# Supplementary material for: Tuning electronic structure of metal-free dual-site catalyst enables exclusive singlet oxygen production and in-situ utilization
Source: Nat Commun. 2024 Jul 10;15:5771. doi: 10.1038/s41467-024-50240-0 (PMC11535063; doi:10.1038/s41467-024-50240-0)
Supplement: Supplementary file 1 — Supplementary Information [file 41467_2024_50240_MOESM1_ESM.pdf]

Supplementary Information for

Tuning Electronic Structure of Metal-Free Dual-Site Catalyst Enables Exclusive Singlet Oxygen Production and In-Situ Utilization

Chao-Hai Gu[1†], Song Wang[2†], Ai-Yong Zhang[1,3*], Chang Liu[1], Jun Jiang[2*], Han-Qing Yu[1*]

[1]CAS Key Laboratory of Urban Pollutant Conversion, Department of Environmental Science and Engineering, University of Science and Technology of China, Hefei, 230026, China

[2]Hefei National Research Center for Physical Sciences at the Microscale, School of Chemistry and Materials Science, University of Science and Technology of China, Hefei 230026, China

[3]Anhui Engineering Laboratory for Rural Water Environment and Resources, School of Civil Engineering, Hefei University of Technology, Hefei, 230009, China

This PDF file includes:

Supplementary text

Figures S1 to S49

Tables S1 to S6

SI References

Supplementary Methods

Theoretical calculation methods

The first-principles DFT calculations were performed using the projector-augmented wave (PAW) method and the Perdew-Burke-Ernzerhof (PBE) exchange-correlation functional as implemented in the Vienna Ab-initio Simulation Package (VASP v5.4.4)[1-3]. To describe the effects of the long-range van der Waals interactions, the semi-classical dispersion correction scheme (DFT-D3) was used[4]. The plane-wave energy cutoff was 450 eV. The gamma point was taken as the k-point. The charge transfer was calculated by using Bader charge method and VASPKIT script[5, 6].

The bonding energies (Eb) were calculated according to the following equation:

$$Eb = E_{sub+mol} - E_{sub} - E_{mol} \quad (S1)$$

where $E_{sub+mol}$ and E_{sub} represent the total energy of substrates with and without PMS or phenol molecule, and E_{mol} represents the total energy of PMS or phenol molecule.

The atomic coordinates of the optimized computational models were provided in Supplementary data 1.

Preparation of control sample NFC/M-HF

To exclude the possible impact of residual Si, Al, and Na on the catalytic performance of NFC/M, the synthesized material underwent an acid-washing procedure. Specifically, 500 mg of NFC/M was added to 60 mL of 1 M hydrofluoric acid (HF) and ultrasonicated for 3 min. This was followed by continuous magnetic stirring for 4 h to ensure thorough acid washing (Supplementary Table 3). The mixture was then vacuum

filtered and washed eight times with deionized water until the effluent pH became neutral. Finally, the washed catalyst was dried at 60 °C for 12 h. The resulting material was designated as NFC/M-HF.

DMA measurement

9,10-Dimethylanthracene (DMA, 50.0 μM) was reacted in a 50.0 wt.% acetonitrile-water solution. The decomposition of DMA was monitored using a UV-vis spectrometer (Shimadzu Co., Japan) and a three-dimensional fluorescence spectrometer (Horiba Co., Japan). The reaction products were identified using an ultra-performance liquid chromatography coupled with high-resolution mass spectrometry (Orbitrap Fusion, Thermo Fisher Inc., USA). The mass spectrometry signals for DMA and 9,10-dimethylanthracene-endoperoxide (DMA-O_2) were recorded in the mass range of 100-450 m/z using an electrospray ion source in a positive ion mode. These signals were utilized to determine the retention times for DMA and DMA-O_2. The conversion of DMA to DMA-O_2 was quantified by measuring the absorbance at 258 nm using the molar absorption coefficients for DMA and DMA-O_2 at this wavelength.

In situ Raman analysis

In situ Raman spectroscopy observation was conducted using a confocal microscopic Raman spectrometer (LABRAM HR EVO, Horiba Co., Japan) with a 532 nm excitation wavelength. For analysis, the NFC/M catalyst was formed into discs with approximately 1.0 mm thickness and 12.6 mm in diameter. Drops of either a 1.0 g/L

phenol solution or a 50.0 mM PMS solution were applied to these discs. The Raman spectra were then collected over a range of 700.0 to 1200.0 cm^{-1} at a resolution of 2.0 cm^{-1}.

Electrochemical measurements

Electrochemical experiments, including linear sweep voltammetry (LSV), chronoamperometry (i-t), and open circuit potential tests (OCPT), were conducted using a workstation (CHI760E, Chenhua Co., China). In the tests a standard three-electrode system in a 0.1 M Na_2SO_4 electrolyte solution was employed. The system comprised a saturated Ag/AgCl reference electrode, a platinum wire counter electrode, and a glassy carbon electrode coated with the catalyst as the working electrode. To prepare the working electrode, a catalyst ink was prepared by ultrasonically blending 5.0 mg of the catalyst with 50.0 μL of a 5.0 wt.% Nafion dispersion and 950.0 μL of ethanol. Subsequently, 10.0 μL of this ink was applied to a freshly polished glassy carbon electrode surface. Chronoamperometry was conducted at open-circuit potential. To explore interactions among the catalyst, oxidant, and pollutant, PMS and phenol were sequentially added to the electrolyte at specified intervals in various sequences, achieving final concentrations of 0.65 mM for PMS and 20.0 $mg \cdot L^{-1}$ for phenol.

Supplementary Notes

Supplementary Note 1. Determination of degradation kinetics

The degradation kinetics of the contaminants followed pseudo-first-order kinetics, with the reaction rate constants (k_{obs}) calculated using the following equation:

$$\ln\left(\frac{C_t}{C_0}\right) = -k_{obs}t \quad \text{(S2)}$$

where C_t is the concentration of the contaminant at time t, and C_0 is the initial concentration at time t_0.

Supplementary Note 2. Thermal operation

In our thermal synthesis process, several crucial objectives were achieved by covering the reaction vial with aluminum foil pierced with three fine holes[7]. These objectives were: 1) Controlled atmospheric exchange: The pierced aluminum foil allowed for limited gas exchange between the interior of the vial and the external environment, which was critical in controlling the atmosphere within the vial during high-temperature pyrolysis. This reaction involved the decomposition of melamine and PTFE organic precursors, releasing gases. Providing a relatively closed space facilitated the mixing and retention of carbon, nitrogen, and fluorine precursors; 2) Release of gaseous product: During heating, it was essential to timely release water vapor and other gaseous by-products like SiF_4, which were instrumental in the in-situ removal of the MMT template; and 3) Prevention of contamination: The foil acted as a barrier, preventing any particles or environmental contaminants from entering the vial during

the heating process. It was especially important to maintain the purity and consistency of the reaction conditions.

To ensure the precision and reproducibility of this setup, the aluminum foil was systematically pierced with a standard 5.0-ml syringe needle. This precise operation guaranteed consistent atmospheric conditions and gas exchange rates during thermal synthesis, minimizing batch-to-batch variations in the synthesis of NFC/M materials.

Supplementary Figures

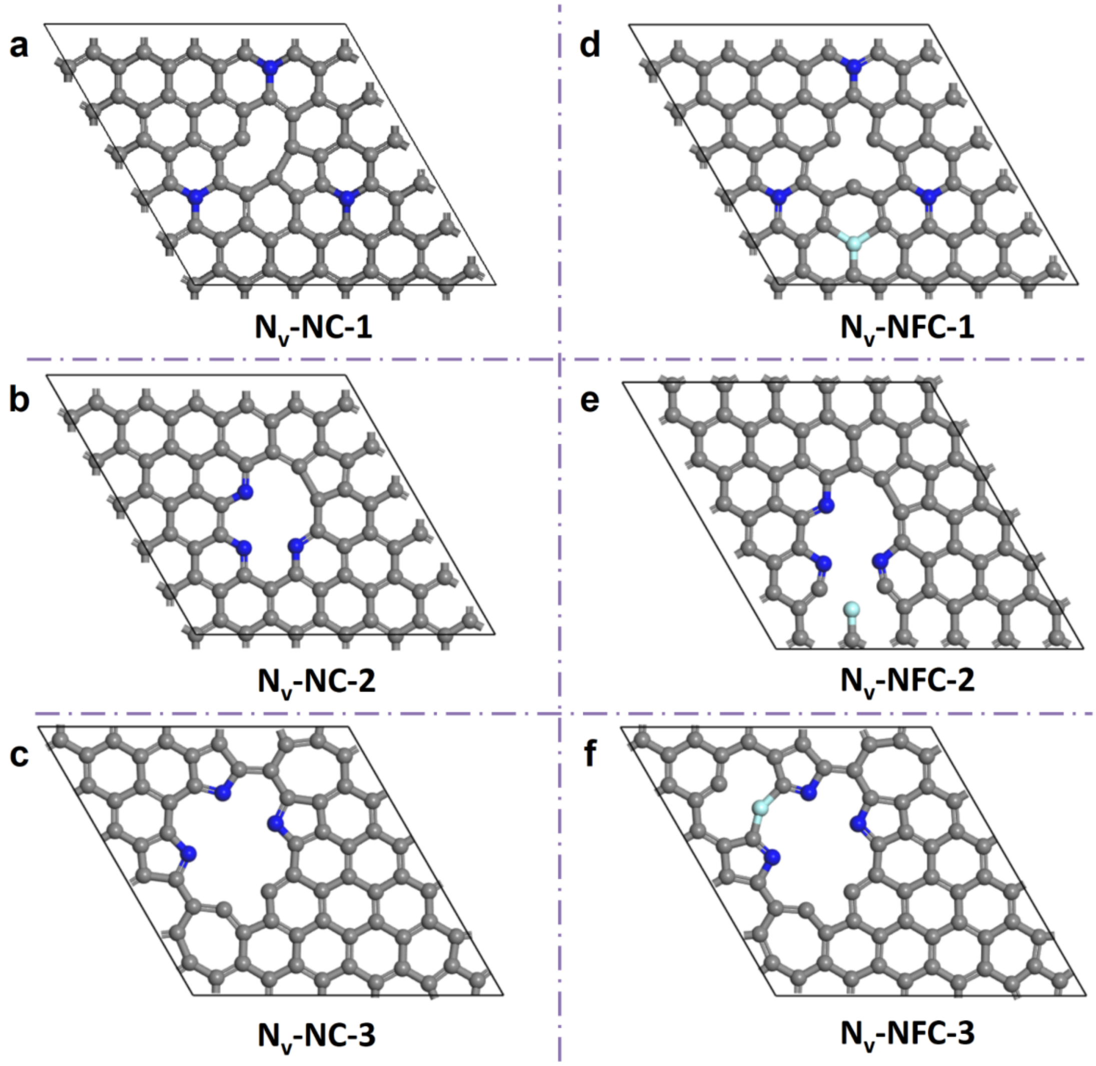

Supplementary Fig. 1 | Configuration of different N vacancy atomic models with and without F doping after DFT optimization. **(a, d)** N_v-NC-1 and N_v-NFC-1, graphitic nitrogen vacancy. **(b, e)** N_v-NC-2 and N_v-NFC-2, pyridinic nitrogen vacancy. **(c, f)** N_v-NC-3 and N_v-NFC-3, pyrrolic nitrogen vacancy. Grey, blue, and cyan spheres represent C, N and F atoms, respectively.

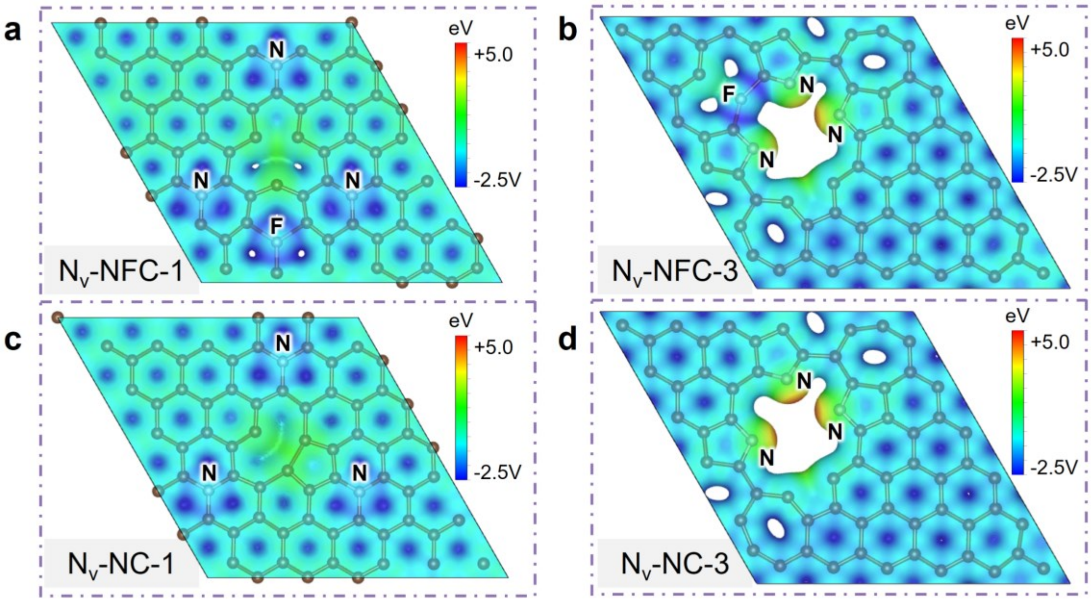

Supplementary Fig. 2 | Electrostatic potential distributions for **(a)** N_v-NFC-1, **(b)** N_v-NFC-3, **(c)** N_v-NC-1 and **(d)** N_v-NC-3.

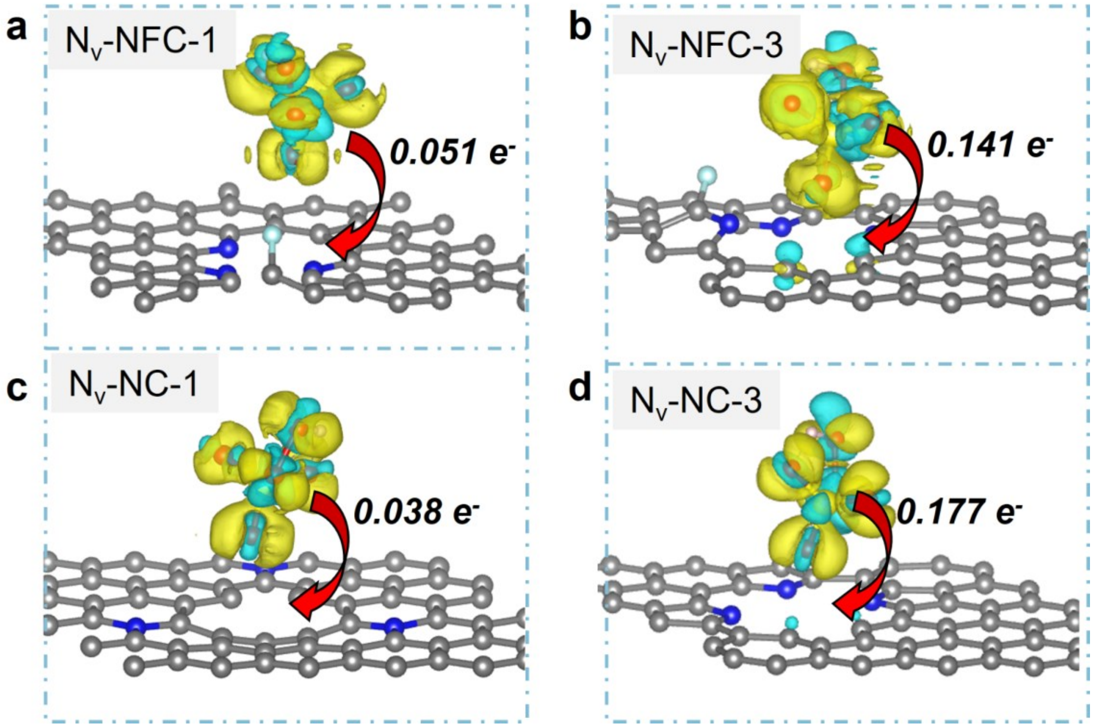

Supplementary Fig. 3 | Difference charge density for PMS adsorption on **(a)** N_v-NFC-1, **(b)** N_v-NFC-3, **(c)** N_v-NC-1 and **(d)** N_v-NC-3. Yellow and cyan regions represent electron accumulation and electron depletion, respectively.

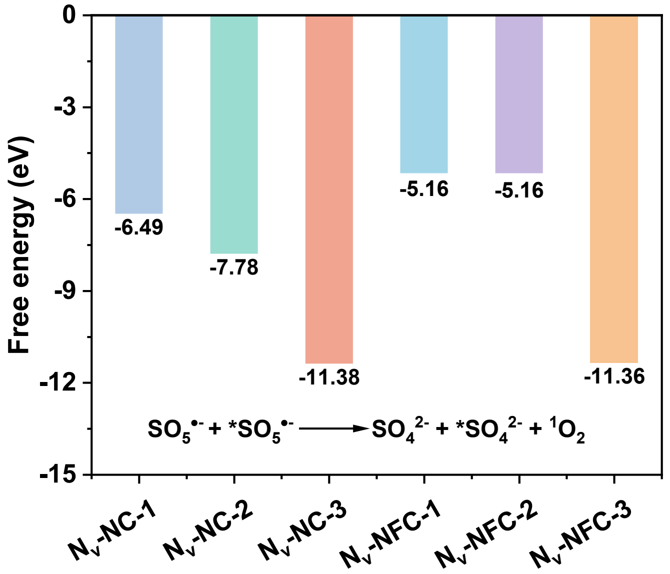

Supplementary Fig. 4 | Free energy of generating singlet oxygen by activating persulfate radical on different sites. The * symbol represents catalytic or adsorption sites.

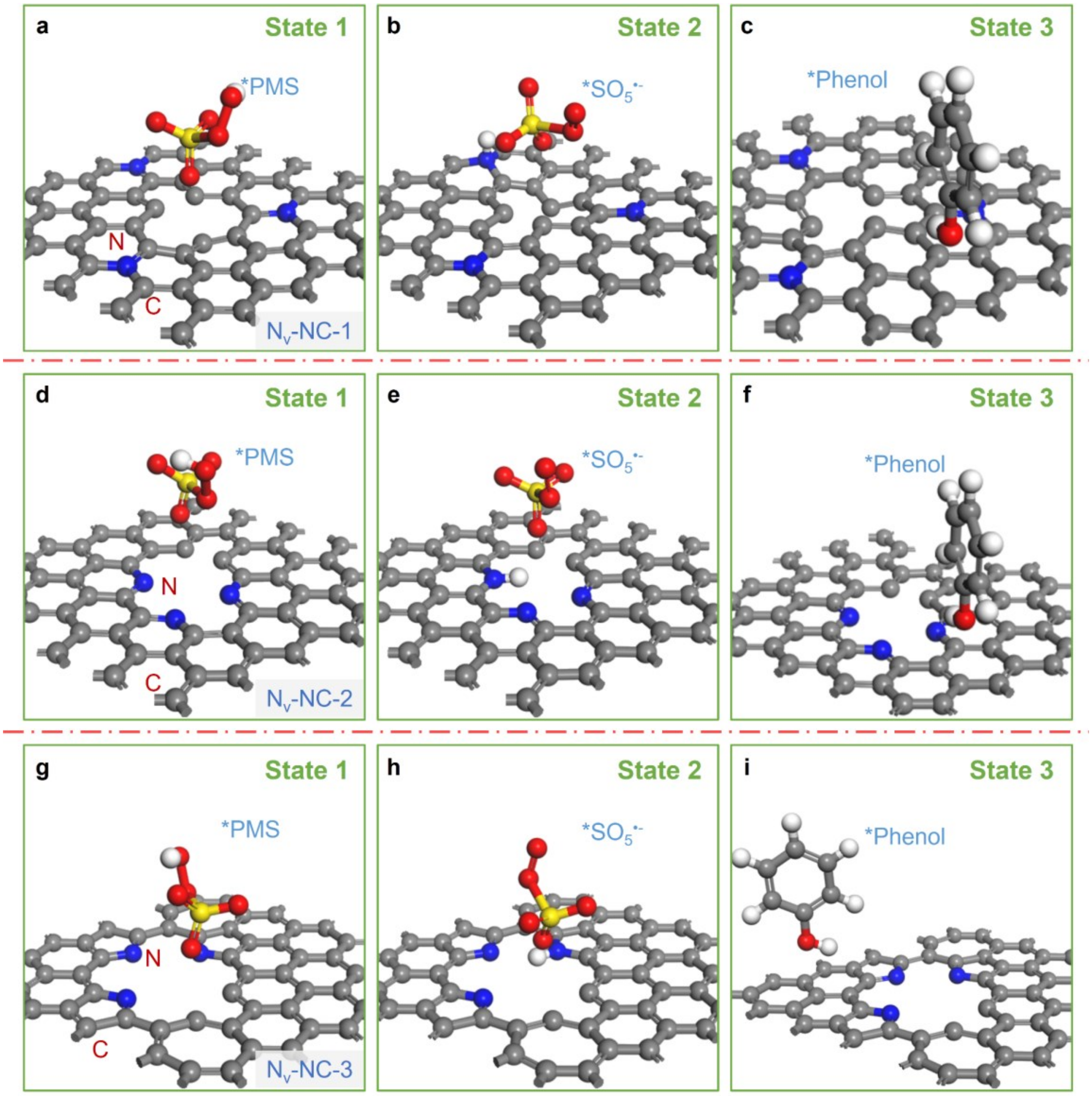

Supplementary Fig. 5 | Optimized configurations of atomic models. **(a, d, g)** PMS adsorbed on N_v-NC-1, N_v-NC-2, and N_v-NC-3. **(b, e, h)** PMS decomposition on N_v-NC-1, N_v-NC-2, and N_v-NC-3. **(c, f, i)** Phenol adsorbed on N_v-NC-1, N_v-NC-2, and N_v-NC-3. Grey, blue, red, yellow, and white spheres represent C, N, O, S and H atoms, respectively. The * symbol represents catalytic or adsorption sites.

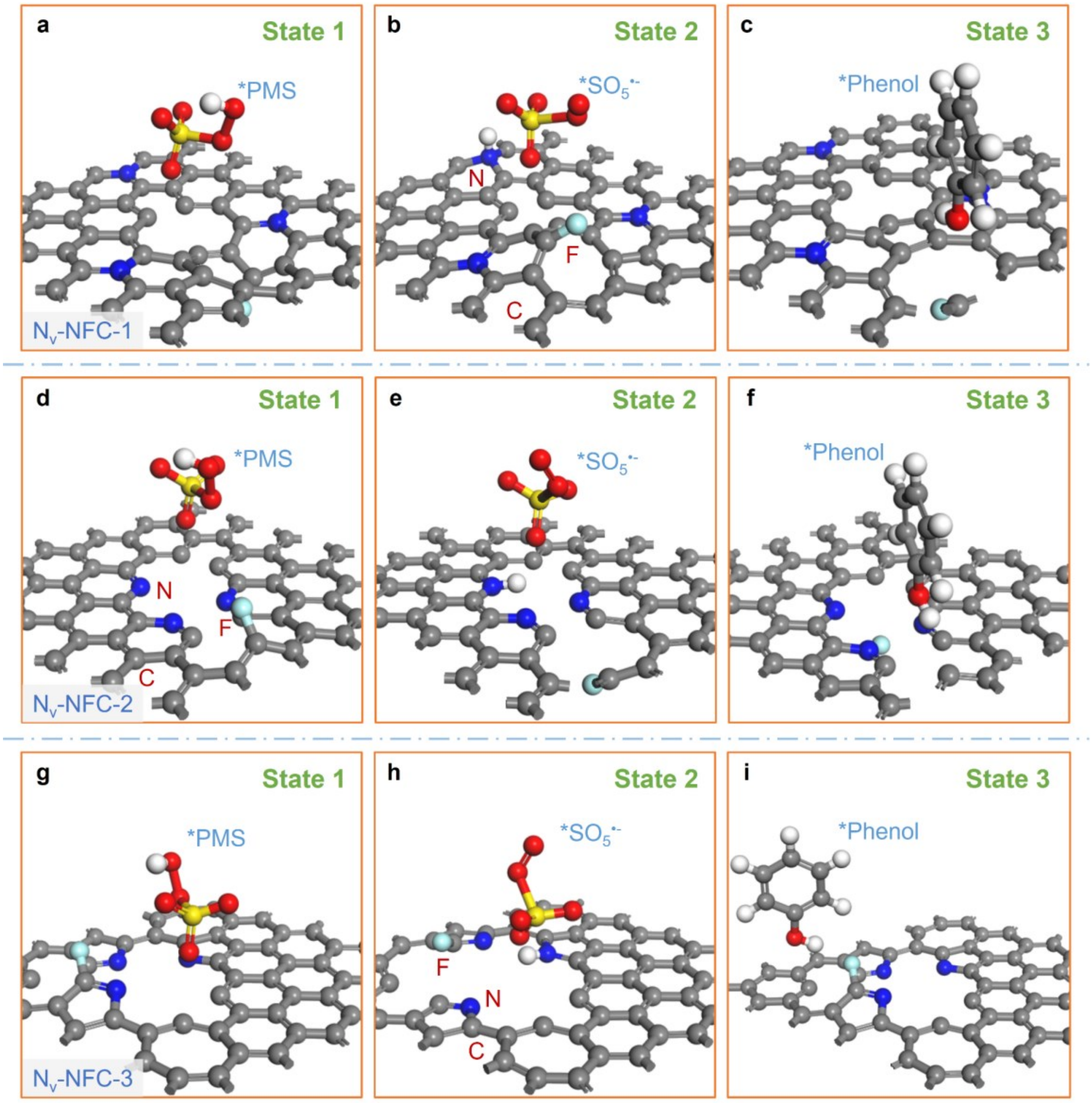

Supplementary Fig. 6 | Optimized configurations of atomic models. **(a, d, g)** PMS adsorbed on N_v-NFC-1, N_v-NFC-2, and N_v-NFC-3. **(b, e, h)** PMS decomposition on N_v-NFC-1, N_v-NFC-2, and N_v-NFC-3. **(c, f, i)** Phenol adsorbed on N_v-NFC-1, N_v-NFC-2, and N_v-NFC-3. Grey, blue, cyan, red, yellow, and white spheres represent C, N, F, O, S and H atoms, respectively. The * symbol represents catalytic or adsorption sites.

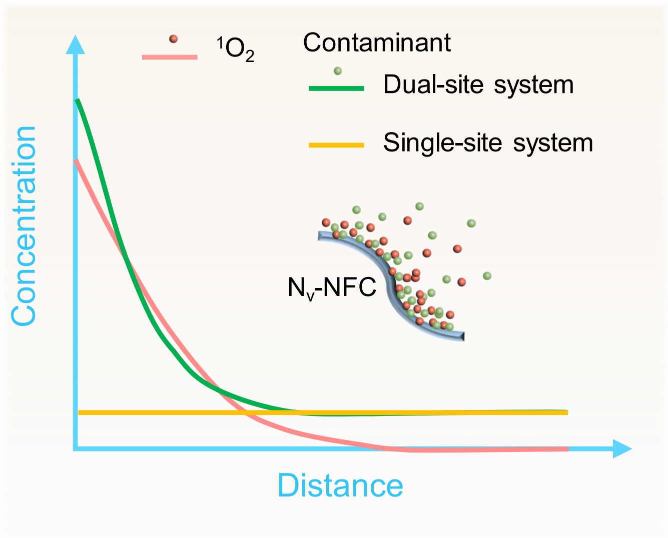

Supplementary Fig. 7 | Schematic diagram of efficient utilization of $^1O_2$ rendered by the dual-site model.

Notes for Supplementary Fig. 7

The term "concentration" specifically denotes the level of reactive oxygen species (here is $^1O_2$) and organic pollutants at varying distances from the catalyst surface (dual-site catalyst N_v-NFC or single-site catalyst N_v-NC) and their accessibility to each other during Fenton-like reactions.

For the spatial distribution of $^1O_2$, its concentration rapidly decreased with the increasing distance from the catalyst surface due to its brief lifetime in an aqueous medium (2.9 ~ 4.6 μs)[8-10]. This decline was primarily due to the nonradiative relaxation of $^1O_2$, leading to the rapid physical deactivation and further compounded by mass transfer limitations inherent in heterogeneous reactions[11, 12].

As for the distribution of pollutants, the systems with a single reactive site (Nv-NC) exhibited a uniform distribution throughout the solution, as there were no adsorptive sites on the catalyst to concentrate the pollutants (Fig. 1g). This resulted in inefficient utilization of $^1O_2$ during Fenton-like catalysis[12, 13]. In contrast, in the metal-free dual-site system (N_v-NFC), the F-C Lewis acid sites functioned as adsorptive sites, which effectively attracted and concentrated pollutants at the catalyst surface. This configuration enhanced the proximity and availability of reactive species, significantly improving the in-situ utilization of $^1O_2$. Due to the exclusive production of $^1O_2$ at the nitrogen vacancy sites (NVs) triggered by PMS activation and its neighboring utilization at the F-C Lewis sites for pollutant degradation, $^1O_2$ was utilized as the non-radical reactive oxygen species for comparison between single-site and dual-site systems in Fenton-like catalysis (Figs. 1 and 4). The dual-site system also supported other reactive oxygen species with high reactivity and short lifetimes in heterogeneous Fenton-like processes.

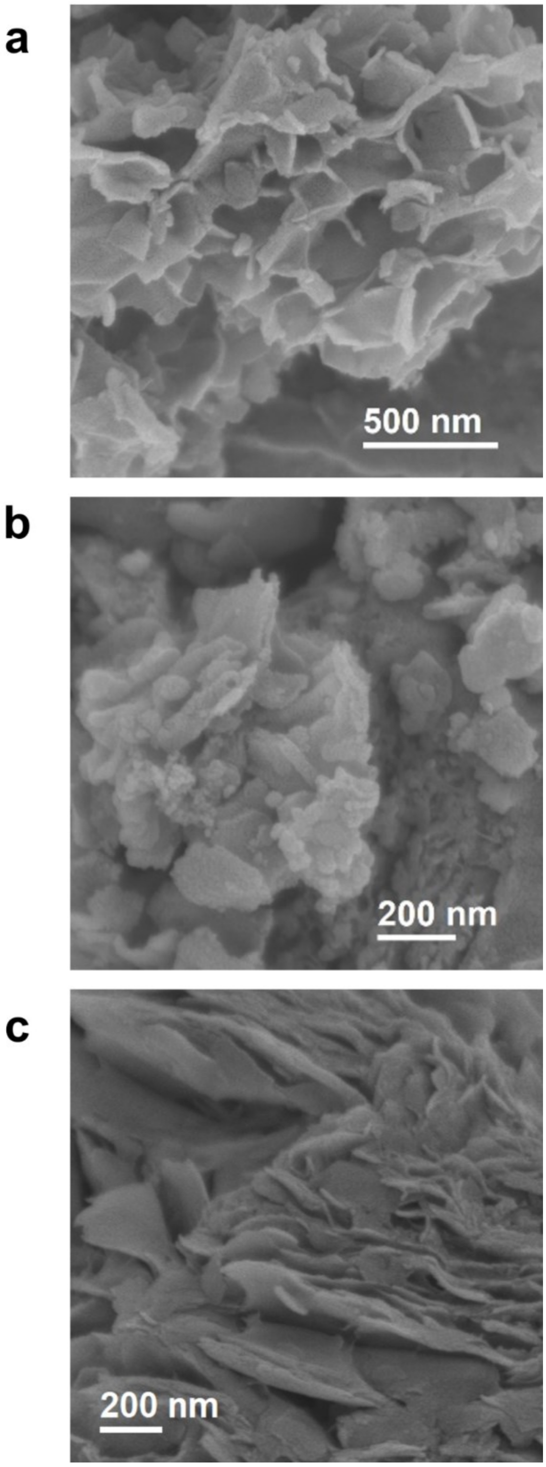

Supplementary Fig. 8 | SEM images of **(a)** NFC/M, **(b)** NFC and **(c)** NC/M.

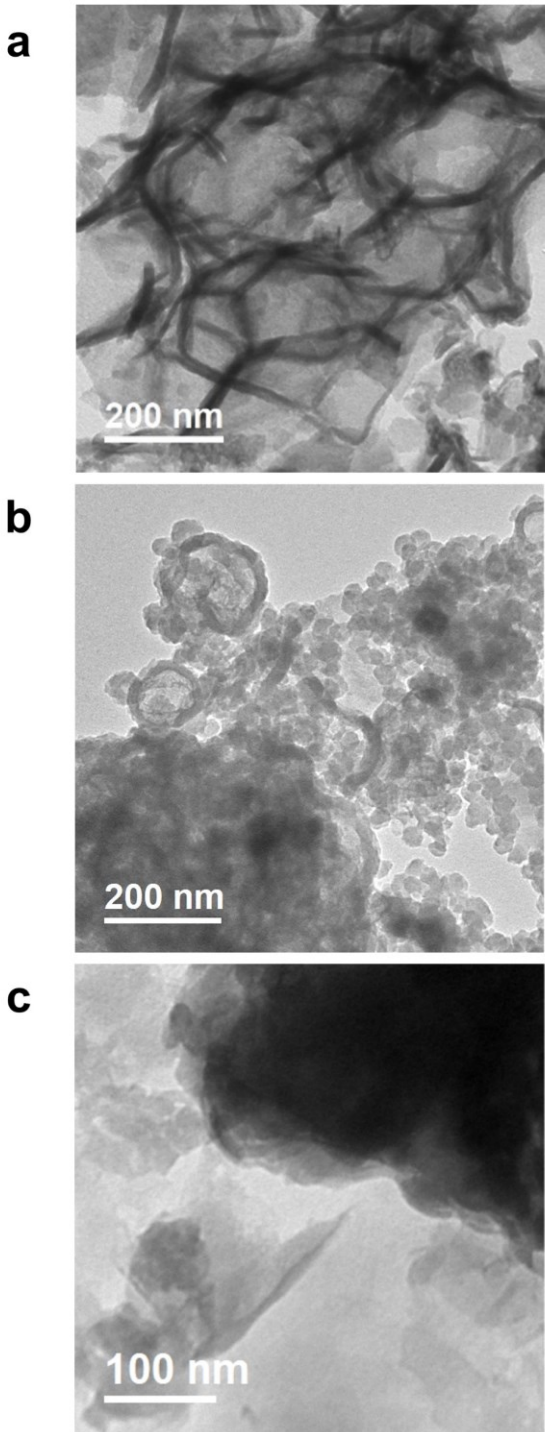

Supplementary Fig. 9 | TEM images of **(a)** NFC/M, **(b)** NFC and **(c)** NC/M.

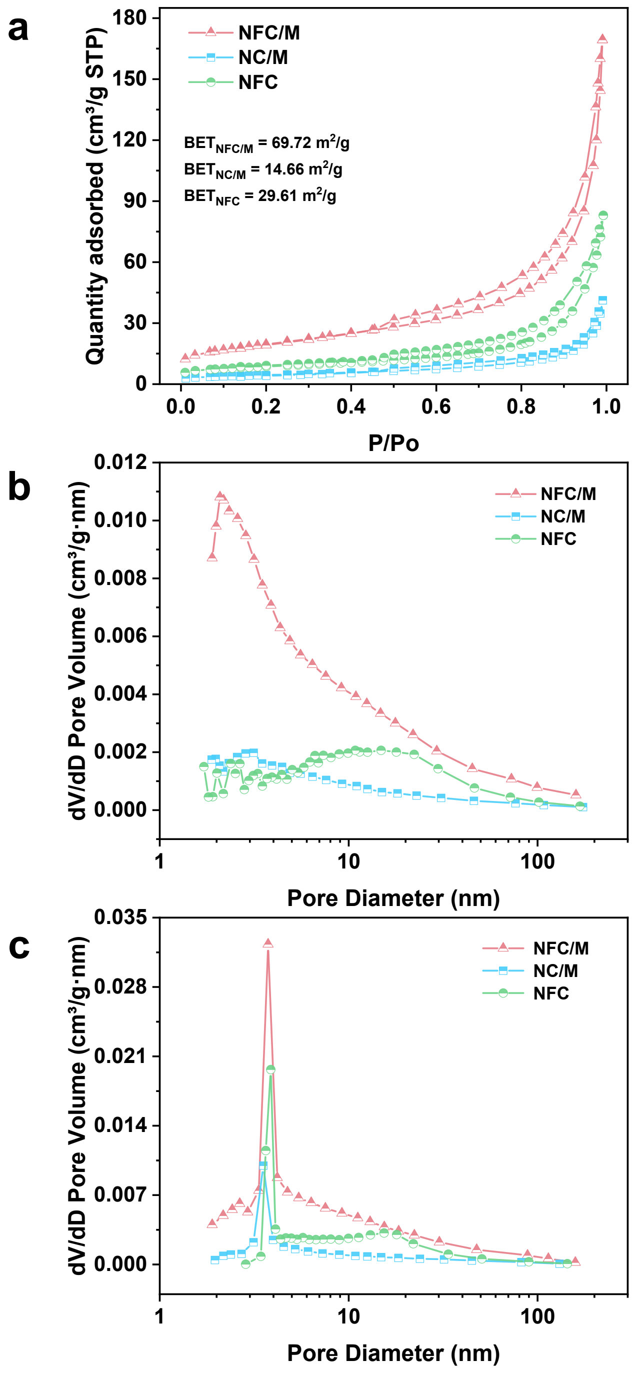

Supplementary Fig. 10 | Specific surface area. **(a)** N_2 adsorption and desorption isotherms, **(b)** adsorption-pore size distribution, and **(c)** desorption-pore size distribution for NFC/M, NC/M and NFC.

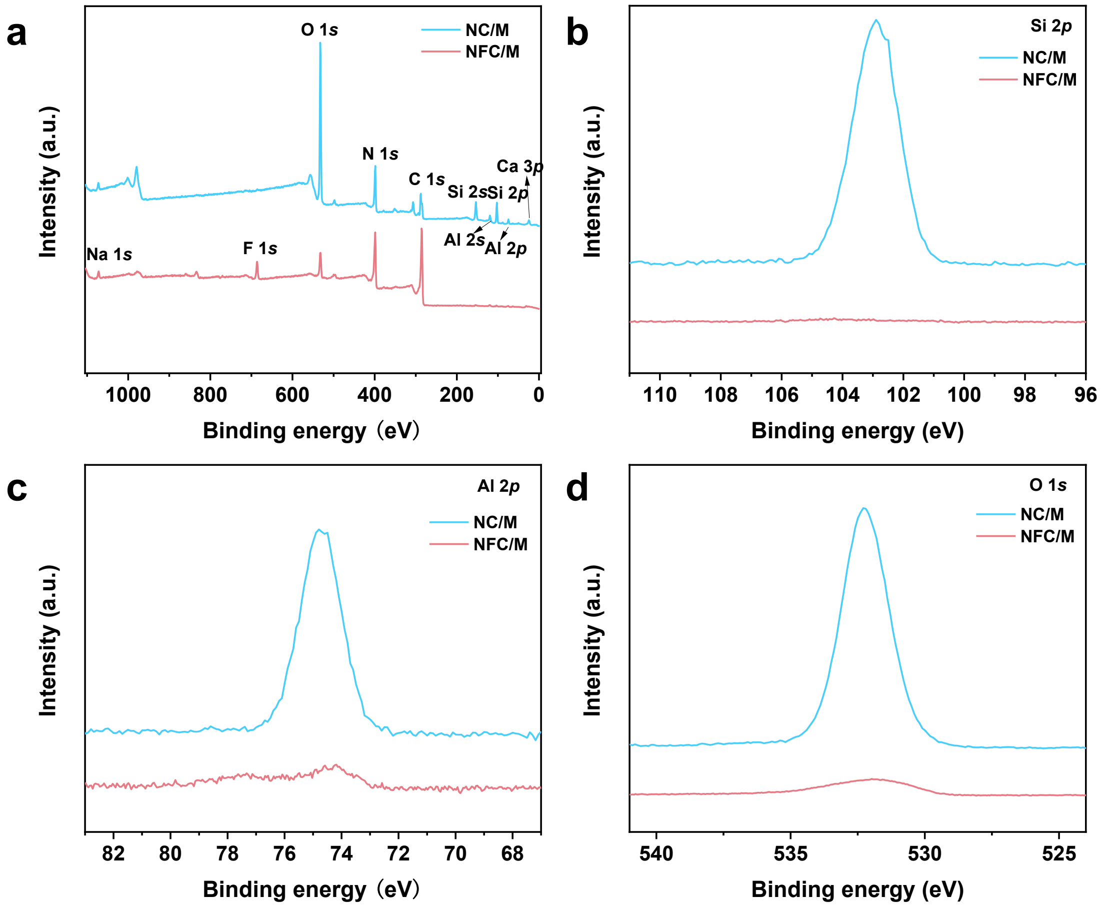

Supplementary Fig. 11 | XPS spectra of NFC/M and NC/M. **(a)** Survey XPS spectra indicated with main elemental contributions. **(b)** Si 2*p* spectra, **(c)** Al 2*p* spectra, and **(d)** O 1*s* spectra. Significant reductions in elements like Si, Al and O-typical in MMT-were observed in NFC/M.

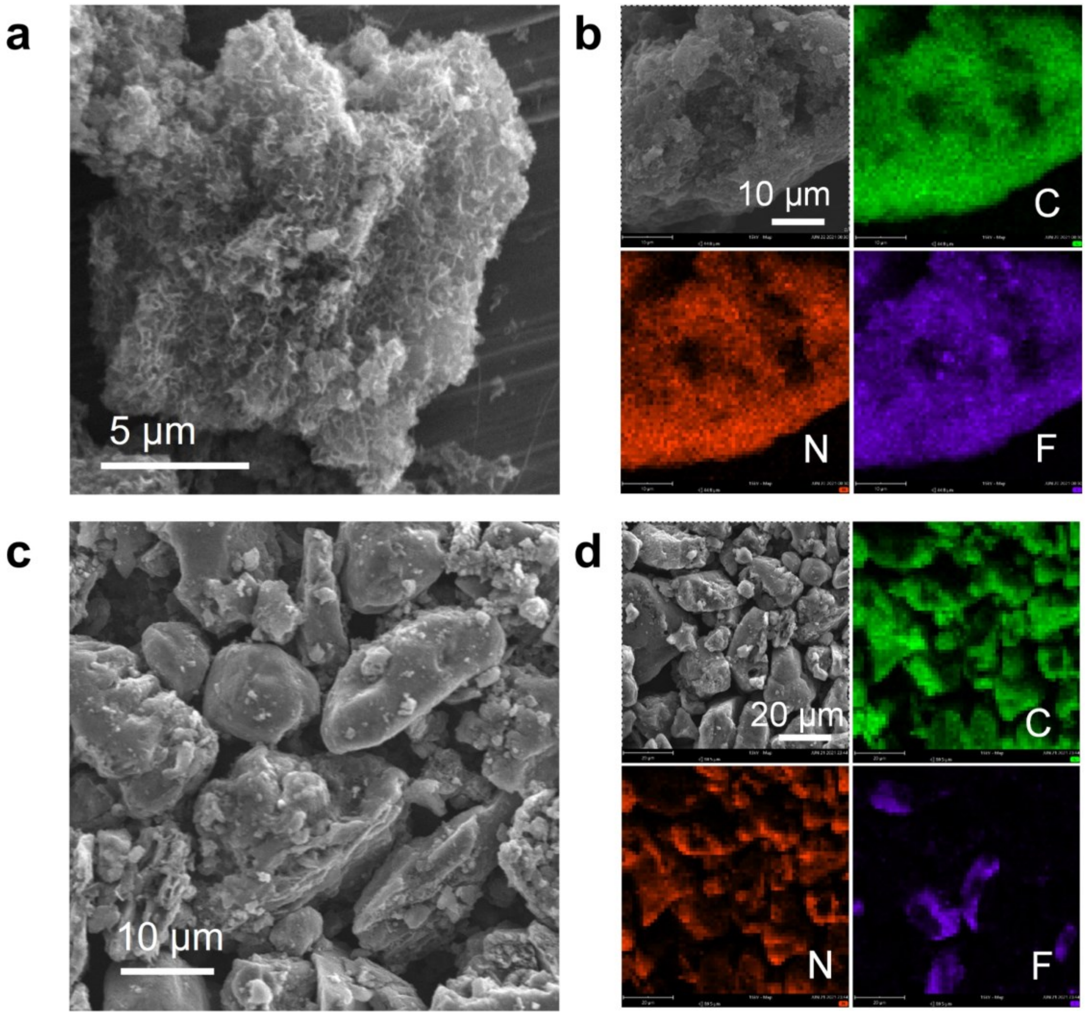

Supplementary Fig. 12 | SEM images and corresponding EDS elemental mappings of **(a, b)** NFC/M, and **(c, d)** NFC.

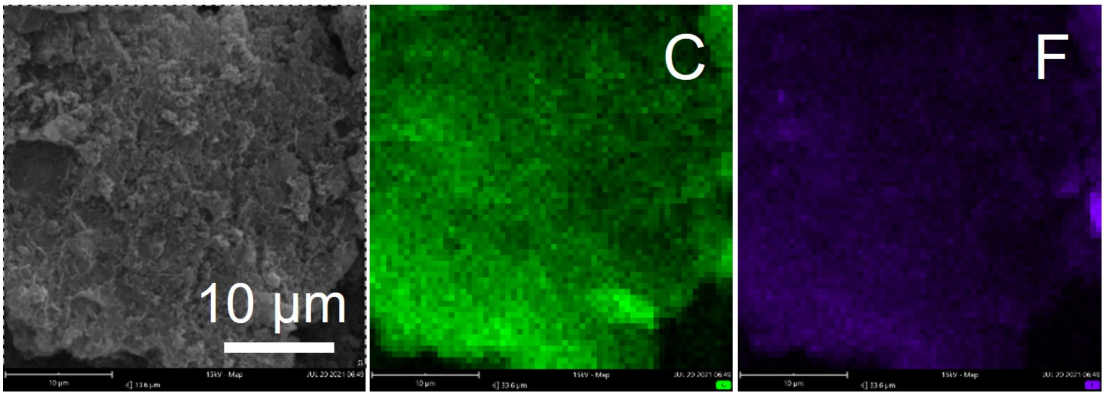

Supplementary Fig. 13 | SEM image and corresponding EDS elemental mapping of FC/M.

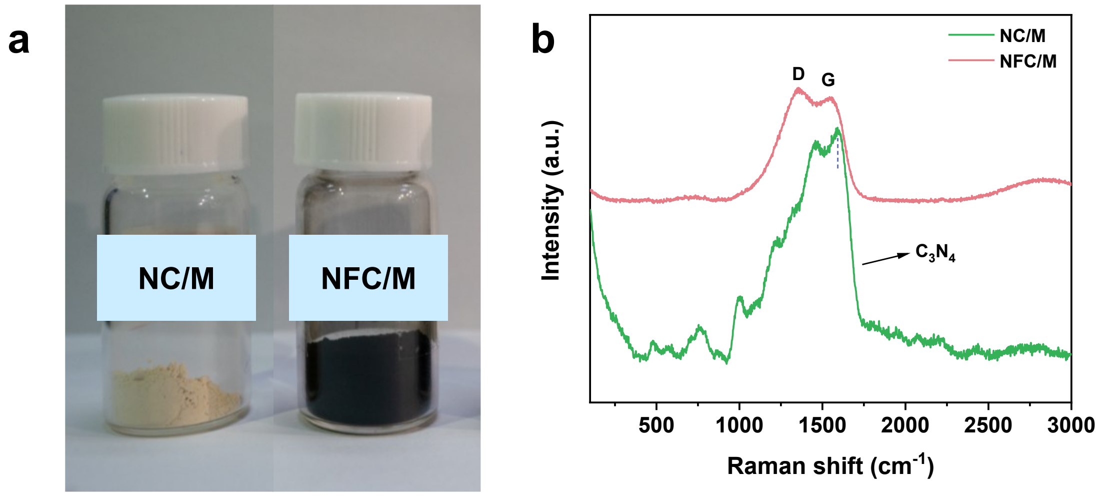

Supplementary Fig. 14 | (a) Photographs of NC/M and NFC/M. **(b)** Raman spectra of NC/M and NFC/M.

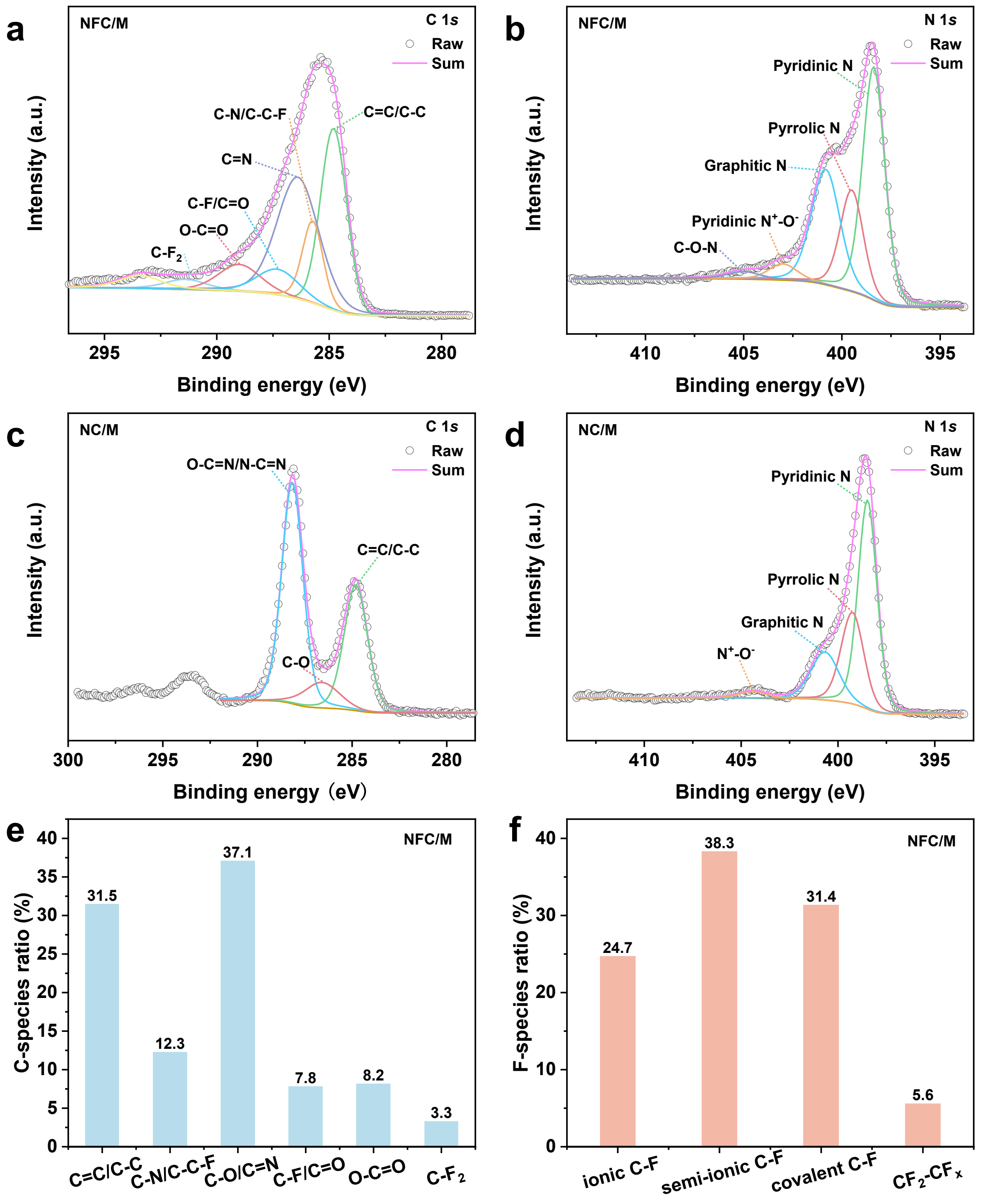

Supplementary Fig. 15 | High-resolution XPS spectra of NFC/M and NC/M. **(a, b)** C 1*s* spectra and N 1*s* spectra of NFC/M. **(c, d)** C 1*s* spectra and N 1*s* spectra of NC/M. **(e, f)** Relative ratios of deconvoluted C species and F species in NFC/M calculated from the high-resolution C 1*s* and F 1*s* XPS spectra.

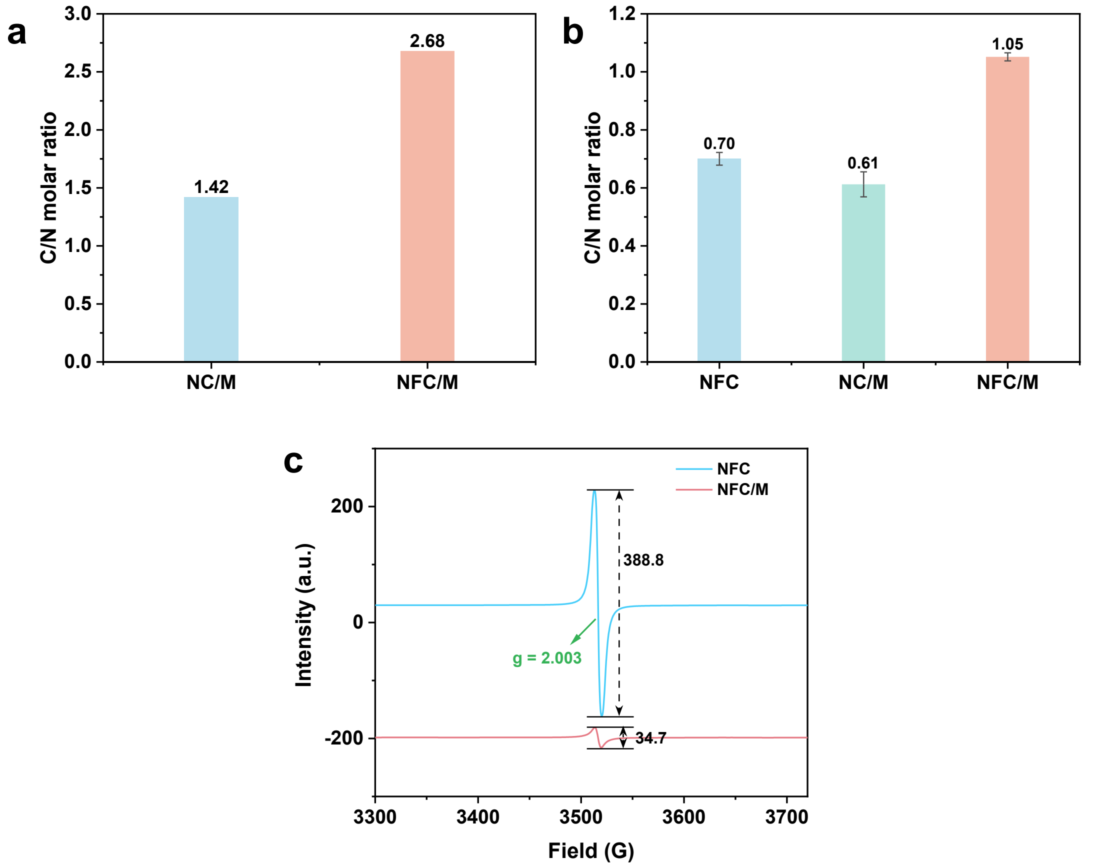

Supplementary Fig. 16 | Elemental analysis and structural defect. **(a)** The C/N molar ratio based on the XPS results. **(b)** The C/N molar ratio based on the SEM-EDS results. Data are presented as mean values ± SD (n = 3). **(c)** EPR spectra of NFC/M and NFC measured at room temperature.

Notes for Supplementary Fig. 16

The composite EPR signal at g=2.003 indicated the presence of either persistent carbon-centric free radicals or defective nitrogen vacancies. Despite equal precursor ratios for carbon, nitrogen, and fluorine, NFC showed a substantially lower surface C/N ratio than NFC/M, as detailed in the XPS and EDS results (Supplementary Fig. 16a, b). Such a lower ratio suggested minimal nitrogen loss during the synthesis of NFC, which lacked the protective MMT template, resulting in fewer nitrogen vacancies in the carbon matrix. This finding was corroborated by bulk phase elemental analysis (EA), which aligns with surface measurements and provides a detailed view of the catalysts' elemental composition and vacancy distribution (Fig. 3c).

Moreover, Raman spectroscopic results (Fig. 3b) support the conclusion that fewer nitrogen vacancies were present in NFC. However, the EPR characterization (Supplementary Fig. 16c) revealed a significantly strong unpaired electron signal—11.2 times higher than that in NFC/M—highlighting the absence of MMT's buffering effect. The lack of this protection allowed the corrosive gases from fluorine precursor

decomposition to etch and structurally damage the NFC surface, leading to an increased formation of carbon radicals.

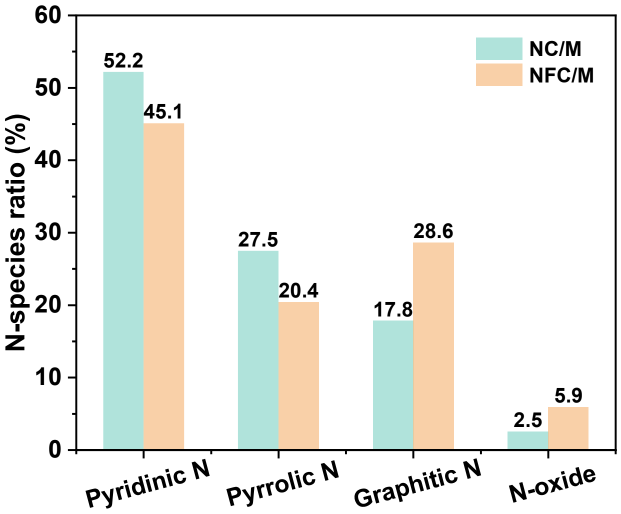

Supplementary Fig. 17 | Relative ratio of deconvoluted N species in NFC/M and NC/M calculated from their high-resolution N 1*s* XPS spectra. The data revealed a large decrease in pyridinic N and pyrrolic N in NFC/M compared to NC/M. Conversely, the relative abundance of graphitic N and N-oxide increased. These findings indicate that the fluorine-incorporation selectively induced the formation of pyridinic and pyrrolic nitrogen vacancies.

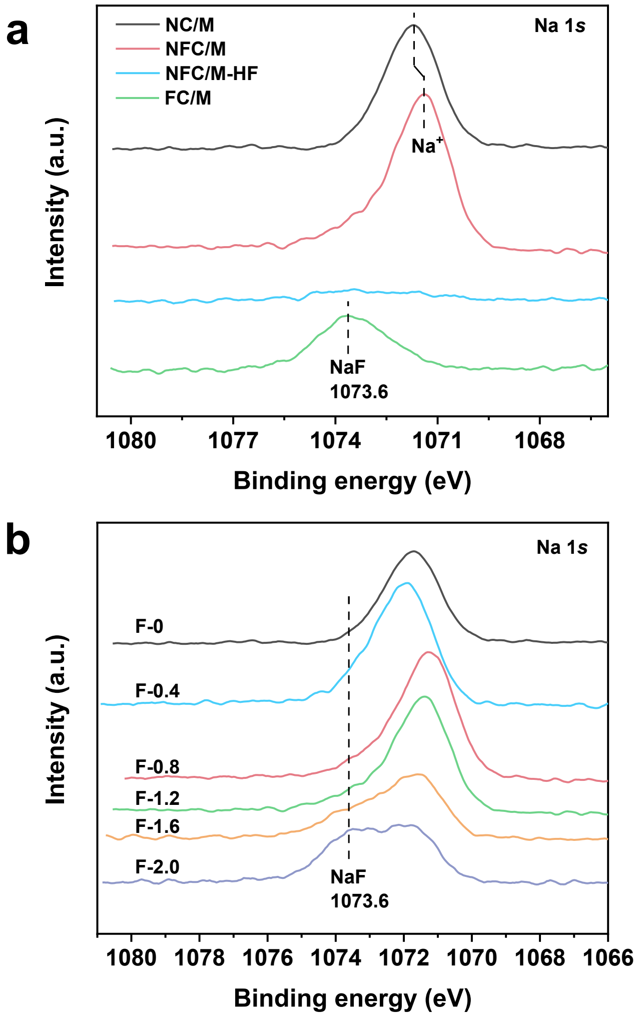

Supplementary Fig. 18 | Comparison in the binding energy and chemical speciation of sodium. **(a)** For NC/M, NFC/M, NFC/M-HF and FC/M based on Na 1*s* XPS spectra. **(b)** For different NFC/M catalysts prepared with various fluorine precursors based on Na 1*s* XPS spectra.

Notes for Supplementary Fig. 18

XPS analysis indicates that there was no significant formation of NaF under standard conditions (Supplementary Fig. 18a). This absence could be attributed to the reductive atmosphere created by the decomposition of melamine, which facilitated the formation of HF, rather than NaF. HF was then consumed or buffered by the MMT template (Supplementary Figs. 19 and 20). However, in the samples synthesized with higher dosages of PTFE, or without melamine, we observed a gradual increase in NaF formation (Supplementary Fig. 18). This result suggests that in environments lacking the reductive atmosphere, excess fluorine was free to react with available Na, forming NaF, further highlighting the selectivity of fluorine binding under varying synthetic conditions.

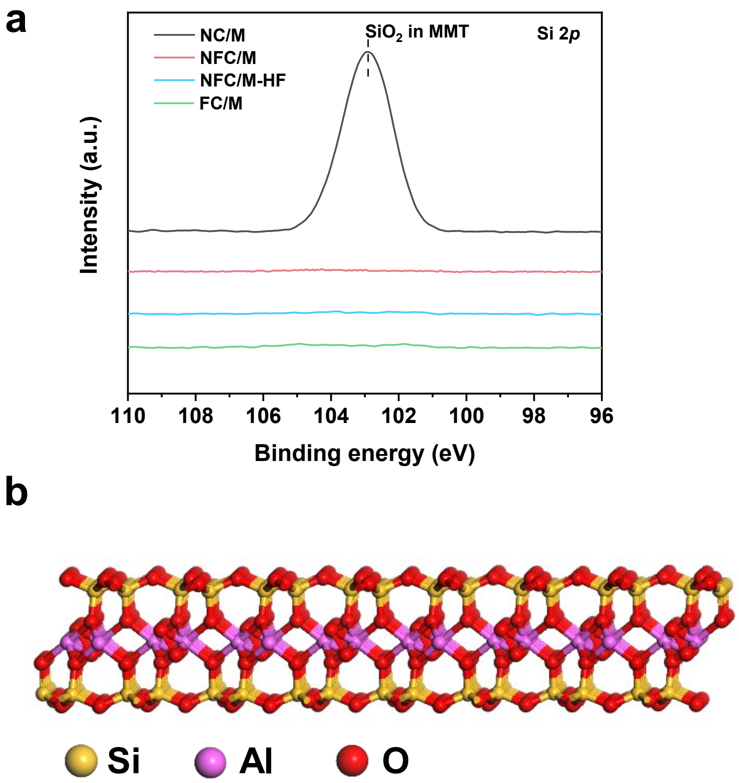

Supplementary Fig. 19 | (a) Comparison of the residual SiO_2 content in the different catalyst samples of NC/M, NFC/M, NFC/M-HF and FC/M prepared under various conditions. **(b)** The atomic representation of used layer-stacked MMT template.

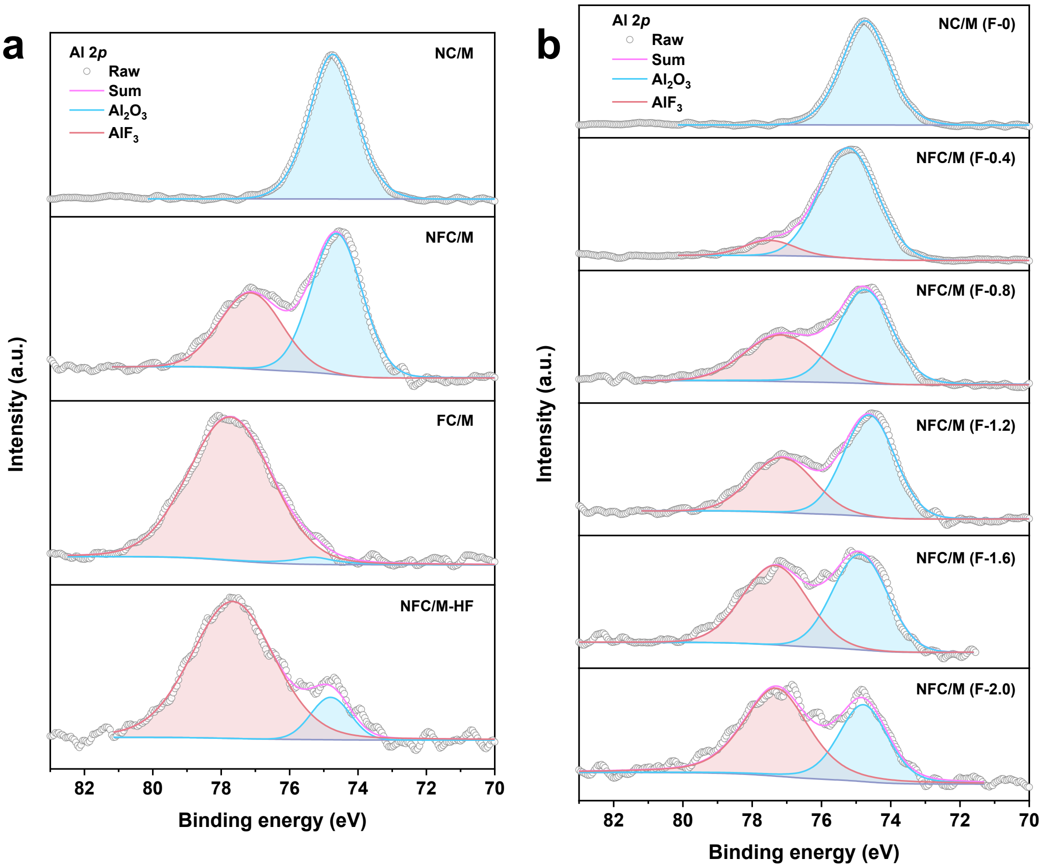

Supplementary Fig. 20 | Comparison of the binding energy and chemical speciation of aluminum. **(a)** For NC/M, NFC/M, NFC/M-HF and FC/M based on Al 2*p* XPS spectra. **(b)** For different NFC/M prepared with various fluorine precursors based on Al 2*p* XPS spectra.

Notes for Supplementary Figs. 19 and 20

In the process of synthesizing NFC/M, the montmorillonite (MMT) template, composed of a SiO_2-sandwiching-Al_2O_3 layered structure, played a crucial role (Figs. 2, 3 and Supplementary Figs. 19). During thermal preparation, the highly reactive fluorine intermediates, derived from the decomposition of polytetrafluoroethylene (PTFE), interacted preferentially with the outer SiO_2 layers of MMT. This interaction led to the formation of SiF_4, facilitating the in-situ removal of SiO_2 ($SiO_2 + 4HF \rightarrow SiF_4\uparrow + 2H_2O$). Subsequently, the inner Al_2O_3 layers provided a buffering effect, consuming any excess corrosive fluorine to form AlF_3, which was also confirmed by a minimal residual presence of Si in XPS analysis (Supplementary Figs. 19a) and the formation of some AlF_3 in the catalyst matrix (Supplementary Figs. 20). This staged consumption effectively prevented the corrosion of the carbon structure by acidic fluorine gases (Supplementary Fig. 16).

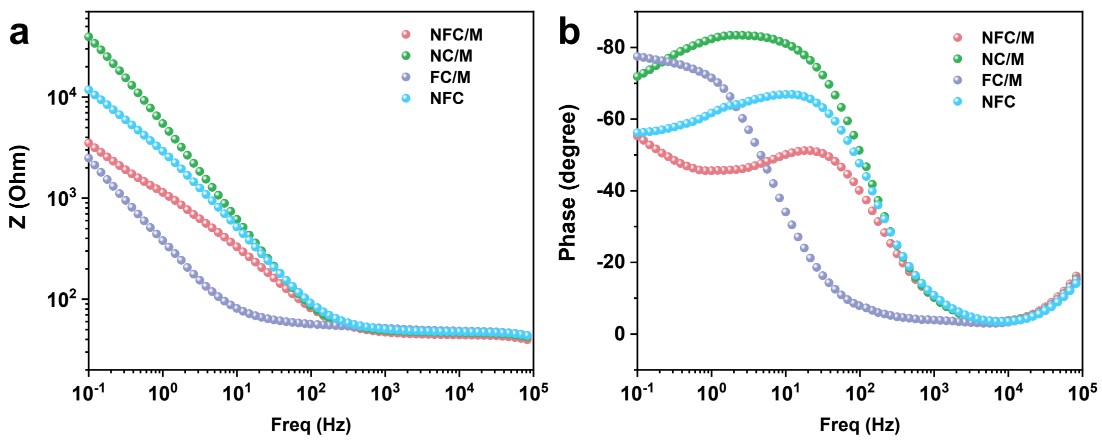

Supplementary Fig. 21 | EIS results of different catalysts. (**a**) Nyquist plot: impedance (Z) vs frequency (Freq). (**b**) Bode plot: phase angle (Phase) vs frequency (Freq).

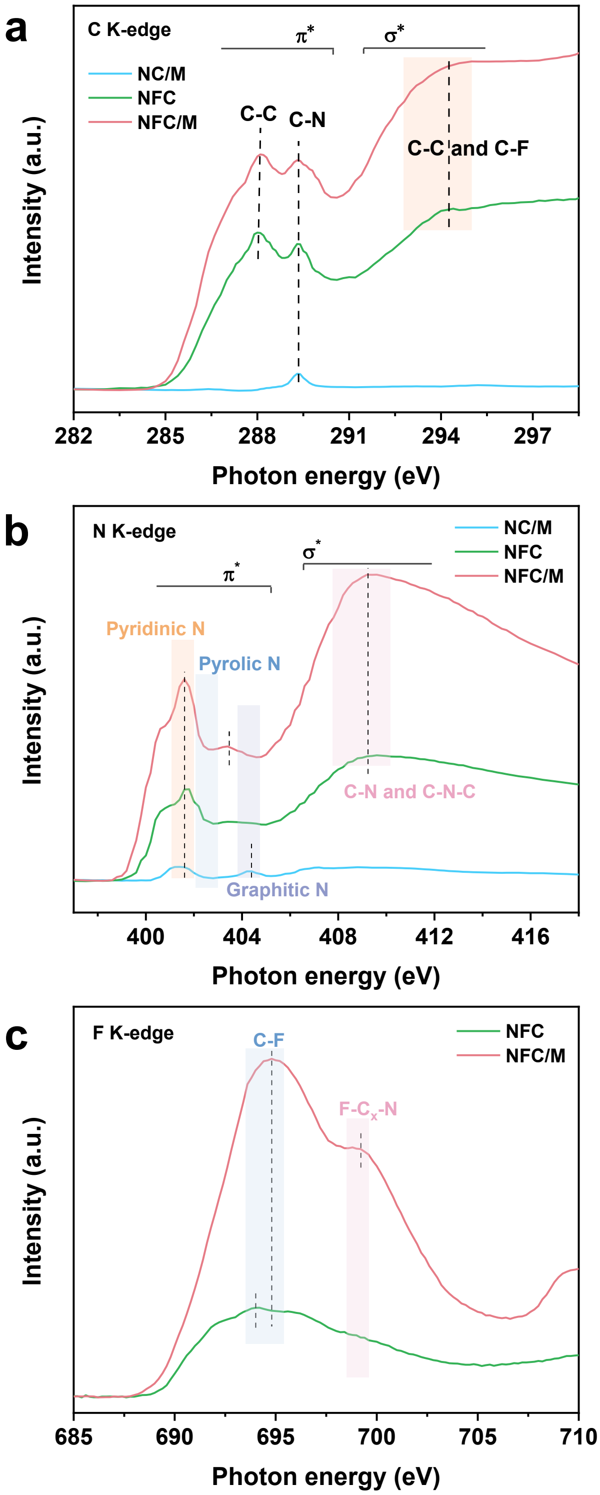

Supplementary Fig. 22 | XAS spectra before normalizing at the **(a)** C K-edge, **(b)** N K-edge, and **(c)** F K-edge of different catalysts. The data demonstrated a significant enhancement in the original XAS signals, from NC/M to NFC, and further to NFC/M, indicating a substantial improvement in the material's conductivity.

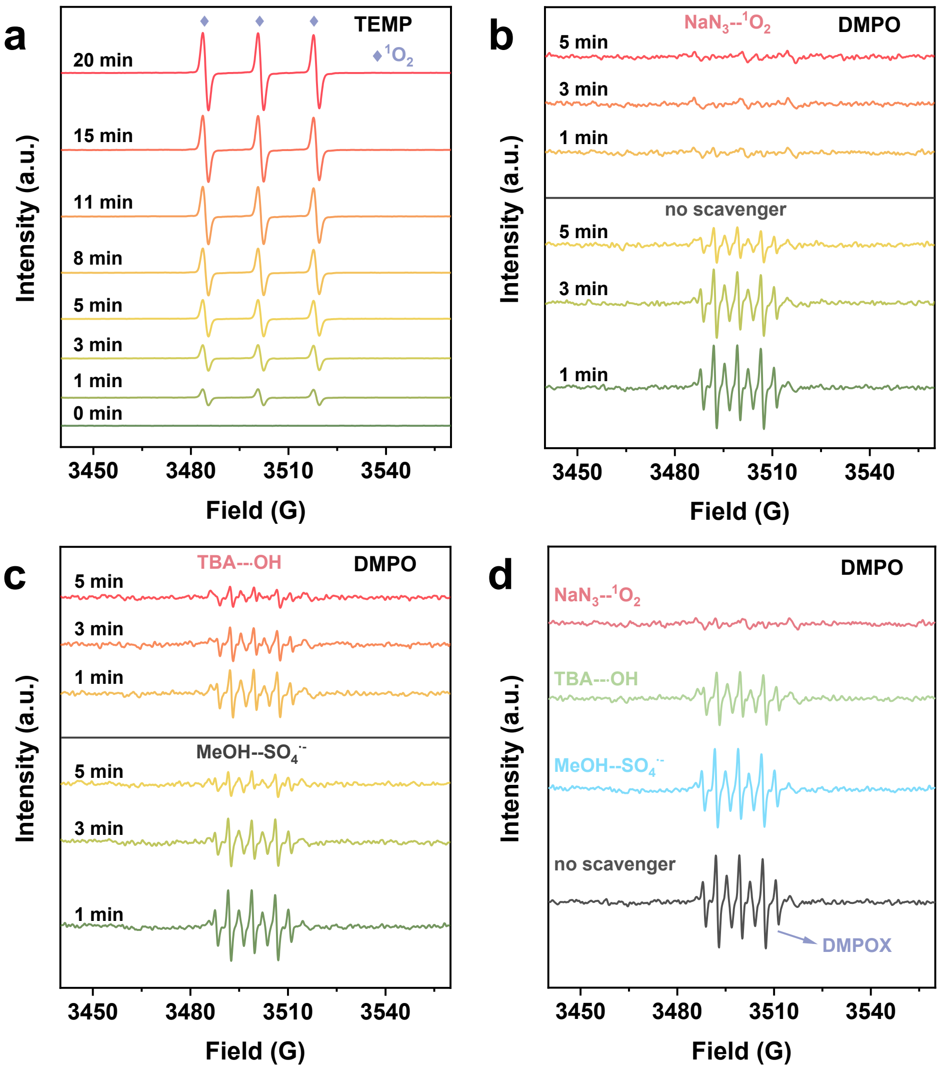

Supplementary Fig. 23 | ROS identification. **(a)** Spin-trapping EPR spectra of TEMP-$^1O_2$ in NFC/M-PMS system. **(b, c)** EPR spectra in NFC/M-PMS system using DMPO as trapping agents, and NaN_3, MeOH and TBA as scavengers. **(d)** Quenching effects of different scavengers on EPR spectra of DMPO-DMPOX in NFC/M-PMS system after 1 min of reaction. Reaction conditions: [catalyst] = 0.2 g·L^{-1}, [PMS] = 0.65 mM, [phenol] = 20.0 mg·L^{-1}; [NaN_3] = 0.2 g·L^{-1}, [MeOH] = [TBA] = 0.5 M; initial pH 7.0, Temp. = 20.0 ± 2.0 °C.

Notes for Supplementary Fig. 23

When DMPO was utilized to detect radicals, no discernible signals of $^{\bullet}OH$ and $SO_4^{\bullet-}$ radicals were observed, only a DMPOX signal was detected. To examine the origin of DMPOX signal, EPR quenching experiments were conducted (Supplementary Fig. 23). These experiments demonstrate that typical radical quenchers did not affect the DMPOX signal. However, NaN_3, a known $^1O_2$ quencher, greatly inhibited the formation of the DMPOX signal. In addition, the presence of NaN_3 showed minimal impact on the consumption of PMS (Supplementary Fig. 24). Thus, the DMPOX signal was originated from the DMPO oxidation by $^1O_2$.

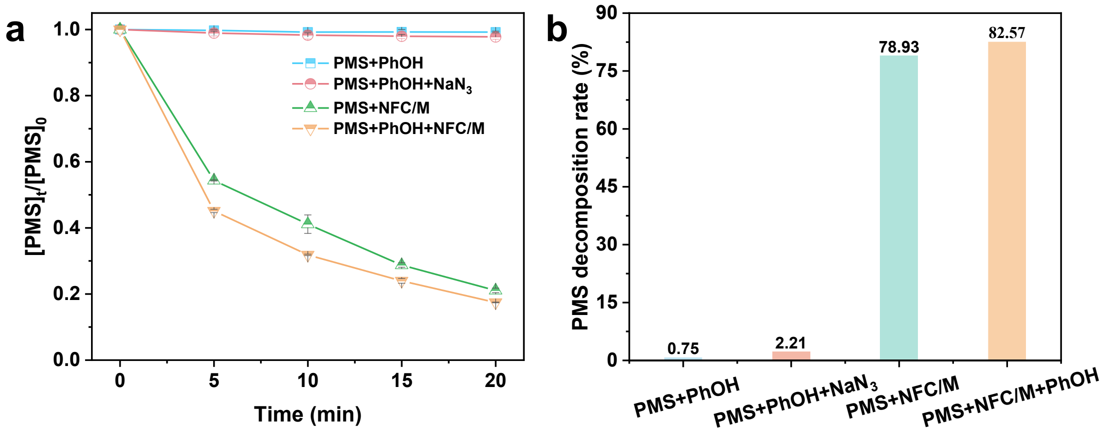

Supplementary Fig. 24 | (a) PMS decomposition in different systems. Data are presented as mean values ± SD (n = 3). **(b)** PMS decomposition efficiency after 20.0 min of reaction. Reaction conditions: [catalyst] = 0.2 g·L^{-1}, [PMS] = 0.65 mM, [phenol] = 20.0 mg·L^{-1}, [NaN_3] = 0.2 g·L^{-1}, Temp. = 20.0 ± 2.0 °C.

Notes for Supplementary Fig. 24

The presence of phenol had a minimal promoting effect on PMS decomposition (Supplementary Fig. 24), and in-situ Raman spectra did not reveal any reactive surface complexes (PMS*; Fig. 4h), effectively ruling out the direct oxidation processes mediated by the catalyst-PMS complex.

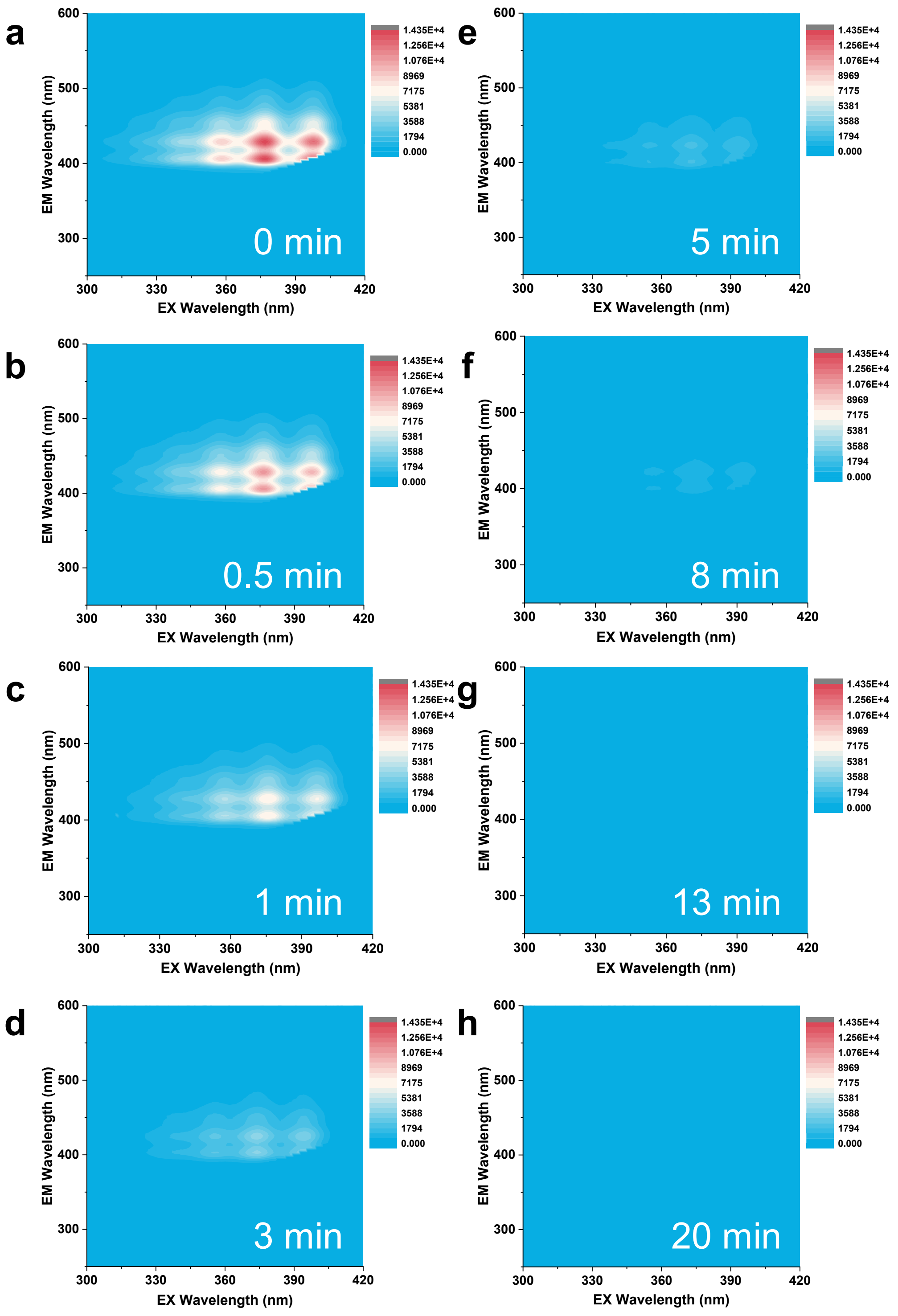

Supplementary Fig. 25 | EEM spectra of DMA in 50.0 wt.% acetonitrile in H_2O upon treatment with NFC/M system. Reaction time: **(a)** 0 min; **(b)** 0.5 min; **(c)** 1 min; **(d)** 3 min; **(e)** 5 min; **(f)** 8 min; **(g)** 13 min; **(h)** 20 min.

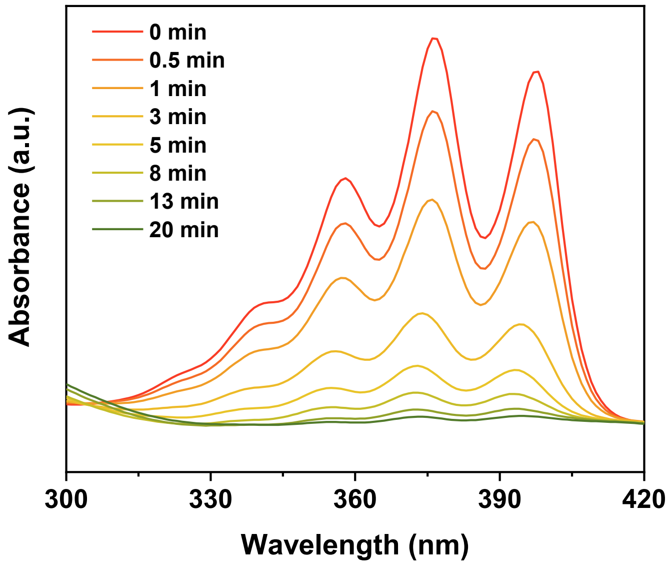

Supplementary Fig. 26 | UV-vis absorption spectra of DMA in 50.0 wt.% acetonitrile in H_2O upon treatment with NFC/M system.

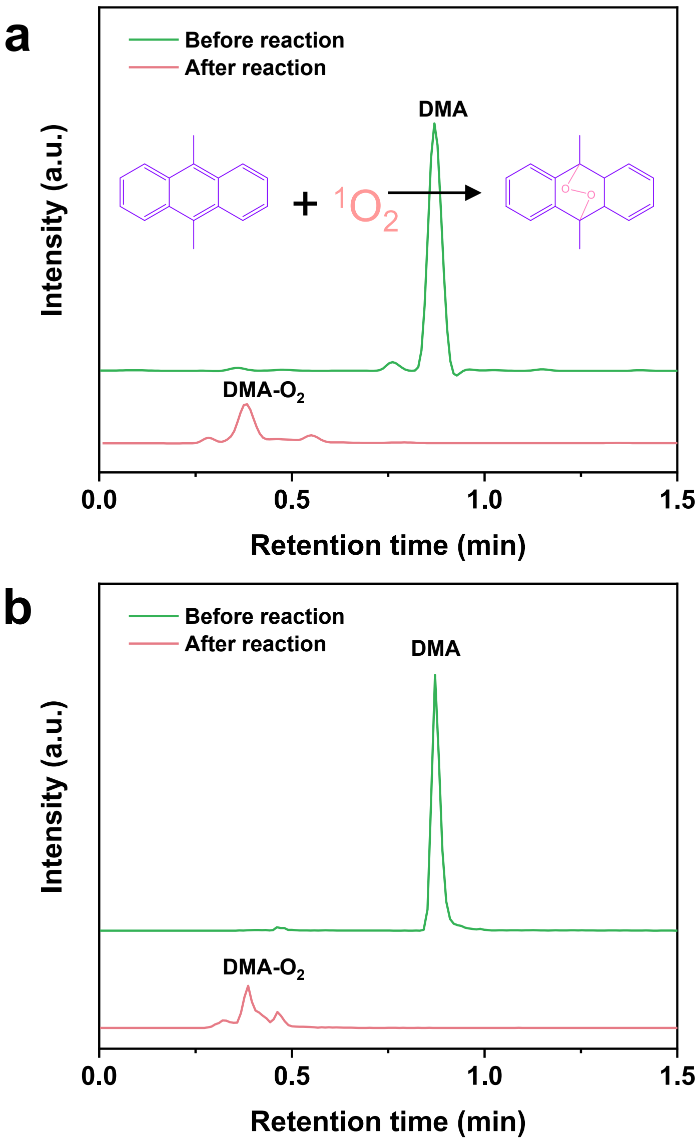

Supplementary Fig. 27 | (a) UPLC-MS chromatograms of the DMA reaction solution. **(b)** UPLC-MS extracted ion chromatograms of DMA reaction solution (DMA: 206.10634-206.11046, DMA-O_2: 240.10437-240.11433).

Notes for Supplementary Fig. 27

Further compelling evidence of robust $^1O_2$ generation was provided by the analysis of specific reaction products formed between classical chemical probe 9,10-dimethylanthracene (DMA) and $^1O_2$, which yielded the corresponding endoperoxide (DMA-O_2). The rapid diminution of DMA fluorescence in the presence of NFC/M was an indicative of substantial $^1O_2$ production (Fig. 4b and Supplementary Figs. 25 and 26). This was corroborated by the high-resolution mass spectrometry (HR-MS) results, in which a distinctive peak at 240.10954 Da was identified, corresponding to the mass of DMA-O_2 (Supplementary Fig. 27).

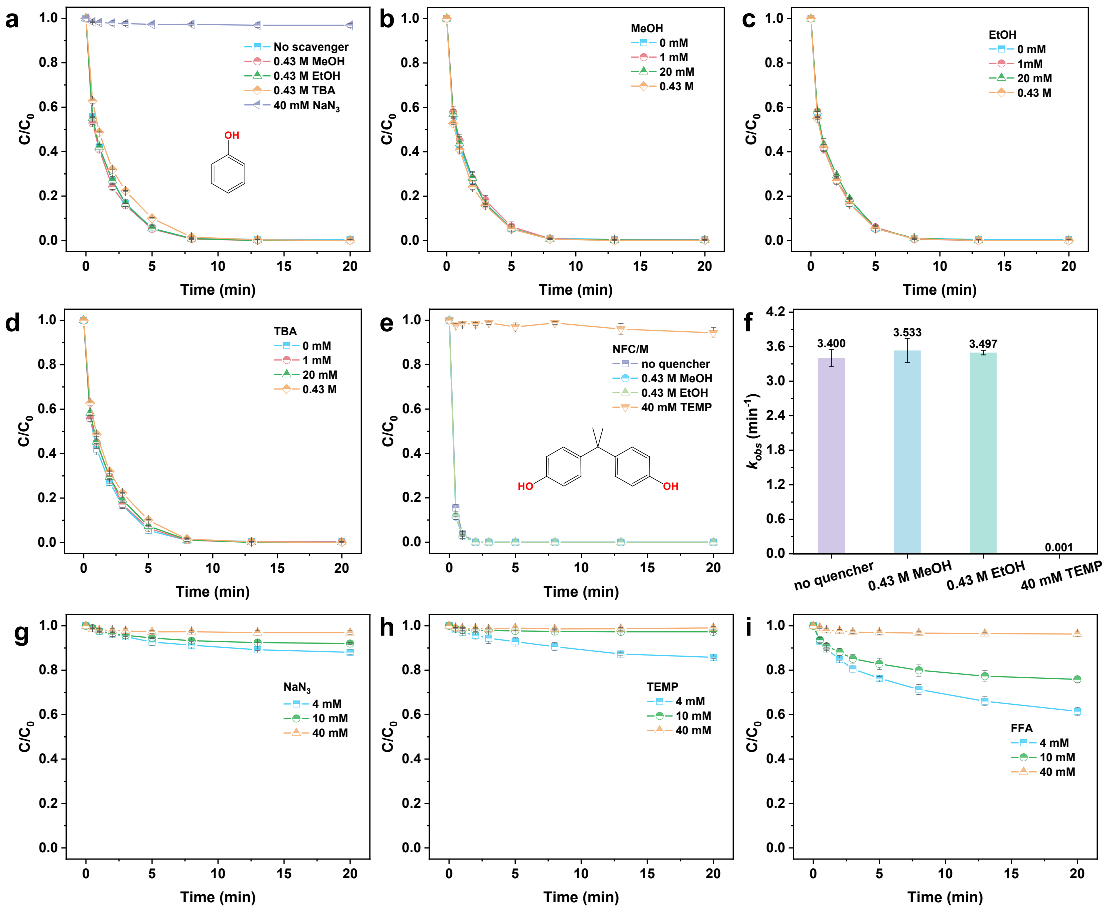

Supplementary Fig. 28 | Effects of ROS scavengers on phenol and BPA degradation in NFC/M system. **(a)** Quenching effects of different scavengers on phenol degradation. **(b-d)** The effects of the concentrations of radical-quenching agents (MeOH, EtOH, and TBA) on phenol degradation activity. **(e, f)** Quenching effects of different scavengers on BPA degradation kinetics. **(g-i)** The effects of the concentrations of $^1O_2$-quenching agents (NaN_3, FFA, and TEMP) on phenol degradation activity. Reaction conditions: [catalyst] = 0.2 g·L^{-1}, [PMS] = 0.65 mM, [pollutant] = 20.0 mg·L^{-1}, initial pH 7.0, Temp. = 20.0 ± 2.0 °C. Data are presented as mean values ± SD (n = 3).

Notes for Supplementary Fig. 28

Dosing MeOH and EtOH (scavengers for $SO_4^{\bullet-}$ and $^{\bullet}OH$) or TBA (scavenger for $^{\bullet}OH$) (1.0 mM to 0.43 M) exhibited minimal inhibitory effects on phenol degradation. This result indicates the absence of $^{\bullet}OH$ and $SO_4^{\bullet-}$ as active species. The very slight inhibition of excessive TBA might primarily be attributed to the increased solution viscosity, which impedes mass transfer.

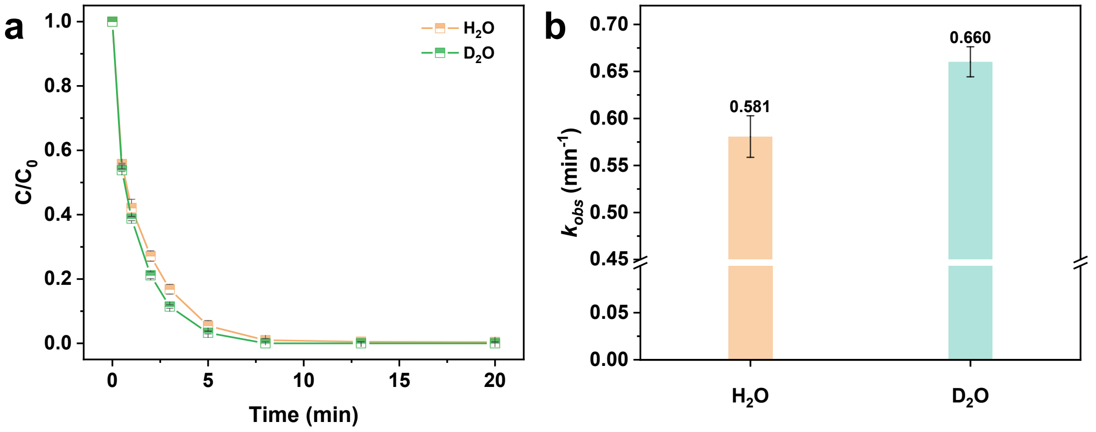

Supplementary Fig. 29 | Comparison of **(a)** degradation efficiency and **(b)** corresponding kinetic rate constant of phenol in H_2O and D_2O. Reaction conditions: [catalyst] = 0.2 g·L^{-1}, [PMS] = 0.65 mM, [phenol] = 20.0 mg·L^{-1}, Temp. = 20.0 ± 2.0 °C. Data are presented as mean values ± SD (n = 3).

Notes for Supplementary Fig. 29

In Fenton-like catalysis, deuterium oxide (D_2O) was used to prolong the lifetime of $^1O_2$ by reducing non-radiative deactivation processes that are more pronounced in H_2O. High-frequency O-H vibrations in H_2O deactivated $^1O_2$ more effectively than the O-D vibrations in D_2O, leading to a significantly extended lifetime of $^1O_2$ in D_2O (~67.0 µs) compared to H_2O (~3.5 µs). Such a higher vibrational threshold slows the rate of $^1O_2$ deactivation, which could theoretically enhance the longevity of reactive oxygen species and improve pollutant degradation.

However, D_2O could also affect catalyst reactivity and oxidant activation, sometimes leading to contradictory effects. While extending the lifetime of $^1O_2$, D_2O might simultaneously diminish catalyst reactivity by altering solvent dynamics and interaction energies. This modification can influence the overall activation of PMS and subsequent ROS generation. The modest increase in degradation rates observed in our experiments reflected the dual roles of D_2O in affecting both ROS longevity and catalytic activity.

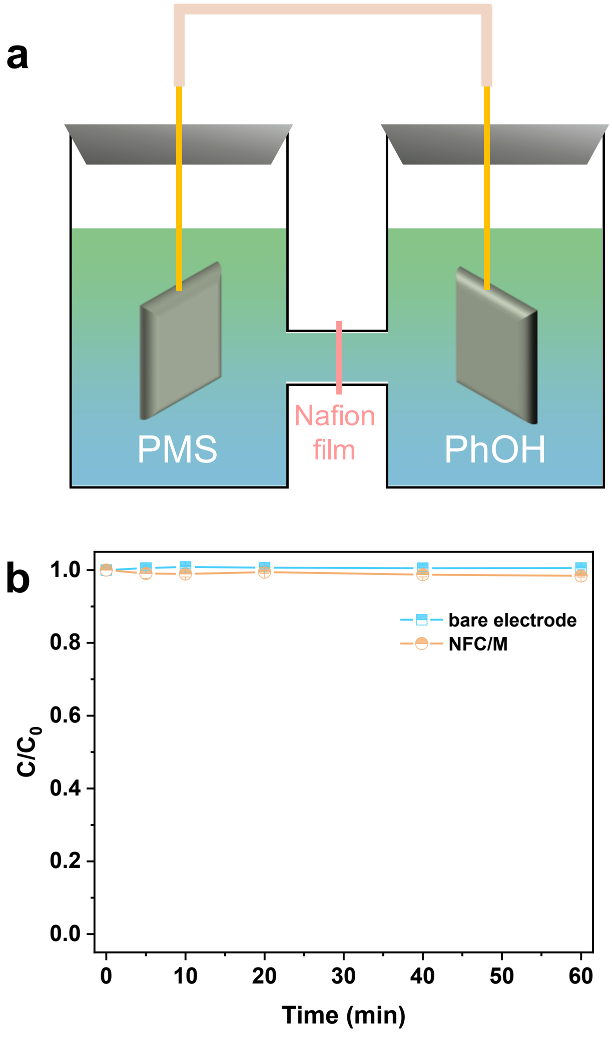

Supplementary Fig. 30 | (a) GOS reaction device setup. **(b)** Phenol degradation efficiency in the GOS reaction device. Data are presented as mean values ± SD (n = 3).

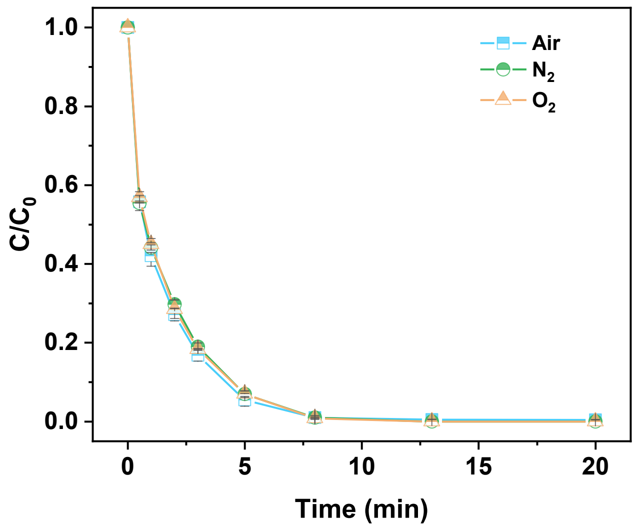

Supplementary Fig. 31 | Degradation of phenol under different atmospheres. Reaction conditions: [catalyst] = 0.2 g·L^{-1}, [PMS] = 0.65 mM, [phenol] = 20.0 mg·L^{-1}, Temp. = 20.0 ± 2.0 °C. Data are presented as mean values ± SD (n = 3).

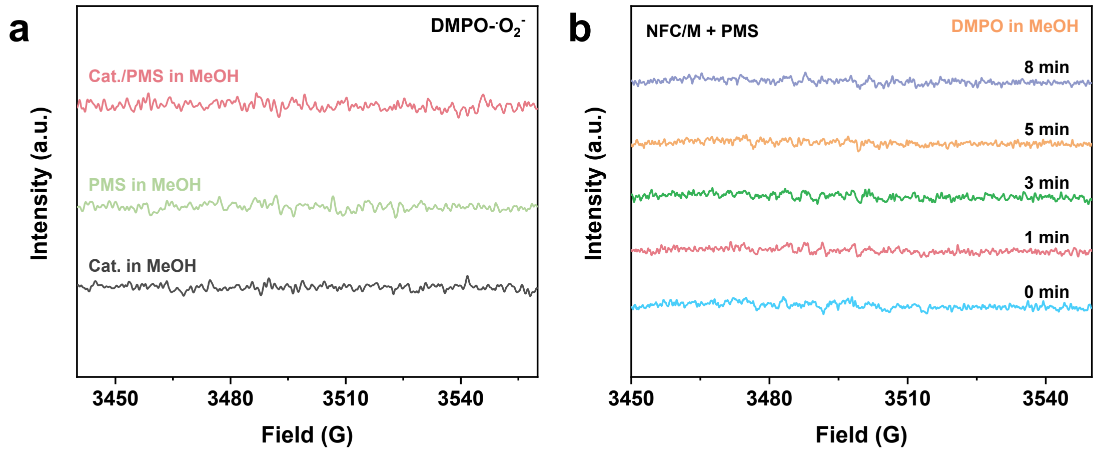

Supplementary Fig. 32 | Spin-trapping EPR spectra of DMPO-$^{\bullet}O_2^-$ in NFC/M system. **(a)** EPR spectra in different systems using DMPO as trapping agents. **(b)** EPR spectra in NFC/M-PMS system using DMPO as trapping agents.

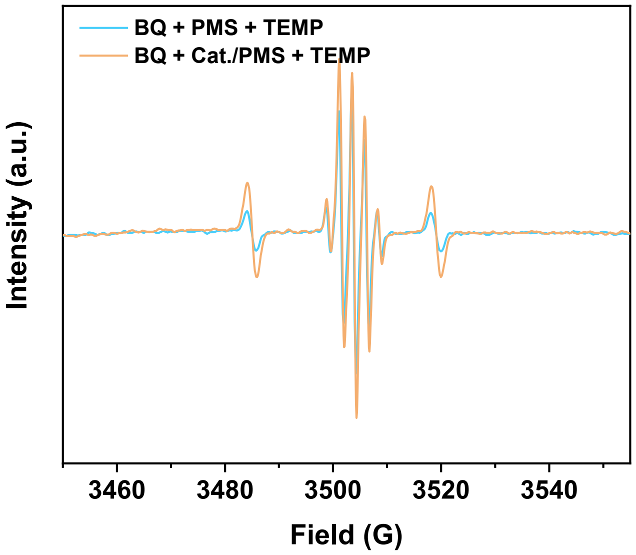

Supplementary Fig. 33 | EPR spectra for the detection of $^1O_2$ in the presence of TEMP and BQ. Reaction conditions: [catalyst] = 0.2 g·L^{-1}, [PMS] = 0.65 mM, [phenol] = 20.0 mg·L^{-1}, [BQ] = 0.4 mM, Temp. = 20.0 ± 2.0 ℃.

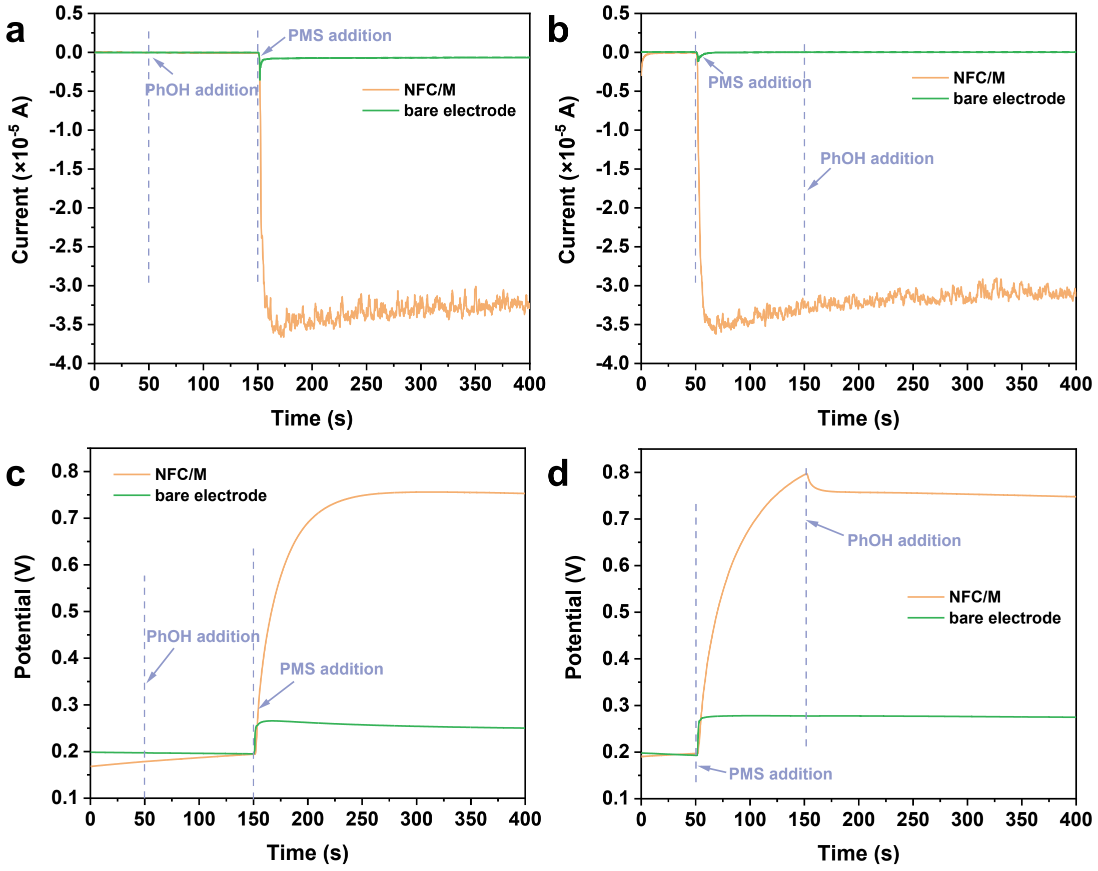

Supplementary Fig. 34 | (a, b) i-t curves, and **(c, d)** the time-profile of OCP upon addition of phenol and PMS, reaction condition: [PMS] = 0.65 mM, [phenol] = 20.0 mg·L^{-1}, initial pH = 7.0, Temp. = 20.0 ± 2.0 °C. No change in current was observed after the injection of phenol, indicating the absence of electron interaction between phenol and PMS or catalyst. The minor decrease in OCP can be attributed to the adsorption and enrichment of phenol on the NFC/M surface, potentially blocking active sites or changing the surface properties.

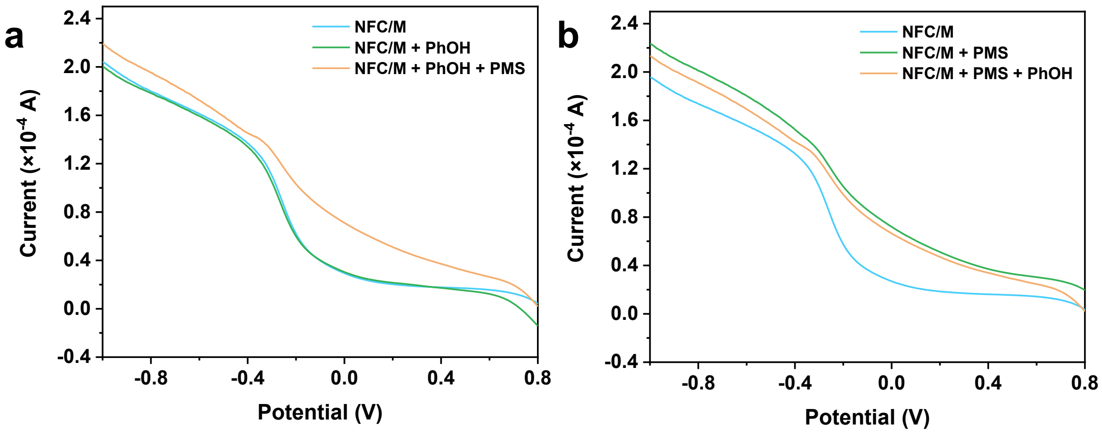

Supplementary Fig. 35 | LSV curves under different testing conditions. **(a)** Adding phenol first and then PMS. **(b)** Adding PMS first and then phenol.

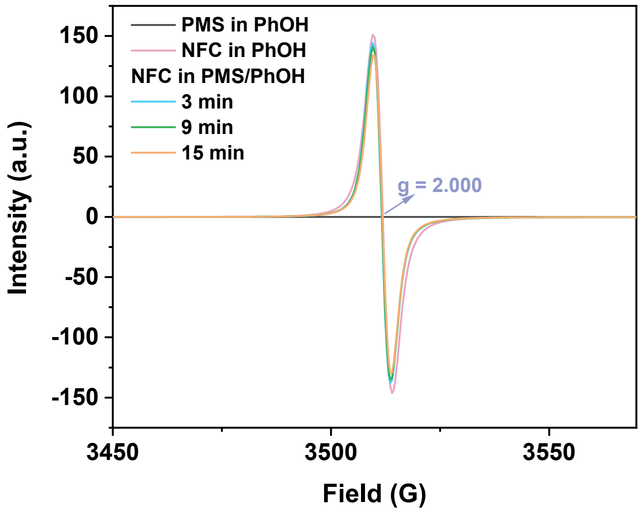

Supplementary Fig. 36 | In-situ EPR spectra of NFC reaction systems.

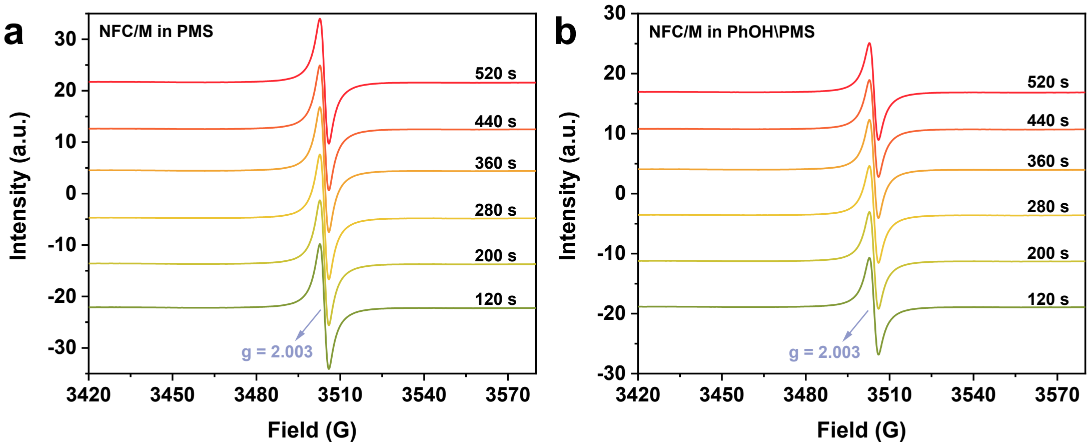

Supplementary Fig. 37 | In-situ EPR spectra of NFC/M-PMS reaction systems. **(a)** Without phenol. **(b)** With phenol.

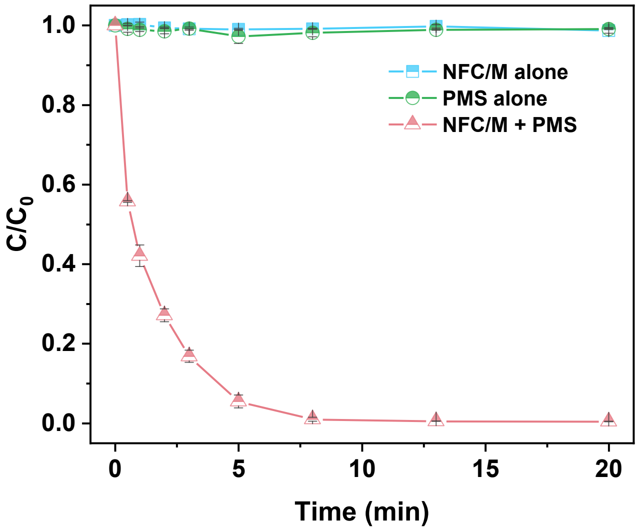

Supplementary Fig. 38 | Phenol adsorption and oxidation via PMS activation on NFC/M. Reaction conditions: [catalyst] = 0.2 g·L^{-1}, [PMS] = 0.65 mM, [phenol] = 20.0 mg·L^{-1}, initial pH 7.0, Temp. = 20.0 ± 2.0 °C. Data are presented as mean values ± SD (n = 3).

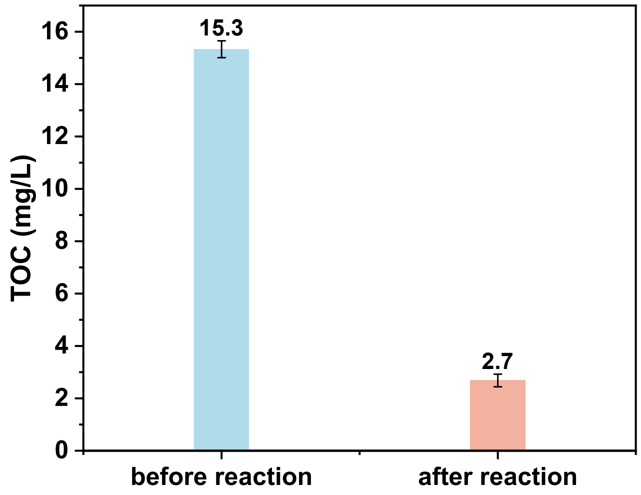

Supplementary Fig. 39 | TOC removal efficiency of NFC/M system for phenol degradation. Reaction conditions: [catalyst] = 0.2 g·L^{-1}, [PMS] = 0.65 mM, [phenol] = 20.0 mg·L^{-1}, initial pH 7.0, Temp. = 20.0 ± 2.0 °C, reaction time = 20 min. Data are presented as mean values ± SD (n = 3).

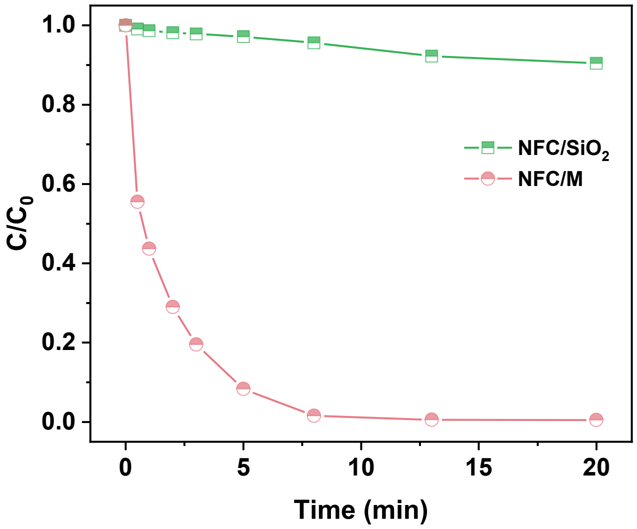

Supplementary Fig. 40 | Comparison in catalytic activity of NFC/M and NFC/SiO_2 for phenol degradation. Reaction conditions: [catalyst] = 0.2 g·L^{-1}, [PMS] = 0.65 mM, [phenol] = 20.0 mg·L^{-1}, initial pH 7.0, Temp. = 20.0 ± 2.0 °C.

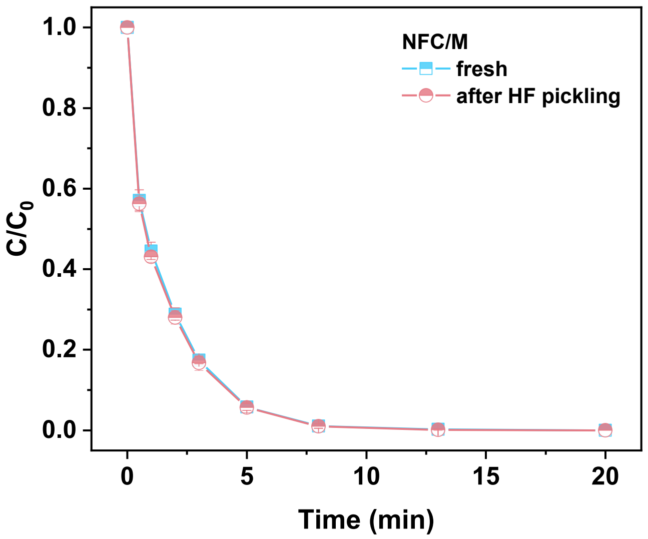

Supplementary Fig. 41 | Comparison of pollutant removal by NFC/M before and after HF etching for Si/Al/Na corrosion. Testing conditions: [catalyst] = 0.2 g·L^{-1}, [PMS] = 0.65 mM, [pollutant] = 20.0 mg·L^{-1}, initial pH 7.0, Temp. = 20.0 ± 2.0 °C. Data are presented as mean values ± SD (n = 3).

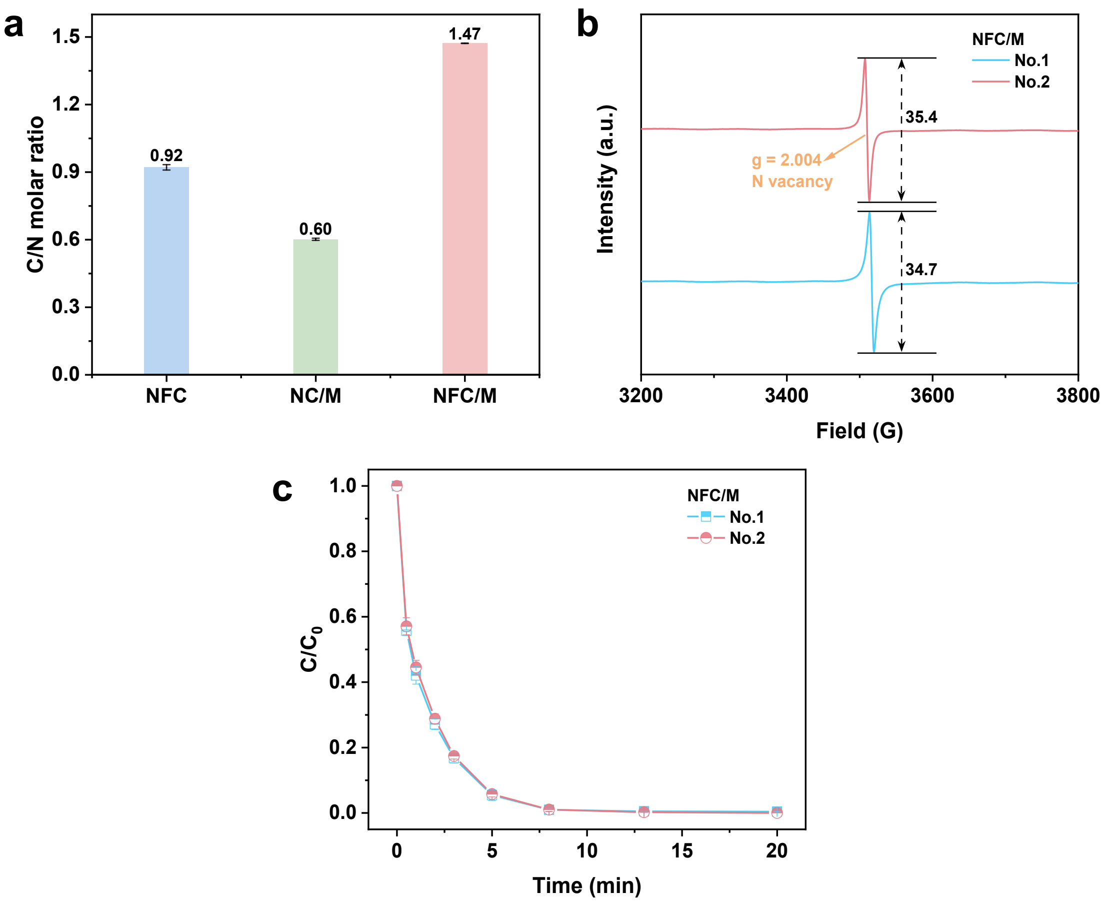

Supplementary Fig. 42 | Repeatability of NFC/M materials. **(a)** C/N molar ratio based on elemental analysis results. Error bars originate from statistical variations among materials synthesized in different batches. **(b)** Consistency of nitrogen vacancies evidenced by EPR spectra. **(c)** Performance consistency of parallel NFC/M samples in Fenton-like catalysis for pollutant degradation under controlled conditions. Conditions: [catalyst] = 0.2 g·L^{-1}, [PMS] = 0.65 mM, [pollutant] = 20.0 mg·L^{-1}, initial pH 7.0, Temp. = 20.0 ± 2.0 °C. Data are presented as mean values ± SD (n = 3).

Notes for Supplementary Fig. 42

These parallel experiments demonstrate good repeatability in the thermal synthesis of NFC/M, with minimal deviations observed in elemental composition (C/N ratio), nitrogen vacancy concentration (as indicated by similar EPR signal intensities), and performance in Fenton-like catalysis (phenol degradation) across different batches (Supplementary Fig. 42). This result indicates that our synthesis method was robust and repeatable.

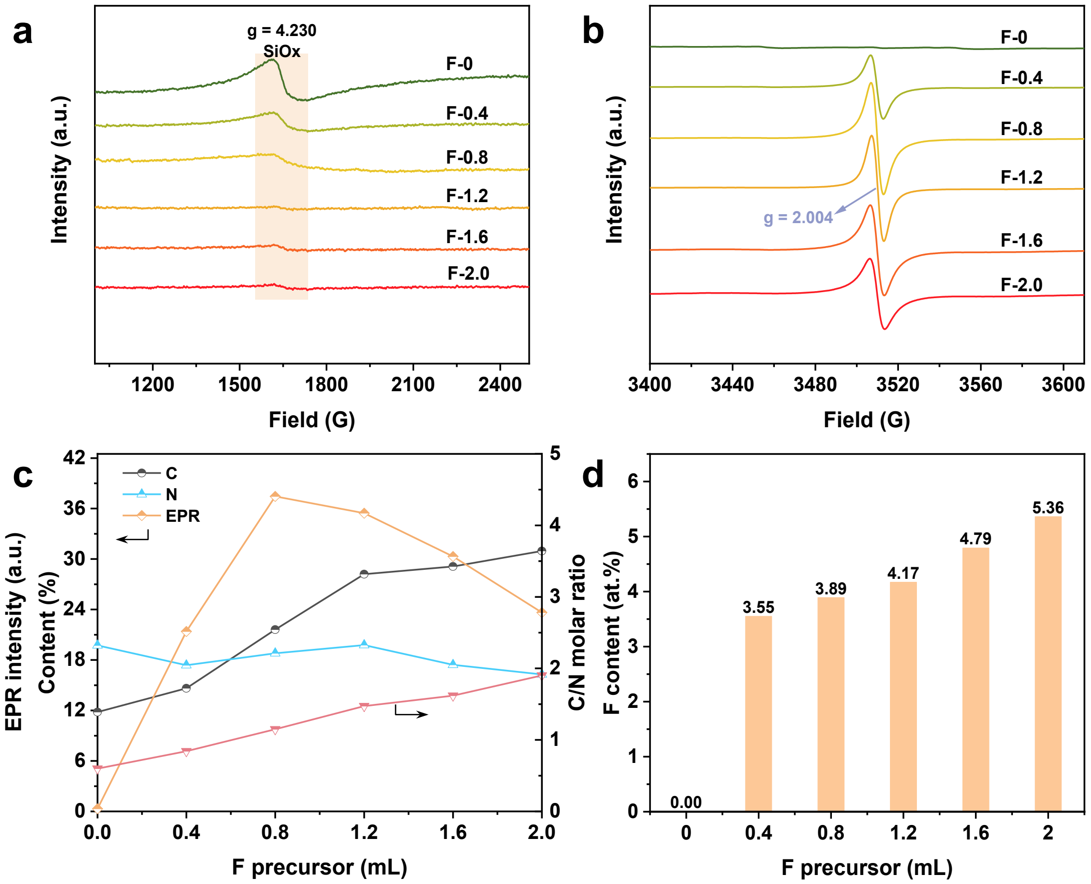

Supplementary Fig. 43 | The regulation of NFC/M materials prepared with various fluorine precursors. **(a)** Residual SiOx in different NFC/M samples, as indicated by EPR results. **(b)** Variation in nitrogen vacancies across NFC/M samples, evidenced by EPR spectra. **(c)** Comparative analysis of carbon, nitrogen, and nitrogen vacancies in the different NFC/M samples. **(d)** Fluorine content variation in NFC/M samples.

Notes for Supplementary Fig. 43

EPR results demonstrate that, as the dosage of PTFE increased, the degree of MMT template removal intensified and the concentration of NVs initially increased, but then declined (Supplementary Fig. 43a, b). Additionally, EA and XPS indicate that, with higher PTFE dosages, there was a corresponding increase in carbon and fluorine content in the materials, while the nitrogen content exhibited a slight but stable decrease (Supplementary Fig. 43c, d).

These findings suggest that varying the fluorine precursor dosage allowed partial control over the carbon/nitrogen ratio and the concentration of nitrogen vacancies in NFC/M. The capability to modulate these variables was crucial for optimizing the catalyst's performance in Fenton-like catalysis by tailoring the co-pyrolyzed fluorine precursor's contribution to the synthesis process.

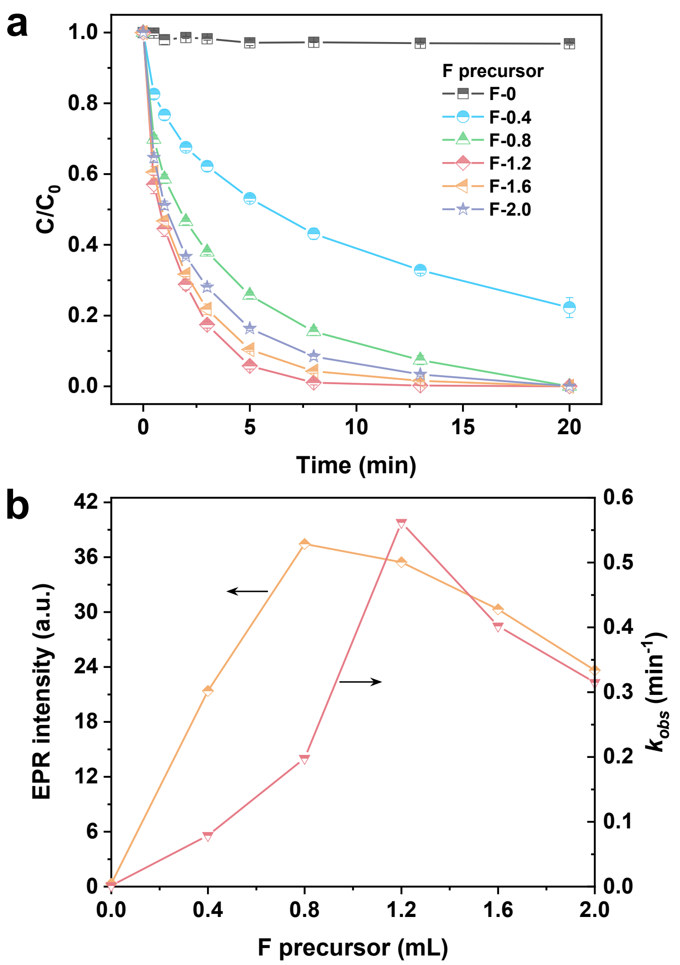

Supplementary Fig. 44 | The regulation of NFC/M materials prepared with various fluorine precursors for Fenton-like catalysis. **(a)** Comparison of pollutant removal by different NFC/M samples through PMS activation. **(b)** Correlation of nitrogen vacancies with Fenton-like reactivity in various NFC/M samples, analyzed through EPR spectra and pollutant degradation tests. Testing conditions: [catalyst] = 0.2 g·L^{-1}, [PMS] = 0.65 mM, [pollutant] = 20.0 mg·L^{-1}, initial pH 7.0, Temp. = 20.0 ± 2.0 ℃. Data for (**a**) are presented as mean values ± SD (n = 3).

Notes for Supplementary Fig. 44

The Fenton-like reactivity of NFC/M for pollutant degradation closely followed the concentration changes of defective nitrogen vacancies resulting from varying PTFE dosages during thermal preparation. While there was a greatly positive correlation, it was not strictly linear, mainly because both the PMS activation and pollutant degradation processes were influenced by the presence of F-C Lewis acid sites. These sites contributed to the regulated atomic and electronic structures and enhanced surface interactions, complicating the direct correlation between nitrogen vacancy concentration and catalytic performance.

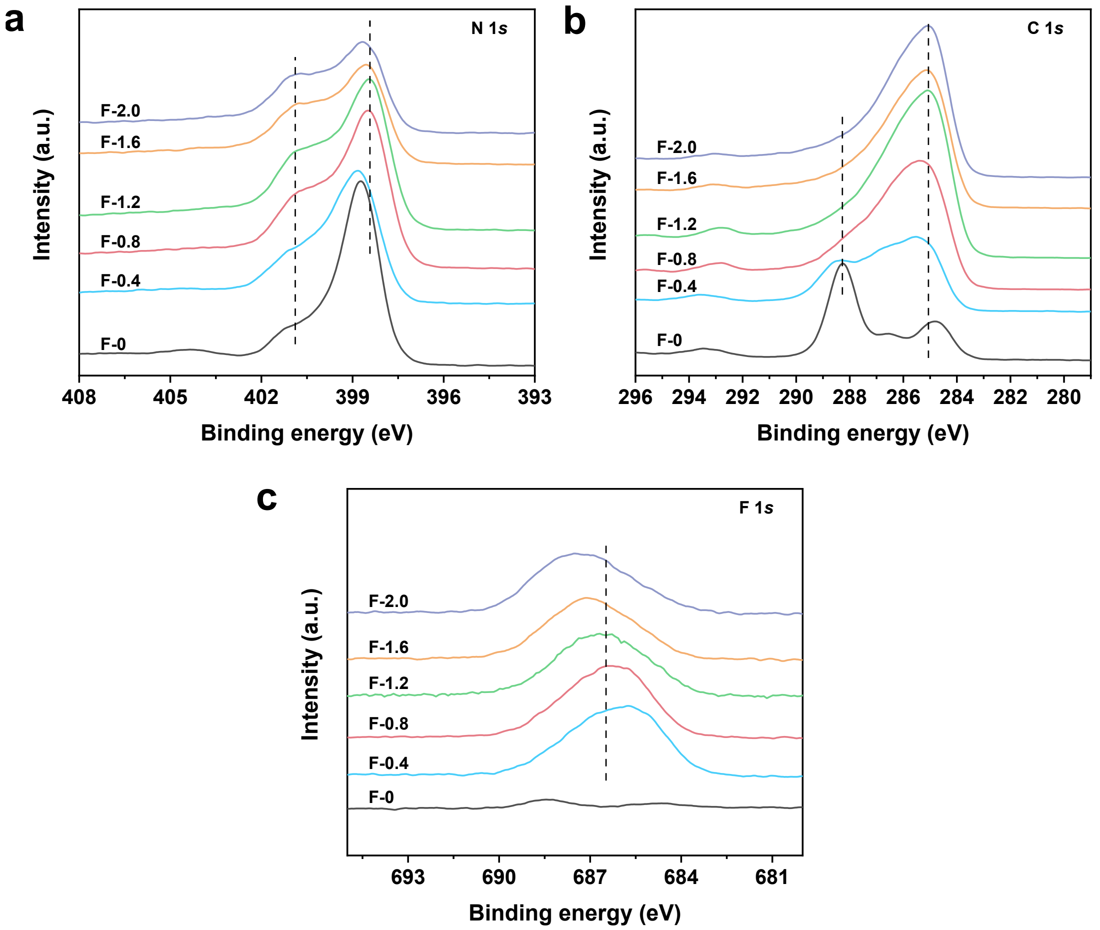

Supplementary Fig. 45 | The regulation of electronic structure of NFC/M materials prepared with various fluorine precursors. **(a)** Comparison in the binding energy of nitrogen in different NFC/M samples based on XPS spectra. **(b)** Comparison in the binding energy of carbon in different NFC/M samples based on XPS spectra. **(c)** Comparison in the binding energy of fluorine in different NFC/M samples based on XPS spectra. The dashed line indicates the binding energy position of the base sample NFC/M (F-1.2).

Notes for Supplementary Fig. 45

XPS analysis shows significant shifts in the binding energies of C, N, and F, which corresponded to the variations in NV and F concentrations (Supplementary Figs. 43, 45). These changes suggest dynamic adjustments in the catalyst's surface electronic structure, affecting both PMS activation and pollutant degradation (Supplementary Fig. 44).

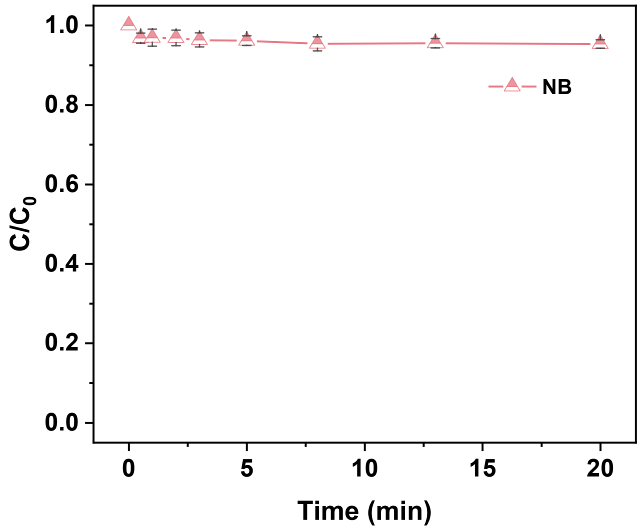

Supplementary Fig. 46 | Degradation performance of NFC/M-PMS system toward NB. Reaction conditions: [catalyst] = 0.2 g·L^{-1}, [PMS] = 0.65 mM, [NB] = 20.0 mg·L^{-1}, Temp. = 20.0 ± 2.0 ℃. Data are presented as mean values ± SD (n = 3).

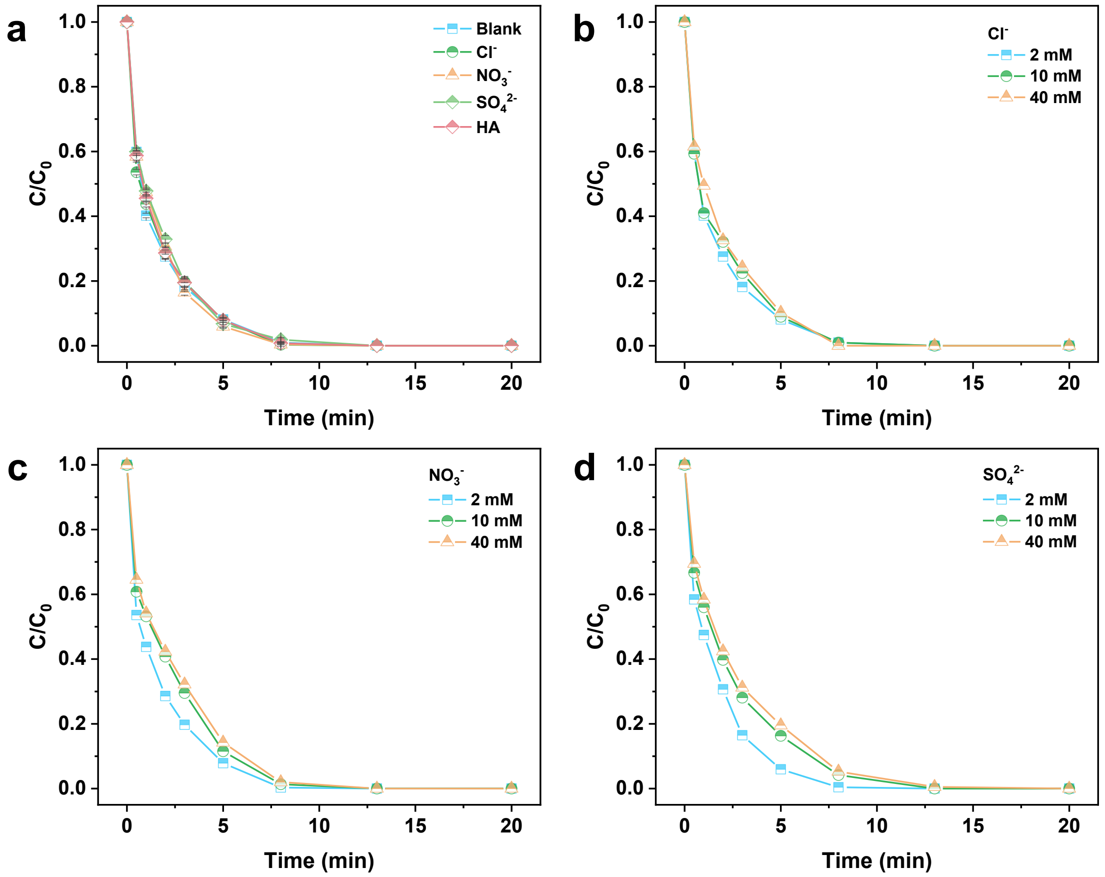

Supplementary Fig. 47 | (**a**) Phenol degradation by NFC/M-PMS system with interference of different ions and humic acids (HA). Data are presented as mean values ± SD (n = 3). (**b**) Effect of Cl^- concentration on phenol degradation. (**c**) Effect of NO_3^- concentration on phenol degradation. (**d**) Effect of SO_4^{2-} concentration on phenol degradation. Reaction conditions: [catalyst] = 0.2 g·L^{-1}, [PMS] = 0.65 mM, [phenol] = 20.0 mg·L^{-1}; ions = 2.0 mM and humic acid = 5.0 mg·L^{-1} (for **a**), initial pH 7.0, Temp. = 20.0 ± 2.0 °C.

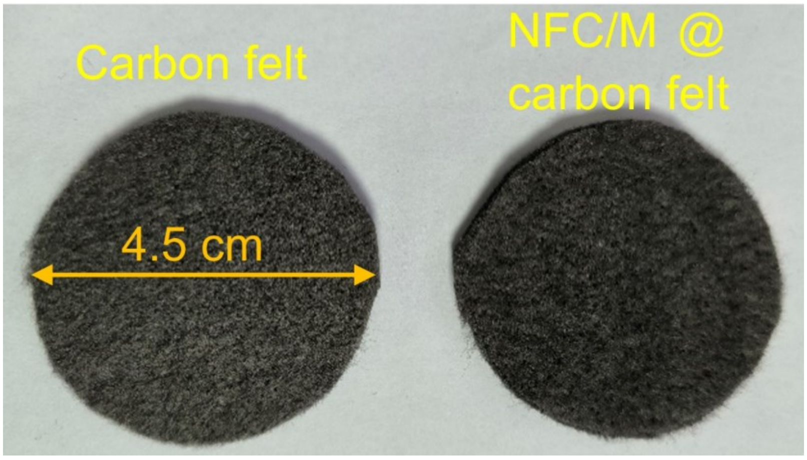

Supplementary Fig. 48 | Photographs of carbon felt and NFC/M @ carbon felt (the NFC/M catalyst coated on the carbon felt).

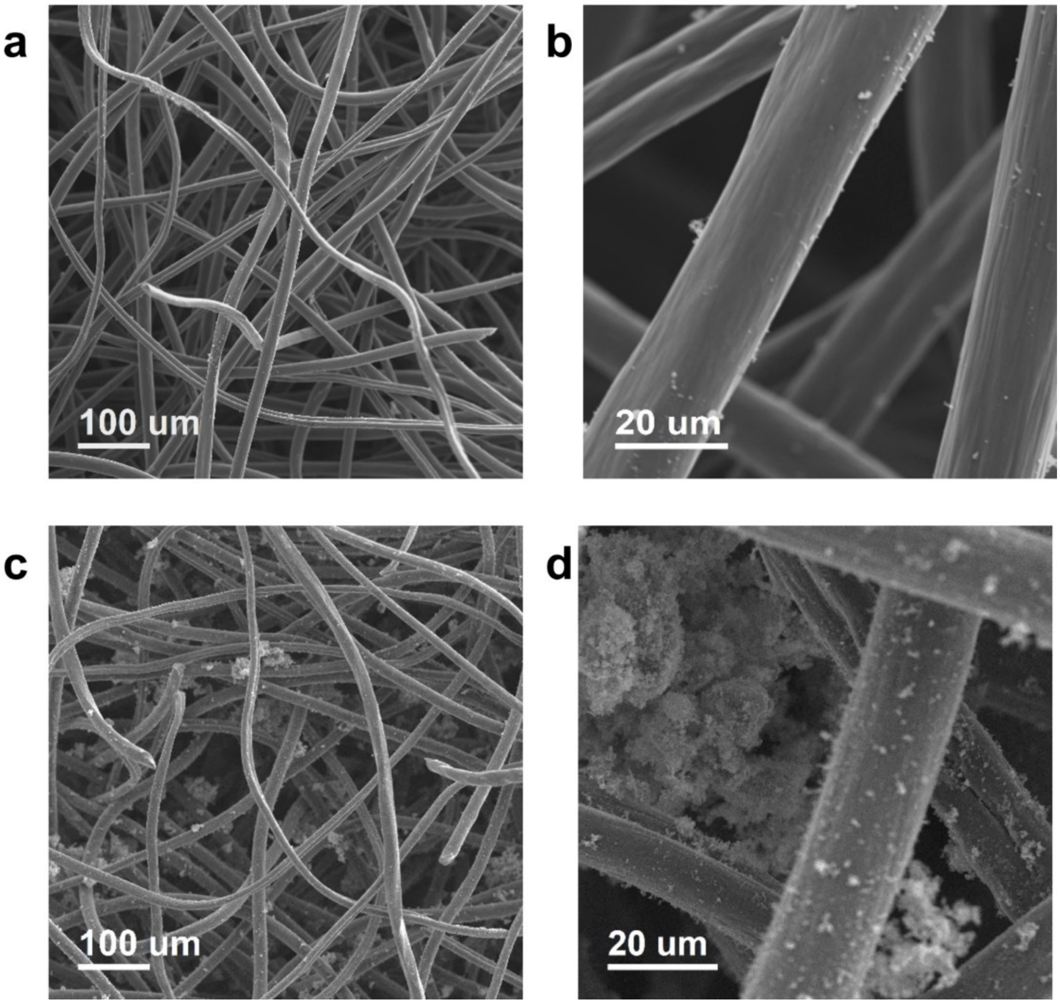

Supplementary Fig. 49 | SEM images of carbon felt **(a, b)** and NFC/M @ carbon felt **(c, d)**. The iamges show that NFC/M was loaded on the surface of the carbon felt and its crevices, which provided sufficient active catalytic sites for rapid water purification.

Supplementary Table 1 | Theoretical bond length of O-H ($l_{O\text{-}H}$) and O-O ($l_{O\text{-}O}$) of PMS on different models with diverse N vacancy active sites.

**Configuration**	$\boldsymbol{l_{O\text{-}H}}$ **(Å)**	$\boldsymbol{l_{O\text{-}O}}$ **(Å)**
free PMS	0.9767	1.509
NC-1	0.9916	1.479
NC-2	0.9970	1.476
NC-3	0.9972	1.465
NFC-1	0.9939	1.480
NFC-2	0.9977	1.469
NFC-3	0.9902	1.477

Supplementary Table 2 | Summary of N_2 sorption for NFC/M and NC/M.

**Catalyst**	**BET Surface Area ($m^2 \cdot g^{-1}$)**	**Pore Volume ($cm^3 \cdot g^{-1}$)**
NFC/M	69.7223	0.26538
NC/M	14.6566	0.05895
NFC	29.6106	0.124548

Supplementary Table 3 | C, N, F, Si, Al, O and Na contents of the synthesized catalysts determined by XPS analysis.

**Samples**		**C 1*s***	**N 1*s***	**F 1*s***	**Si 2*p***	**Al 2*p***	**O 1*s***	**Na 1*s***
NFC/M	BE (eV)	284.8	398.48	686.68	103.18	74.48	532.25	1071.44
	at%	63.45	24.14	4.17	0.34	1.16	5.50	1.24
	wt%	58.03	25.01	6.05	0.62	2.31	6.05	1.93
NC/M	BE (eV)	288.09	398.59	---	102.91	74.74	532.25	1071.63
	at%	25.65	18.10	---	12.01	3.75	39.4	1.09
	wt%	18.41	15.34	---	20.06	6.16	38.22	1.81
FC/M	BE (eV)	284.8	---	687.61	103.79	77.72	532.25	1073.52
	at%	65.08	---	23.37	0.38	1.99	8.07	1.11
	wt%	59.62	---	26.86	0.69	3.86	7.23	1.74
NFC/M-HF	BE (eV)	284.8	398.48	686.68	103.18	77.68	532.25	1071.44
	at%	61.58	26.57	4.87	0.06	1.38	5.49	0.05
	wt%	56.83	26.73	6.97	0.11	2.75	6.43	0.08

Supplementary Table 4 | Comparison of catalytic performance for organic contaminant degradation in recently reported PMS activation processes. The modified kinetic rate constant (*k*) was calculated through dividing the observed rate constant of contaminants by catalyst dosage and PMS concentration, followed by multiplying contaminant concentration.

Catalyst ($g \cdot L^{-1}$)	PMS (mM)	Pollutant ($mg \cdot L^{-1}$)	Removal efficiency	k_{obs} (min^{-1})	*k*-value ($min^{-1} \cdot M^{-1}$) #	Mechanism	Ref.
NFC/M (0.2)	0.65	BPA (20)	100% (2 min)	3.400	523.08	$^1O_2$	**This work**
NFC/M (0.2)	0.65	Phenol (20)	100% (8 min)	0.581	89.39	$^1O_2$	**This work**
NFC/M (0.2)	0.65	4-CP (25.7)	100% (8 min)	0.854	168.88	$^1O_2$	**This work**
STLC (1)	1.3	Phenol (20)	---	0.19	2.92	$SO_4^{\bullet-}$, $^1O_2$	*Nat. Commun.* **2023**, 14:3538
STLC (1)	1.3	o-nitrophenol (20)	95% (8 min)	0.344	5.29	$SO_4^{\bullet-}$, $^1O_2$	*Nat. Commun.* **2023**, 14:3538
SAMCC0.5 (0.3)	0.65	Phenol (20)	99% (15 min)	0.337	34.56	Co(IV)=O, $^1O_2$	*Proc. Natl. Acad. Sci. USA* **2023**, 120, e2305933120
BPY-COFs (0.3)	5.0	BPA (10)	100% (5 min)	1.25	8.33	$^1O_2$	*Angew. Chem. Int. Ed.* **2023**, e202310934
Cu-N4/C-B (0.1)	0.65	BPA (20)	98% (5 min)	0.56	172.31	Cu (III)-OH	*Proc. Natl. Acad. Sci. USA* **2022**, *119*, e2119492119
2.9% Cu1/NG (0.1)	0.5	BPA (20)	100% (5 min)	1.18	472	Electron-transfer	*Angew. Chem.* **2022**, *134*, e202207268
Cu-MnO_2 (0.3)	1.3	Phenol (35)	99% (40 min)	0.072	6.46	$^1O_2$	*J. Hazard. Mater.*, **2018**, *360*, 303-310.

$La_2CuO_{4-\delta}$ (0.1)	2.0	BPA (11.4)	60.1 (60 min)	0.014	0.798	Radicals, $^1O_2$	*Appl. Catal. B*. **2021**, *286*, 119910
SACu@NBC (0.1)	1.3	BPA (20)	100% (30 min)	0.28	43.08	Electron-transfer	*J. Mater. Chem. A*, **2021**, *9*, 11604.
CuFeMnO (0.2)	1.0	Phenol (28.2)	81% (30 min)	0.05	7.05	High-valent metal species, ·OH	*Proc. Natl. Acad. Sci. USA* **2022**, *119* (30), e2202682119
Co-SAs (0.2)	1.3	BPA (20)	80% (12 min)	0.157	12.08	$^1O_2$	*Small*, **2020**, *16*, 2005060
Co-TPML (0.2)	2.0	BPA (11.4)	100% (5 min)	2.4	68.4	Radicals	*Environ. Sci. Technol.* **2021**, *55*, 1242-1250
SACo-NGs (0.1)	1.0	BPA (12.8)	100% (5 min)	0.6	136.8	Electron-transfer	*Appl. Catal. B*. **2021**, *286*, 119912
Fe-SACs (0.2)	1.3	BPA (25)	88% (30 min)	0.104	10.0	$^1O_2$	*Angew. Chem. Int. Ed.* **2021**, *60*, 22513-22521
Fe1/CN (0.5)	1.0	BPA (22.8)	100% (10 min)	1.43	65.21	$^1O_2$	*Angew. Chem. Int. Ed.* **2021**, *60*, 4588-4593
Fe1/CN (0.5)	1.0	4-CP (12.85)	100% (10 min)	0.55	14.14	$^1O_2$	*Angew. Chem. Int. Ed.* **2021**, *60*, 4588-4593
Fe-g-C_3N_4 (0.10)	1.0	4-CP (13)	100% (20 min)	0.254	33.02	Fe (V)═O	*Environ. Sci. Technol.* **2018**, 52, 2197-2205.
$ZnFeMnO_4$ (0.1)	0.163	BPA (10)	100% (15 min)	0.43	263.8	Electron-transfer	*Proc. Natl. Acad. Sci. USA* **2022**, *119* (31), e2201607119
C-ZIF-67@ZIF-8@GO (0.02)	1.3	BPA (20)	75.4% (40 min)	0.026	20.0	$^1O_2$, ·OH	*J. Mater. Chem. A*, **2020**, 8, 3168.

Fe-Co-NC (0.1)	0.65	BPA (20)	100% (4 min)	1.252	385.23	$^1O_2$	*J. Am. Chem. Soc*. **2018**, *140*, 12469-12475
FeCo-N/C (0.05)	1.3	Phenol (15.3)	100% (20 min)	0.316	74.38	FeCo=O	*Appl. Catal. B*. **2022**, *309*, 121256
Fe-N-C (0.1)	0.4	BPA (11.4)	100% (10 min)	0.44	125.4	Radicals, $^1O_2$	*Chem. Eng. J*. **2022**, *427*, 130898

$$\#: k - \text{value} = \frac{k_{obs} \times c[Pollutant]}{\text{c[Catalyst]} \times \text{c[PMS]}}$$

Supplementary Table 5 | Water matrix characteristics of two real wastewater tested in this work.

Real wastewater	Source	Characteristics	
		COD ($mg \cdot L^{-1}$)	pH
Aniline production wastewater	Jintai Lihua Chemical Industry Technology Co., China	8000-12000	8.5-9.5
Biochemical wastewater	East China Engineering Science & Technology Co., China	200-300	7.0-9.0

The main components of the aniline production wastewater are aniline and its derivatives. The biochemical wastewater is the effluent from an industrial park wastewater treatment plant, in which the mixed wastewater in the pharmaceutical park is treated by a microelectrolysis preteatment process and activated sludge process in sequence.

Supplementary Table 6 | Performance of NFC/M-PMS system for treating actual wastewater.

Sample	COD before treatment ($mg \cdot L^{-1}$)	COD after treatment ($mg \cdot L^{-1}$)	Removal efficiency (%)
Aniline production wastewater	360.9	180.4	50.01
Biochemical Wastewater	265.0	137.2	48.23

Reaction conditions: [catalyst] = 0.5 $g \cdot L^{-1}$, [PMS] =3.9 mM, initial pH = 7.0, Temp. = 20.0 ± 2.0 ℃, reaction time: 60.0 min.

Supplementary References

1. Kresse, G. & Furthmiiller, J. Efficiency of ab-initio total energy calculations for metals and semiconductors using a plane-wave basis set. *Comp. Mater. Sci.* **6**, 15-50 (1996).
2. Kresse, G. & Furthmiiller, J. Efficient iterative schemes for ab initio total-energy calculations using a plane-wave basis set. *Phys. Rev. B* **54**, 11169 (1996).
3. Kresse, G. & Joubert, D. From ultrasoft pseudopotentials to the projector augmented-wave method. *Phys. Rev. B* **59**, 1758 (1999).
4. Grimme, S. et al. A consistent and accurate ab initio parametrization of density functional dispersion correction (DFT-D) for the 94 elements H-Pu. *J. Chem. Phys*. **132**, 154104 (2010).
5. Tang, W., Sanville, E. & Henkelman, G. A grid-based Bader analysis algorithm without lattice bias. *J. Phys. Condens. Matter.* **21**, 084204 (2009).
6. Wang, V. et al. VASPKIT: A user-friendly interface facilitating high-throughput computing and analysis using VASP code. *Comp. Phys. Commun.* **267**, 108033 (2021).
7. Xu, J. et al. Organic wastewater treatment by a single-atom catalyst and electrolytically produced H_2O_2. *Nat. Sustain.* **4**, 233-241 (2021).
8. Kang, Y. et al. Unveiling the spatially confined oxidation processes in reactive electrochemical membranes. *Nat. Commun.* **14**, 6590 (2023).
9. Cheng, K., Zhang, L. & McKay, G. Evaluating the microheterogeneous distribution of photochemically generated singlet oxygen using furfuryl amine. *Environ. Sci. Technol.* **57**, 7568-7577 (2023).
10. Lenzen, M., Kanemoto, K., Moran, D. & Geschke, A. Mapping the structure of the world economy. *Environ. Sci. Technol.* **46**, 8374-8381 (2012).
11. Liu, T. et al. Water decontamination via nonradical process by nanoconfined Fenton-like catalysts. *Nat. Commun.* **14**, 2881 (2023).
12. Zhang, S. et al. Mechanism of heterogeneous fenton reaction kinetics enhancement under nanoscale spatial confinement. *Environ. Sci. Technol.* **54**, 10868-10875 (2020).
13. Li, X. et al. Single cobalt atoms anchored on porous n-doped graphene with dual reaction sites for efficient fenton-like catalysis. *J. Am. Chem. Soc.* **140**, 12469-12475 (2018).
