# Supplementary material for: Tuning electronic structure of metal-free dual-site catalyst enables exclusive singlet oxygen production and in-situ utilization
Source: Nat Commun. 2024 Jul 10;15:5771. doi: 10.1038/s41467-024-50240-0 (PMC11535063; doi:10.1038/s41467-024-50240-0)
Supplement: Supplementary file 3 — Description of Additional Supplementary Files [file 41467_2024_50240_MOESM3_ESM.pdf]

Description of Additional Supplementary Files

File Name: Supplementary Data 1
Description: This file contains the atomic coordinates of the studied structures.
