# Supplementary material for: Tuning electronic structure of metal-free dual-site catalyst enables exclusive singlet oxygen production and in-situ utilization
Source: Nat Commun. 2024 Jul 10;15:5771. doi: 10.1038/s41467-024-50240-0 (PMC11535063; doi:10.1038/s41467-024-50240-0)
Supplement: Supplementary file 4 — Supplementary Data 1 [file 41467_2024_50240_MOESM4_ESM.pdf]

```
Nv-NC-1
    1.00000000000000
      14.7600002288999992       0.0000000000000000       0.0000000000000000
      -7.3800001143999996      12.7825351581000000       0.0000000000000000
       0.0000000000000000       0.0000000000000000      20.0000000000000000
    C     N
     68     3
Direct
  0.0003249280035421 -0.0003249280035421  0.5000000000000000
  0.1132604330221673  0.0587416022504763  0.5000000000000000
  0.1671428990596529  0.0022230597117969  0.5000000000000000
  0.2796686418966196  0.0590229924323073  0.5000000000000000
  0.3342646578238483  0.0031261957617596  0.5000000000000000
  0.4460045596298410  0.0577470646181716  0.5000000000000000
  0.5005667275549739  0.0010709552878842  0.5000000000000000
  0.6115281708483690  0.0536679800453752  0.5000000000000000
  0.6667782930480259 -0.0021965612305550  0.5000000000000000
  0.7786849079152321  0.0539889156220555  0.5000000000000000
  0.8328751094918228 -0.0019392230734834  0.5000000000000000
  0.9450852988746536  0.0549147081253436  0.5000000000000000
  0.0019392230734834  0.1671248615081784  0.5000000000000000
  0.1151924082363879  0.2255395657055758  0.5000000000000000
  0.1732106285045128  0.1738811693418005  0.5000000000000000
  0.2876084751164682  0.2327123858052602  0.5000000000000000
  0.3370802151567192  0.1715722854715921  0.5000000000000000
  0.4477656940948276  0.2254829373876271  0.5000000000000000
  0.5023119787210883  0.1690554559621880  0.5000000000000000
  0.6140076092154881  0.2214946993772961  0.5000000000000000
  0.6686134992407622  0.1658575887636032  0.5000000000000000
  0.7792633782333793  0.2207366217666204  0.5000000000000000
  0.8348451518521255  0.1651548191478758  0.5000000000000000
  0.9460110913779414  0.2213150920847678  0.5000000000000000
  0.0021965612305550  0.3332217359519726  0.5000000000000000
  0.1133327282715046  0.3891601860830061  0.5000000000000000
  0.2818203708079757  0.3923109698776128  0.5000000000000000
  0.3477376893863400  0.3482550910002132  0.5000000000000000
  0.4717598224502962  0.4116685004340567  0.5000000000000000
  0.5068369125071267  0.3369928326869447  0.5000000000000000
  0.6158636579858809  0.3841363120141241  0.5000000000000000
  0.7785053006227044  0.3859923607845163  0.5000000000000000
  0.8341423822363985  0.3313865297592364  0.5000000000000000
  0.9463320269546220  0.3884717991516355  0.5000000000000000
 -0.0010709552878842  0.4994332724450264  0.5000000000000000
  0.1095696646507952  0.5551553033020933  0.5000000000000000
```

0.1654520877280574	0.5002411066928202	0.5000000000000000
0.2763090327728022	0.5583096411633464	0.5000000000000000
0.3319469548827678	0.5035875439925167	0.5000000000000000
0.4386390525034291	0.5613609474965714	0.5000000000000000
0.5883314695659484	0.5282401775497040	0.5000000000000000
0.6630071963130542	0.4931630874928729	0.5000000000000000
0.7745170626123734	0.5522343059051732	0.5000000000000000
0.8309445150378133	0.4976880212789117	0.5000000000000000
0.9422529423818252	0.5539954403701591	0.5000000000000000
-0.0031261957617596	0.6657353711761503	0.5000000000000000
0.1094719777022716	0.7227460931303473	0.5000000000000000
0.1650190480553830	0.6676159578658860	0.5000000000000000
0.2758658380948124	0.7241341619051881	0.5000000000000000
0.3313771625587208	0.6686228664412778	0.5000000000000000
0.4416903588366539	0.7236909672271977	0.5000000000000000
0.4964124560074834	0.6680530741172315	0.5000000000000000
0.6076890001223920	0.7181796291920242	0.5000000000000000
0.6517449379997853	0.6522623396136586	0.5000000000000000
0.7672876141947398	0.7123915248835320	0.5000000000000000
0.8284276855284086	0.6629198138432792	0.5000000000000000
0.9409770145676897	0.7203313581033800	0.5000000000000000
-0.0022230597117969	0.8328570719403482	0.5000000000000000
0.1099358261770398	0.8900642038229550	0.5000000000000000
0.1656279221370674	0.8343720488629340	0.5000000000000000
0.2772539068696526	0.8905280522977231	0.5000000000000000
0.3323840711341129	0.8349809229446182	0.5000000000000000
0.4448446966979072	0.8904303653492004	0.5000000000000000
0.4997588933071797	0.8345478832719442	0.5000000000000000
0.6108397839169982	0.8866673017284905	0.5000000000000000
0.7744604342944239	0.8848076217636073	0.5000000000000000
0.8261188016582004	0.8267893424954883	0.5000000000000000
0.9412584047495206	0.8867395969778282	0.5000000000000000
0.1699323856654567	0.3353474842602672	0.5000000000000000
0.6698179148515563	0.3301821141484426	0.5000000000000000
0.6646525447397321	0.8300675853345442	0.5000000000000000

```
Nv-NC-2
   1.00000000000000
    14.7600002288999992    0.0000000000000000    0.0000000000000000
    -7.3800001143999996   12.7825351581000000    0.0000000000000000
     0.0000000000000000    0.0000000000000000   20.0000000000000000
   C    N
    66     3
Direct
 -0.0008364743391215 -0.0012214292014516  0.5000000000000000
  0.1105388866073847  0.0559167702502921  0.5000000000000000
  0.1694100415558577  0.0038093633274775  0.5000000000000000
  0.2822317834306781  0.0632106925683004  0.5000000000000000
  0.3363613837901325  0.0044498621846495  0.5000000000000000
  0.4464846577843227  0.0593484649745192  0.5000000000000000
  0.5010677442797097  0.0003362004912925  0.5000000000000000
  0.6110743804520817  0.0546607155812673  0.5000000000000000
  0.6651706964009828 -0.0045070139520502  0.5000000000000000
  0.7760184727898621  0.0528169407856284  0.5000000000000000
  0.8305862928819272 -0.0041925625856268  0.5000000000000000
  0.9414818520858613  0.0530302154047380  0.5000000000000000
 -0.0033909209117266  0.1645357193799656  0.5000000000000000
  0.1080428346711294  0.2212800279491974  0.5000000000000000
  0.1647537393144629  0.1672276319413466  0.5000000000000000
  0.2761778486673214  0.2280129413952746  0.5000000000000000
  0.4508895461712753  0.2337117000447539  0.5000000000000000
  0.5019179108662100  0.1717880928306722  0.5000000000000000
  0.6123225701103270  0.2279626395063356  0.5000000000000000
  0.6664544857305451  0.1683054115002197  0.5000000000000000
  0.7762665890751637  0.2226697486966561  0.5000000000000000
  0.8311134515439300  0.1647049342416052  0.5000000000000000
  0.9416533454652400  0.2207204259444900  0.5000000000000000
 -0.0019873762485938  0.3328352957180111  0.5000000000000000
  0.1083011064545685  0.3882483134587097  0.5000000000000000
  0.1637372056808810  0.3319665708619957  0.5000000000000000
  0.2744202909817836  0.3844377195472180  0.5000000000000000
  0.6076415594698305  0.3999623317405327  0.5000000000000000
  0.6668179541066689  0.3409350735912355  0.5000000000000000
  0.7772239715781296  0.3916243277976971  0.5000000000000000
  0.8317392843525275  0.3340440893968767  0.5000000000000000
  0.9429861106262849  0.3895053563817221  0.5000000000000000
 -0.0004344539575702  0.5002301008033689  0.5000000000000000
  0.1108417286240795  0.5555722180842144  0.5000000000000000
  0.1652339797724046  0.4990250912741013  0.5000000000000000
  0.2771300738429498  0.5537411199488865  0.5000000000000000
```

0.3346556019022375	0.4994593268638388	0.5000000000000000
0.6687683368328480	0.5199427646668059	0.5000000000000000
0.7777789648424113	0.5591604489800953	0.5000000000000000
0.8334899480832519	0.5023473417560668	0.5000000000000000
0.9445441027266788	0.5573088175856379	0.5000000000000000
0.0029960630079341	0.6680950736614254	0.5000000000000000
0.1138851962973429	0.7241458136219293	0.5000000000000000
0.1685296165899397	0.6672414084332252	0.5000000000000000
0.2791167168476130	0.7223265503827475	0.5000000000000000
0.3338933164064493	0.6645592903893113	0.5000000000000000
0.4446492912121018	0.7169802328798718	0.5000000000000000
0.4949563316083830	0.6541608089963566	0.5000000000000000
0.6090711075200642	0.7038274356702239	0.5000000000000000
0.6692943570230536	0.6472220313916397	0.5000000000000000
0.7781356391137308	0.7178650498498965	0.5000000000000000
0.8399688469088532	0.6693795780914839	0.5000000000000000
0.9483806946305270	0.7243746121660380	0.5000000000000000
0.0036099310843516	0.8360879378262938	0.5000000000000000
0.1143232868396028	0.8924643088021468	0.5000000000000000
0.1698496535305603	0.8356010270035201	0.5000000000000000
0.2803900038985327	0.8916482057739393	0.5000000000000000
0.3351505938878976	0.8340955145835942	0.5000000000000000
0.4460991839915022	0.8882798946228351	0.5000000000000000
0.5004428122284442	0.8295843052503631	0.5000000000000000
0.6102261563003663	0.8820977938128935	0.5000000000000000
0.6638553630699822	0.8203164533352548	0.5000000000000000
0.7749837682526339	0.8825208782707378	0.5000000000000000
0.8323408735411983	0.8294706068091211	0.5000000000000000
0.9446551262764485	0.8879304903550865	0.5000000000000000
0.3371498621046730	0.1748900120799776	0.5000000000000000
0.3266326839767738	0.3325706253096488	0.5000000000000000
0.5037539801805170	0.3403408129638373	0.5000000000000000
0.4393899365755460	0.5485896169500615	0.5000000000000000

Nv-NC-3
1.00000000000000
14.8037004471000007 0.0000000000000000 0.0000000000000000
-7.4018502235000003 12.8203806571999994 0.0000000000000000
0.0000000000000000 0.0000000000000000 20.0000000000000000
C N
62 3
Direct

0.1073462433046740	0.0653691313312859	0.4999981689786139
0.0501489662346293	0.1220566616173239	0.4999957221295574
0.2748271180527945	0.0596025590809858	0.4999988319950913
0.2191180748579387	0.1184167932404657	0.4999982707168851
0.4440492101392587	0.0557033740950822	0.4999999829296423
0.3878713496321882	0.1116996189234207	0.4999992814977974
0.6116516807964530	0.0470934343164344	0.4999998757466882
0.5566700130990454	0.1072452872313499	0.5000002358121615
0.7809214037104140	0.0435462960121924	0.4999994822275557
0.7240058504063003	0.1002494281317603	0.4999997181144785
0.9426168509917340	0.0552692654752532	0.4999948476190418
0.8885443413407472	0.1100822495786665	0.5000014869327798
0.0654452228360941	0.2324197495910184	0.4999978776306184
0.2793144507277276	0.2323931495096522	0.4999986501112506
0.2584602444539822	0.3183323558048907	0.4999983474994810
0.4461656283591697	0.2222079331711797	0.4999981697206422
0.3901442391438289	0.2776820941004319	0.4999979307758125
0.6143483893660140	0.2194462729616898	0.4999995347864242
0.5574755357943746	0.2776658858670824	0.4999974823111573
0.7843451142769609	0.2153280146951408	0.4999995817027723
0.7265255879556263	0.2728544487439626	0.4999988648726265
0.9561209144328378	0.2182572360335323	0.4999998541328175
0.8990932483563582	0.2750336652416536	0.4999998999637098
0.1605880173577091	0.4312939745596338	0.4999970797900194
0.0649024146419127	0.4469991859051066	0.4999962853872571
0.1655077229569626	0.3304141033852093	0.4999981215787708
0.4381654599286936	0.3858454476343172	0.4999982214260763
0.6092865995158624	0.3894790997043445	0.4999990246077615
0.5486483878536144	0.4492281193636676	0.4999995557010616
0.7798011975517465	0.3849563752367253	0.5000010042867162
0.7215514160287334	0.4415628115413051	0.4999983108817043
0.9527905578638379	0.3875734288998491	0.4999956718192372
0.8923618801556400	0.4422720671353961	0.4999974606637390
0.1099377783532309	0.5570468841871696	0.5000000094669910
0.0544588384401982	0.6123166616627669	0.4999976446776064
0.2206863091201274	0.6040413446918342	0.4999998624256214

0.6119038922152298	0.5625833270761154	0.5000004635289905
0.7773374330044495	0.5532992149841606	0.4999985656636983
0.7223763936536443	0.6087932634542736	0.5000006960512668
0.9440878635710447	0.5546536766703911	0.4999961285381651
0.8878486025980905	0.6110355950858246	0.4999968473370063
0.1102728707102708	0.7242286776440546	0.5000006048876237
0.0520140903849202	0.7806292936319762	0.4999989877393270
0.2228118355088295	0.7761737864050503	0.4999992444078523
0.3949999109692499	0.7796865692337621	0.5000003837328832
0.6682559207888784	0.8354950199587785	0.4999985315363827
0.7672057264261501	0.7188913226621171	0.5000003967625313
0.6727058853010189	0.7350616485424554	0.4999999233304741
0.9393911752427117	0.7233751962840965	0.4999981270078068
0.8789640046402666	0.7781294371071270	0.4999988698925594
0.1053236177384869	0.8931536974817303	0.4999992516002889
0.0475094171393420	0.9502868662119108	0.4999978510668394
0.2743391207871225	0.8886582548325385	0.4999993956178622
0.2175480379835575	0.9466546607455105	0.4999990196974413
0.4414957204696601	0.8902350228035325	0.5000009343467212
0.3861302530693234	0.9450844998240879	0.5000001286900317
0.5676657854237037	0.8404120059360208	0.5000004994802750
0.5518923914284609	0.9352299934017498	0.5000006598323752
0.7654316585075793	0.9323183699220916	0.4999962446977791
0.9323795834501251	0.8905410996549898	0.4999971654765606
0.8757097500233625	0.9473543338595029	0.4999944041123452
0.2822085365574932	0.7169495856799302	0.4999986494400885
0.2479556071272117	0.5264846598187057	0.4999969351901568
0.5847606150493561	0.6391348749764411	0.5000000118355660
0.4720880971930604	0.7527517094493082	0.5000006155789556

Nv-NFC-1

```
     1.00000000000000
       14.7600002288999992         0.0000000000000000         0.0000000000000000
       -7.3800001143999996        12.7825351581000000         0.0000000000000000
        0.0000000000000000         0.0000000000000000        20.0000000000000000
     C      F      N
      67      1      3
Direct
  -0.0016409346940423 -0.0008710388892864  0.5000000000000000
   0.1077424340009219  0.0533038120193322  0.5000000000000000
   0.1625710952054779 -0.0038396302776272  0.5000000000000000
   0.2687944595264913  0.0376161843921364  0.5000000000000000
   0.3335811006631852 -0.0038234578401232  0.5000000000000000
   0.4455659480497094  0.0533227770438634  0.5000000000000000
   0.5007454050184825 -0.0008686187463756  0.5000000000000000
   0.6125310356465535  0.0567796172191861  0.5000000000000000
   0.6679970440287347  0.0015833161236266  0.5000000000000000
   0.7788402887236959  0.0576855963034276  0.5000000000000000
   0.8335774482923098  0.0015838297112898  0.5000000000000000
   0.9442502538939418  0.0567641319975027  0.5000000000000000
  -0.0004958442068109  0.1686076863736771  0.5000000000000000
   0.1098788328572445  0.2249893403662014  0.5000000000000000
   0.1617399799145758  0.1651679559429210  0.5000000000000000
   0.2654238948326765  0.2302459666022875  0.5000000000000000
   0.4648491441023455  0.2302957310380670  0.5000000000000000
   0.5034166085918390  0.1651871355875907  0.5000000000000000
   0.6151129502837273  0.2250006124711290  0.5000000000000000
   0.6690957845937188  0.1686021559681546  0.5000000000000000
   0.7791844068846822  0.2240865148730268  0.5000000000000000
   0.8341281628102254  0.1682586355062796  0.5000000000000000
   0.9448977766286900  0.2240888493815248  0.5000000000000000
   0.0011901673239794  0.3347604182931989  0.5000000000000000
   0.1128002480312343  0.3899795195341308  0.5000000000000000
   0.2781696218000181  0.3923578701845382  0.5000000000000000
   0.3292699680819482  0.3330947249625515  0.5000000000000000
   0.4375917261378304  0.3751836103535961  0.5000000000000000
   0.5038746797115412  0.3331339125006332  0.5000000000000000
   0.6142141370098712  0.3923844732713641  0.5000000000000000
   0.7771869993619245  0.3899816316891282  0.5000000000000000
   0.8335708380417018  0.3347580486344631  0.5000000000000000
   0.9450673767920248  0.3901364395725873  0.5000000000000000
   0.0002854089656897  0.5005737086408080  0.5000000000000000
   0.1125245122684932  0.5546696956729279  0.5000000000000000
   0.1710342101699155  0.5004739714283358  0.5000000000000000
```

0.2839785680342323	0.5565367164310001	0.5000000000000000
0.3432413939975910	0.5022094217443872	0.5000000000000000
0.4594423034352175	0.5592293310860380	0.5000000000000000
0.5997916418595599	0.5592343023172377	0.5000000000000000
0.6589888958547351	0.5022313116276533	0.5000000000000000
0.7725724788870622	0.5565473933158394	0.5000000000000000
0.8294478762524425	0.5004766931510570	0.5000000000000000
0.9421513006558098	0.5546726347518135	0.5000000000000000
-0.0016364660183537	0.6654369863360233	0.5000000000000000
0.1098723763497007	0.7197375482755363	0.5000000000000000
0.1670801100178236	0.6654395191603358	0.5000000000000000
0.2783500404570707	0.7207344077933594	0.5000000000000000
0.3358263985459083	0.6666882184114982	0.5000000000000000
0.4462785158129143	0.7220479390450525	0.5000000000000000
0.5041601910717509	0.6684679524154200	0.5000000000000000
0.6142902343631766	0.7285829241162127	0.5000000000000000
0.6643153757544547	0.6684653952417376	0.5000000000000000
0.7757692694772207	0.7220425268358929	0.5000000000000000
0.8308657876625517	0.6666921147318968	0.5000000000000000
0.9423842532899477	0.7207397418170830	0.5000000000000000
-0.0029137589455960	0.8312292653411436	0.5000000000000000
0.1079529401739249	0.8848713839961554	0.5000000000000000
0.1652940333573225	0.8305781712104690	0.5000000000000000
0.2769217680967250	0.8848821169233022	0.5000000000000000
0.3341491752560543	0.8312379144812247	0.5000000000000000
0.4457425847634548	0.8877435207619571	0.5000000000000000
0.5020854023111161	0.8331125826557703	0.5000000000000000
0.6129393504746511	0.8904832639236165	0.5000000000000000
0.7775434425637228	0.8904770924722635	0.5000000000000000
0.8310228547176384	0.8331056095056484	0.5000000000000000
0.9419921070894021	0.8877362328176871	0.5000000000000000
0.3276993376311533	0.1555397144516455	0.5000000000000000
0.1678397967787325	0.3355309203878721	0.5000000000000000
0.6676967142267541	0.3355340881298319	0.5000000000000000
0.6682564163995287	0.8365100784302979	0.5000000000000000

```
Nv-NFC-2
   1.00000000000000
     14.7600002288999992         0.0000000000000000         0.0000000000000000
     -7.3800001143999996        12.7825351581000000         0.0000000000000000
      0.0000000000000000         0.0000000000000000        20.0000000000000000
   C    F    N
    65     1     3
Direct
  0.9975507019719081  0.9977732723873319  0.5000000000000000
  0.1074332454398942  0.0528381289516207  0.5000000000000000
  0.1646735131783899  0.9989390673970072  0.5000000000000000
  0.2721117823526852  0.0451480565598206  0.5000000000000000
  0.3349472404188258  0.0005891584751545  0.5000000000000000
  0.4459974895813026  0.0570391711514771  0.5000000000000000
  0.5005820750817483  0.9997053019524008  0.5000000000000000
  0.6110707244870746  0.0546961994022743  0.5000000000000000
  0.6648570943087203  0.9953796567741645  0.5000000000000000
  0.7756200036451996  0.0526940425862541  0.5000000000000000
  0.8301019637578186  0.9959165763063563  0.5000000000000000
  0.9406581350239169  0.0528138102714525  0.5000000000000000
  0.9952230344007397  0.1646436704150061  0.5000000000000000
  0.1051509082309710  0.2219664533401338  0.5000000000000000
  0.1585846469330140  0.1635502277313679  0.5000000000000000
  0.2610502238799747  0.2362244535225942  0.5000000000000000
  0.4798641944873712  0.2466842363311084  0.5000000000000000
  0.5036211422144309  0.1687642382606102  0.5000000000000000
  0.6159439408374651  0.2301378833385937  0.5000000000000000
  0.6671112207247759  0.1688106633872963  0.5000000000000000
  0.7770876049536324  0.2234303388310719  0.5000000000000000
  0.8306342794386096  0.1650468578507647  0.5000000000000000
  0.9408684181856266  0.2213615308073021  0.5000000000000000
  0.9973268134431550  0.3329121033384138  0.5000000000000000
  0.1079149035186463  0.3889299362388638  0.5000000000000000
  0.1630505010514364  0.3337068181421818  0.5000000000000000
  0.2740395410030154  0.3899154331419552  0.5000000000000000
  0.6170209402187434  0.4101301592556172  0.5000000000000000
  0.6702535591645618  0.3446793804378717  0.5000000000000000
  0.7794694461789422  0.3933723576504352  0.5000000000000000
  0.8327033358431066  0.3345023804907210  0.5000000000000000
  0.9431234448857242  0.3893551985473959  0.5000000000000000
  0.9999048260745448  0.5007458119291326  0.5000000000000000
  0.1108302539193012  0.5557249959576108  0.5000000000000000
  0.1654129786720607  0.4994277875366576  0.5000000000000000
  0.2764820767717922  0.5539782881165340  0.5000000000000000
```

0.3347776681374271	0.5009094406314302	0.5000000000000000
0.6711811650203811	0.5235789509696810	0.5000000000000000
0.7799719548641618	0.5602539405448580	0.5000000000000000
0.8346187464463256	0.5036027130804100	0.5000000000000000
0.9456361063989570	0.5574426275709792	0.5000000000000000
0.0031496829861472	0.6676375029406357	0.5000000000000000
0.1132594492305956	0.7224125090203993	0.5000000000000000
0.1683117265480382	0.6668228503529398	0.5000000000000000
0.2791590879954779	0.7215064926786042	0.5000000000000000
0.3340459580151168	0.6648505123398730	0.5000000000000000
0.4447107472790187	0.7164557190757369	0.5000000000000000
0.4955156861787858	0.6536780484462528	0.5000000000000000
0.6092440331466490	0.7029038575838653	0.5000000000000000
0.6700265061247951	0.6481055913827303	0.5000000000000000
0.7786662721302269	0.7180145820552271	0.5000000000000000
0.8403917694799579	0.6700432098192831	0.5000000000000000
0.9482623021224209	0.7234683922747128	0.5000000000000000
0.0014274897396485	0.8342447547411377	0.5000000000000000
0.1112345319865912	0.8881062249036377	0.5000000000000000
0.1683930443870041	0.8337501552077592	0.5000000000000000
0.2788529706358643	0.8880378100237396	0.5000000000000000
0.3349613114097907	0.8324826995374069	0.5000000000000000
0.4458737471479197	0.8868094965151028	0.5000000000000000
0.5003162454774418	0.8290173194308286	0.5000000000000000
0.6103750273844911	0.8817649891842837	0.5000000000000000
0.6638804319182380	0.8206360707479314	0.5000000000000000
0.7746563703926754	0.8823998253830183	0.5000000000000000
0.8318275127279595	0.8292837528319391	0.5000000000000000
0.9430094790704939	0.8862683131392108	0.5000000000000000
0.3267126852480012	0.1581130391508476	0.5000000000000000
0.3216933124964889	0.3299827676701660	0.5000000000000000
0.5104807140359435	0.3395337942368613	0.5000000000000000
0.4397799455577588	0.5484685707140079	0.5000000000000000

Nv-NFC-3

1.00000000000000

14.8037004471000007	0.0000000000000000	0.0000000000000000
-7.4018502235000003	12.8203806571999994	0.0000000000000000
0.0000000000000000	0.0000000000000000	20.0000000000000000

C N F

61 3 1

Direct

0.1063221597658905	0.0667848344127566	0.4999970457742167
0.0487930547855115	0.1219910855723396	0.5000025927390330
0.2740516899100898	0.0630729283872109	0.4999914955162845
0.2182787627129828	0.1210172070767629	0.4999911257276533
0.4431265164867743	0.0545621343006559	0.4999921613489715
0.3872375090361924	0.1119589401074529	0.4999893179960735
0.6120904869686966	0.0481783683811482	0.4999967435168414
0.5565314277664234	0.1072144153174692	0.4999983552588311
0.7808911376322072	0.0437260894685306	0.5000059962386550
0.7238672470254016	0.1005989589385931	0.5000024031874378
0.9417597285965498	0.0558485811875620	0.5000006249438937
0.8881368020474255	0.1101876717497461	0.5000093497028003
0.0631760131070110	0.2295517084692628	0.5000038619336696
0.2790991494665144	0.2337400818009459	0.4999918018623210
0.2566778384681986	0.3177696161000257	0.4999999304058351
0.4459581223206246	0.2225515121398759	0.4999903066661321
0.3898984482841770	0.2782364100857223	0.4999886884604132
0.6139708312895066	0.2195384413191656	0.4999976576888239
0.5573378361774011	0.2779382419694889	0.4999977003604387
0.7838418849595786	0.2156437645751650	0.5000064893375382
0.7263993796021267	0.2729310343648756	0.5000047866762504
0.9557446916891504	0.2181215822763863	0.4999986369496475
0.8987331649017508	0.2750893922017454	0.5000032619376152
0.1529032326838439	0.4206479917782179	0.4999988305991676
0.0645720444808059	0.4440975723483420	0.4999981123254188
0.1614855592009146	0.3255861713055277	0.5000030340144392
0.4385421202094896	0.3865980716475018	0.4999946934961231
0.6094164476485738	0.3897824646356896	0.5000006796924917
0.5488278496786924	0.4497068548146810	0.5000003606755986
0.7799752932632871	0.3854143376532621	0.4999994880912789
0.7216916293320217	0.4418452042388897	0.5000001609347907
0.9517257798024316	0.3869552331927243	0.5000000698471548
0.8926633120882178	0.4424869669195540	0.4999974786280033
0.1124278482242289	0.5570895883098874	0.4999955461885016
0.0560529074848336	0.6138551870953759	0.4999998182008797
0.2178539432267991	0.5839500724553711	0.4999927219776035

0.6118204848276320	0.5630475961094794	0.5000009694975301
0.7774633806423897	0.5536405520423454	0.4999978370816466
0.7221245463901232	0.6090812993704621	0.5000018171885163
0.9456421617086820	0.5556727073231684	0.4999960554657830
0.8879187167929963	0.6117029918029160	0.4999943844943117
0.1049295221429792	0.7270829887943882	0.4999929959330884
0.0471051560909599	0.7831452148744384	0.4999967234277374
0.2110569960859842	0.7886149457740737	0.4999874780030054
0.4150358029233011	0.7828089380010772	0.4999948402748554
0.6725626311451024	0.8384180626266490	0.4999947837869012
0.7659045839592579	0.7191415869192066	0.5000043689546119
0.6729248231090408	0.7355726469361816	0.5000014077246716
0.9363041322161795	0.7242292433693004	0.4999935407013553
0.8768583095224424	0.7790569597056950	0.5000011659665752
0.1029739244291084	0.8957952314249825	0.4999978975121213
0.0458623563793087	0.9520852971110100	0.4999969096480275
0.2718087485825079	0.8946844180987316	0.4999998231272804
0.2154542039996346	0.9520959644373204	0.4999990170607720
0.4411833661112231	0.8879451568476930	0.4999981862879991
0.3848116859645113	0.9440088953153989	0.4999922298397038
0.5782682842074829	0.8477721436080452	0.4999950850787518
0.5544389884301056	0.9355843653129561	0.4999978198057272
0.7682572332145359	0.9341457352912377	0.4999969011249272
0.9311009426762562	0.8914254395331399	0.4999970185305708
0.8755283260798743	0.9483171092298005	0.5000001886593068
0.2455555291102060	0.5149369422006222	0.5000013227732478
0.5845690125528110	0.6397750644697752	0.5000032849647948
0.4831563587757177	0.7553413312651235	0.4999966774563619
0.2986779926053421	0.6977525566068825	0.5000478287290103

Nv-NC-1+PMS
1.00000000000000
14.7600002288999992 0.0000000000000000 0.0000000000000000
-7.3800001143999996 12.7825351581000000 0.0000000000000000
0.0000000000000000 0.0000000000000000 20.0000000000000000
C N S O H
68 3 1 5 1
Direct
0.0009129765066027 0.0020871522138979 0.4981272565831563
0.1118856197582866 0.0574815305649984 0.4969141546822115
0.1668753322050690 0.0004596825047184 0.4965483333449160
0.2777951743346460 0.0552414473987215 0.4938659093679977
0.3339276150518217 0.0004680026979434 0.4962991912415075
0.4459407093882968 0.0574824946097159 0.4964901109629064
0.5015522525448274 0.0020718348727060 0.4975439082181861
0.6124049377865213 0.0591921903901042 0.4972751318266564
0.6691581249174273 0.0036964805897744 0.4972198908897680
0.7800229013675805 0.0596527016867989 0.4976645678574066
0.8349725425955357 0.0037452232323884 0.4976153160670800
0.9472126614712860 0.0592543934717444 0.4979145068183827
0.0033558850324400 0.1708687708052911 0.4979155833950979
0.1147512172775028 0.2261409128881436 0.4960681035536757
0.1682260914968648 0.1689502330292356 0.4943147469881647
0.2789693670053802 0.2224153587613389 0.4881627422778445
0.3332787395816270 0.1663455485600226 0.4886180699463995
0.4436673662952476 0.2224703444206245 0.4880927238779146
0.5009945445550644 0.1689546731967326 0.4939113247927612
0.6117265770286395 0.2261266225346546 0.4956112536104009
0.6678784834910702 0.1708583119931951 0.4973841307581300
0.7792952430789409 0.2259731842776755 0.4990124485886465
0.8355858517471081 0.1708105732758091 0.4987569424546618
0.9470146966482998 0.2259468773634822 0.4992471274921634
0.0031173018215312 0.3363531348461731 0.5006250914932360
0.1161159056565111 0.3911847016451631 0.5005239700050654
0.2830593011523656 0.3911192098308320 0.4932699841724788
0.3349390394244222 0.3332937571741468 0.4818889451609999
0.4416119842118753 0.3831750661366232 0.4664054799113874
0.4985511131632938 0.3334557664959461 0.4820547973799132
0.6083016572468330 0.3911833352881369 0.4934198838083072
0.7753382900839256 0.3911834382491122 0.5005220319963559
0.8335357833065178 0.3363554576877186 0.5005380585970082
0.9459235509118151 0.3915794896676972 0.5014565791614831
0.0012298537231339 0.5021390513806043 0.5020342850056269
0.1140952665993864 0.5562354107574773 0.5021613768002658

0.1736371182404745	0.5012859884598257	0.5017549396932047
0.2873428050561889	0.5565737165043804	0.5021799415415624
0.3473093927482281	0.5014332316321839	0.5014736417593024
0.4657455302943395	0.5602707812328375	0.5018169049039520
0.5950181397481462	0.5604518492095866	0.5023680440113373
0.6544079579622227	0.5015020979919047	0.5018456888063576
0.7695934517862487	0.5566094987344511	0.5025911548297646
0.8279480364918658	0.5013014156979726	0.5019582747712251
0.9424543132187188	0.5562246597869822	0.5023270998819044
-0.0008865596488227	0.6670663722169746	0.5021460430831497
0.1112495139813220	0.7221394320530775	0.5014677772337289
0.1682920550135732	0.6670824810408150	0.5018929399317548
0.2802484354824657	0.7227132816224485	0.5010348884834884
0.3375420406804370	0.6675288779300171	0.5009717958500555
0.4476831254129779	0.7235384286343631	0.4979561759639707
0.5059127411030372	0.6697747649381831	0.4965541556911561
0.6156874988581549	0.7309272580872734	0.4958373226640300
0.6643831487843900	0.6699595639771115	0.4971675540967741
0.7763063680903162	0.7236942452873845	0.4987020617675251
0.8303723602500581	0.6675939343133339	0.5016843252081828
0.9427944235310592	0.7227055974832172	0.5016113088810874
-0.0011135401856259	0.8337643511354500	0.4999552216968408
0.1110905446943986	0.8893064641416236	0.4990237383127820
0.1669242037520211	0.8335674042118482	0.4998429194377804
0.2785191650893947	0.8893074660258004	0.4986912108842131
0.3352216091804255	0.8337680462254197	0.4993153344153232
0.4467708728890429	0.8904131368941511	0.4978657493967086
0.5028544465932028	0.8349573419323486	0.4973127923922516
0.6141524516413307	0.8929904191366500	0.4967807782665654
0.7792709142034645	0.8930640358422032	0.4971440527704474
0.8325611781169071	0.8350568402772841	0.4980137228249347
0.9440866993683596	0.8904770298712413	0.4986795150240071
0.1718772770649107	0.3368750741634038	0.4968317638549937
0.6653026055297702	0.3368870139013236	0.4967656504597955
0.6697489518286032	0.8390437713872834	0.4964462384015330
0.4733079563760503	0.4543249298139796	0.6849386431504803
0.5876349634512649	0.5098119278428073	0.6928459280000513
0.4338435781619284	0.4159952564734810	0.6177640654101572
0.4214948207345891	0.5016121839087343	0.7205008373822578
0.4357778053936527	0.3357052486777577	0.7206391379531573
0.4896483975846481	0.3524845854887760	0.7856437709395858
0.5601490949785183	0.4086577553147873	0.7718729568844567

```
Nv-NC-2+PMS
   1.00000000000000
    14.7600002288999992     0.0000000000000000     0.0000000000000000
    -7.3800001143999996    12.7825351581000000     0.0000000000000000
     0.0000000000000000     0.0000000000000000    20.0000000000000000
   C    N    S    O    H
    66     3     1     5     1
Direct
 -0.0003870723066481 -0.0012854935560117  0.4979412101604234
  0.1110164759999200  0.0558757391729883  0.4965118206043151
  0.1697246929878342  0.0036811415402565  0.4955997448527310
  0.2824580476843694  0.0631312373722907  0.4945654998502448
  0.3366295288661080  0.0044011911607840  0.4959881443264663
  0.4466924466165371  0.0593174831867620  0.4965085260745423
  0.5012977929106516  0.0002856342079867  0.4970962557769024
  0.6113323640608919  0.0546348615660474  0.4977209881184668
  0.6653683304693937 -0.0047180918377937  0.4977420844690971
  0.7762099107681488  0.0526057898775872  0.4988757796554210
  0.8308715573659837 -0.0044569319369220  0.4986764188445783
  0.9417934289964295  0.0528542865545919  0.4987293728214818
 -0.0030271192883651  0.1644063508352920  0.4987605850381529
  0.1083658075219847  0.2211476536050360  0.4966380106727229
  0.1652373461309414  0.1672461394385348  0.4951757379037616
  0.2764048055681171  0.2278621791627496  0.4905749991601575
  0.4510480683086826  0.2336866383270070  0.4947733939666597
  0.5020949764136856  0.1718081376232676  0.4964478106158416
  0.6124435265140289  0.2280515988460264  0.4977697441161224
  0.6666071307300266  0.1683058447352708  0.4984902202333790
  0.7764408683328385  0.2226139420415707  0.4995648538151147
  0.8313075980296220  0.1645697577803384  0.4997054658587470
  0.9418818527898746  0.2205253576642912  0.4997586390052173
 -0.0016575175502990  0.3326681057477000  0.4998196095134355
  0.1086633213214252  0.3880508227394664  0.4982612797013031
  0.1641838825869069  0.3318360312592560  0.4957195787192704
  0.2746924388639961  0.3842866478097955  0.4900698482666551
  0.6074397805718433  0.4001641534807966  0.4967943709340795
  0.6667937355459134  0.3410867681070217  0.4979794083092803
  0.7772592856239195  0.3915784329932557  0.4992750638842853
  0.8319253043345934  0.3340288893696497  0.4999202867511919
  0.9432413514141081  0.3893541024722733  0.5001819136022478
 -0.0001379968749866  0.5001215544901157  0.5000207704385320
  0.1111937802838650  0.5554442106196120  0.4991543796331592
  0.1656461073928361  0.4988825442051005  0.4978145128762872
  0.2775030749395334  0.5536380353959418  0.4953828789558365
```

0.3349824098197320	0.4993991285385624	0.4908299666205392
0.6687984715390407	0.5200782142485438	0.4967279243916632
0.7777304968970914	0.5589151469020249	0.4980129524649047
0.8335994410700266	0.5022313108154294	0.4992808469207307
0.9447220405659386	0.5571358104053858	0.4997557072743249
0.0032583680836960	0.6679314651750861	0.4990124512676304
0.1141674227038003	0.7240813127519690	0.4984909383833388
0.1689526825583692	0.6671962470436927	0.4986085577991747
0.2795224086677804	0.7221945492809417	0.4975673088833421
0.3343601020195035	0.6643658615297510	0.4961672412574834
0.4450951341139242	0.7166988032713300	0.4953544936304958
0.4954019051873699	0.6538229901645578	0.4930391800875377
0.6094526832463418	0.7032047114567624	0.4949422176702520
0.6696709219954936	0.6467732582801525	0.4957488752417868
0.7783995235250852	0.7176212854149758	0.4967398437743766
0.8401417237586284	0.6691386605864611	0.4978320197680615
0.9485835263238700	0.7241446251343164	0.4982870470202534
0.0038333265896470	0.8358641793922620	0.4975242797681377
0.1145377760993738	0.8922797473538051	0.4967371302804408
0.1701404106153014	0.8355411148358414	0.4972177271166783
0.2806789791613736	0.8915772924648073	0.4968207137044915
0.3354499068052076	0.8339566693126454	0.4971844703572351
0.4463407131565702	0.8881098130887758	0.4968614962192143
0.5007050478084268	0.8293321199555534	0.4964289700100090
0.6104888655690506	0.8817359192006506	0.4966830355180419
0.6640622348998475	0.8197248143914021	0.4964465574416313
0.7751810411619634	0.8821328407958882	0.4976152970894128
0.8325836243979662	0.8292103795345044	0.4973684753759371
0.9448802917726302	0.8877722050064123	0.4976565136926096
0.3373799787754662	0.1747708693319139	0.4924922867705130
0.3265058126011429	0.3321824284847282	0.4848495061178575
0.5035026269446815	0.3402520807582864	0.4952222308579671
0.4395685449417228	0.5485280360957283	0.4888763490394412
0.5704991601068387	0.5397872397315988	0.6954349779376870
0.6745449769040963	0.5667317111353454	0.7183368385312597
0.5604074487026736	0.5527035229285255	0.6242148852529588
0.5141463711853324	0.5745125706408732	0.7399321515679227
0.4941022532279336	0.4062176329918806	0.7006695135119719
0.4601424048730912	0.3786435391913111	0.7707483203714451
0.4528298936992108	0.4404992753194044	0.7819615194851315

Nv-NC-3+PMS
1.00000000000000
14.8037004471000007 0.0000000000000000 0.0000000000000000
-7.4018502235000003 12.8203806571999994 0.0000000000000000
0.0000000000000000 0.0000000000000000 20.0000000000000000
C N S O H
62 3 1 5 1
Direct

0.1072936824365073	0.0668375974689059	0.4984215623026278
0.0502181079602856	0.1236070914611635	0.4983112892231339
0.2747315693364388	0.0611040386848737	0.4984719670823229
0.2190241841957140	0.1200330503267479	0.4979977568933430
0.4438966953232165	0.0573193679447211	0.4986825813335291
0.3876759179575797	0.1132996860368876	0.4986352212898738
0.6115025254527404	0.0484934657090339	0.4987477925593898
0.5565412765788875	0.1087954779092196	0.4989528920542344
0.7809678681947902	0.0451180229798640	0.4986605191306055
0.7239367427061649	0.1016397021831705	0.4988728629859023
0.9425900272992915	0.0568264374740244	0.4986665860024048
0.8886742664229844	0.1115984580419960	0.4985027058707037
0.0658825329425062	0.2341993105863511	0.4976753458534266
0.2791567022130128	0.2338969835417945	0.4966135579985472
0.2592380741737261	0.3205145472545715	0.4949825699377473
0.4458320571286105	0.2237851275902200	0.4987251174725107
0.3897781906308512	0.2790816724714543	0.4972796495920029
0.6141063083626417	0.2211322015209970	0.4992176191899057
0.5571070400467818	0.2792555764431190	0.4991676115933690
0.7842888188283617	0.2168609804631613	0.4990201218352848
0.7263860565612722	0.2743660438965447	0.4991776931459675
0.9565563129561954	0.2199496216575152	0.4980449657203328
0.8992273801455015	0.2765017633782578	0.4986540563142102
0.1608994574060923	0.4330513970645863	0.4963440273818575
0.0647689341130828	0.4485293959363449	0.4981192160656369
0.1661294944317357	0.3322501031224133	0.4965160558440591
0.4369870510828848	0.3873044176242462	0.4967667574862364
0.6085385009660434	0.3911081776214855	0.4991192343796785
0.5478035568308732	0.4505057044975265	0.4982863982368588
0.7793749196394865	0.3865668473052382	0.4993120998480375
0.7209568585455061	0.4430335412411711	0.4993888498977911
0.9526205085290248	0.3891164078048043	0.4988099343776502
0.8920581712903581	0.4438648174072817	0.4992127313636479
0.1097766556541185	0.5586697425544145	0.4979849946851845
0.0542625390232610	0.6138834714710050	0.4987385954739996
0.2204469487401151	0.6056929439390645	0.4961694160002790

0.6113493892163958	0.5638739606153800	0.4984127078311030
0.7769899640648834	0.5547608298365235	0.4995324789521444
0.7218861117704487	0.6100680087093908	0.4993868825220389
0.9438506920350084	0.5562527487268122	0.4991431154499219
0.8875594450950161	0.6124856987159918	0.4993150292977067
0.1102165086936380	0.7257711710485855	0.4985451533403048
0.0519560156075811	0.7821769945807820	0.4989383885773265
0.2226803343389897	0.7776134084271025	0.4978079175695582
0.3948088525844590	0.7811467049931787	0.4964843242253336
0.6679627601062940	0.8370002938119884	0.4977745469310296
0.7669281994650126	0.7203052024506562	0.4992013330139370
0.6723657902539639	0.7365070935712159	0.4980570956191990
0.9392649969617132	0.7247882323480485	0.4991847316914818
0.8787081888649534	0.7794927678333951	0.4992621152797064
0.1052154257443776	0.8947222971367744	0.4988720427584350
0.0474207391390516	0.9517572462347165	0.4988915414764860
0.2742977742850972	0.8901572361499037	0.4982696752783747
0.2174606898212162	0.9481637584119004	0.4986421657695937
0.4414102870978861	0.8917069296820429	0.4978036953598739
0.3860790023456585	0.9466730293540930	0.4983094887230922
0.5673865345978947	0.8418215453845908	0.4970720031001689
0.5517529003397980	0.9365395027984772	0.4981434947207035
0.7653358622085751	0.9337874820797475	0.4985896127274259
0.9322051697706055	0.8919251288395690	0.4990933096928336
0.8755078325156800	0.9486770410868025	0.4988475482807744
0.2819236083833953	0.7185240732361163	0.4965187150060427
0.2478151199082489	0.5283125206779259	0.4946401238218275
0.5845318631403261	0.6406420857553984	0.4971270764628321
0.4717126002045419	0.7541598461873910	0.4956804112031011
0.3895177686982095	0.4376738025453064	0.6814941365749497
0.3175817747313902	0.4291137786526249	0.7351032423466521
0.3502478737116345	0.4263244240543794	0.6135095391593953
0.4498184627223512	0.3868491135897938	0.6958925993475119
0.4764874007256990	0.5701446645571797	0.6805706127270531
0.4949575205316926	0.6096366097903801	0.7492773927187693
0.4244406332116885	0.5613016965116707	0.7679653100210431

Nv-NFC-1+PMS
1.00000000000000
14.7600002288999992 0.0000000000000000 0.0000000000000000
-7.3800001143999996 12.7825351581000000 0.0000000000000000
0.0000000000000000 0.0000000000000000 20.0000000000000000
C F N S O H
67 1 3 1 5 1
Direct

-0.0073195649276125	-0.0025633062089932	0.5116715876170967
0.1007983394397314	0.0506313975595145	0.5110676943937155
0.1614973583993204	0.0017760326184776	0.5069030170970471
0.2705538815437766	0.0751308269997853	0.4995417279852473
0.3322136153682217	0.0170551399491207	0.4931438690839470
0.4427196752677952	0.0619172414170231	0.4828424072435715
0.4973083143850835	0.0040465430940326	0.4903700816490726
0.6092632649592521	0.0592901401471780	0.4935637089246875
0.6647661891330764	0.0036304670474329	0.4994515457408263
0.7761900781943157	0.0590097940412775	0.5038422016891110
0.8303342259558645	0.0024981489592030	0.5076118771376222
0.9410335482436910	0.0565259845819796	0.5097158304149644
0.0014901747444609	0.1686023383275364	0.5071301063178765
0.1138274643902055	0.2216050602751649	0.5060134752719063
0.1641669586457193	0.1604263432778405	0.5080148740042715
0.2708805169769779	0.1893794415047693	0.5011404823401271
0.4720849982474906	0.2292015398800025	0.4319305847650963
0.5071082478041857	0.1715671338097179	0.4666872283033823
0.6149498891727385	0.2288614834239627	0.4859589250047042
0.6680134347289830	0.1717337608570991	0.4947349075148612
0.7785385676222002	0.2258296049272400	0.5000152655611861
0.8342741950343188	0.1699708336358588	0.5033711172622873
0.9460531583959706	0.2241946183104476	0.5045913621935443
0.0042042347055994	0.3352886316749026	0.5027888367091086
0.1163914868296223	0.3882577758734189	0.5016676761547318
0.2834855437024591	0.3786338448033081	0.4961790314136582
0.3317556099299489	0.3079417573007078	0.4880094749511224
0.4300765831705824	0.3577991691399052	0.4634116400952466
0.5062090257614942	0.3319252504353456	0.4565516148550022
0.6083069624199368	0.3907239152012106	0.4845698221573065
0.7767198199089966	0.3916145690712151	0.4972412102269829
0.8341486430140953	0.3367757396683803	0.4997336310059325
0.9468386960888618	0.3914321474651192	0.5016107558195843
0.0016864543104906	0.5019083240296829	0.5014392497967158
0.1147261092354252	0.5557582278239800	0.5005244609460150
0.1738513563896728	0.4995055452194641	0.4999519717830931

0.2872294450066032	0.5528038720554398	0.4985854263706939
0.3482954815588662	0.4933112358506871	0.4977064463685835
0.4667684267498347	0.5578270206524366	0.4982595336536513
0.5904271227638656	0.5588079273912903	0.4995626339303703
0.6500425690235945	0.5000050712515447	0.4985176673105161
0.7669219011141022	0.5553656533915068	0.5032969885068415
0.8280167678712732	0.5018920712728969	0.5012480652461093
0.9419209574916313	0.5564093900015962	0.5025904267784844
-0.0016107383837661	0.6680129761179455	0.5043322374805492
0.1110704824402389	0.7242607527961612	0.5021572014404505
0.1678780002909955	0.6681515263439421	0.4999821205238774
0.2784777296430110	0.7246777683666386	0.4979994361186025
0.3353965625999129	0.6659478935324334	0.4977970965519904
0.4452097667982360	0.7226756669441911	0.4978637075906793
0.5027978861847375	0.6679531308729255	0.4990053705909095
0.6124720271666318	0.7299424011673952	0.5021888860393401
0.6614740143830289	0.6686357258369098	0.5029572207996696
0.7719591134208168	0.7213918490769884	0.5070424134551037
0.8274709125285432	0.6657844627953310	0.5069009862158554
0.9404938628985462	0.7215719067880931	0.5071398342716315
-0.0041798659878924	0.8331676718257713	0.5087736482839241
0.1078508683425276	0.8902884283533266	0.5063876023986935
0.1658058212277177	0.8369263427985384	0.5022705857959362
0.2772709496068714	0.8989695997912812	0.4977630823636563
0.3322972528937423	0.8390064921424674	0.4963973579756857
0.4434085103468829	0.8927328534904316	0.4952272674796342
0.4993143182400502	0.8350898188997936	0.4978867870243249
0.6097039334064013	0.8922349437744884	0.5003210428599743
0.7745185182767881	0.8909125152287406	0.5073458189219398
0.8271189118267891	0.8325692491536600	0.5090556438824626
0.9380406889638147	0.8869599882419085	0.5106236117116764
0.3874486126340014	0.1829970924998953	0.3915149162329213
0.1727390429249752	0.3315354276781569	0.5015033966820248
0.6675274693766806	0.3378062062363578	0.4890743674777058
0.6654558399332072	0.8376701216935142	0.5036635145204805
0.4971309500368187	0.4807491754784896	0.6987816049313201
0.5971882450293777	0.5734823385619819	0.7133957846785329
0.4607874747576162	0.4717529442331715	0.6299368011039496
0.4167384186365240	0.4527572720298820	0.7507204294302363
0.5157085922319690	0.3753036489911148	0.7000946687517908
0.5189806927206686	0.3471552172266894	0.7707162655331951
0.4626752578307729	0.3590190070217050	0.7881088752853765

```
Nv-NFC-2+PMS
   1.00000000000000
     14.7600002288999992         0.0000000000000000         0.0000000000000000
     -7.3800001143999996        12.7825351581000000         0.0000000000000000
      0.0000000000000000         0.0000000000000000        20.0000000000000000
   C    F    N    S    O    H
    65     1     3     1     5     1
Direct
  0.0035037608263888  0.0032215689098840  0.5000457302424125
  0.1146924907478782  0.0591571155717171  0.5066036865268743
  0.1668801663448224 -0.0000440940959108  0.5153444414924867
  0.2728900786505968  0.0441479977163667  0.5345336793642032
  0.3334290682059254  0.0010763082920134  0.5137913357682976
  0.4454137957363079  0.0626425869056937  0.5056558086898413
  0.5004103715380700  0.0049890274045442  0.4994511021283927
  0.6107997822283260  0.0594390159964812  0.4946308483095481
  0.6660506495505708 -0.0004098790584309  0.4927985739432682
  0.7778985103367010  0.0564353445981021  0.4923712180239610
  0.8331284149673692  0.0001723691886165  0.4933869125009968
  0.9458564414353376  0.0573710105837073  0.4955324620318251
  0.0013716817308874  0.1680781715109919  0.4944611935731091
  0.1146293867414917  0.2241919716399950  0.4938734327259137
  0.1736446144228738  0.1712921215617484  0.4982508147647225
  0.2875103553182037  0.2367033694437075  0.4902518315294097
  0.4504303338667770  0.2439096986609488  0.4926947665527411
  0.4966513638859106  0.1747607674349370  0.4986008994359467
  0.6088804336624296  0.2313796657619653  0.4939942728185764
  0.6645402253680649  0.1722323016182931  0.4935261262237962
  0.7766390759876556  0.2259454989787220  0.4919039675971951
  0.8327577841940426  0.1685863803589761  0.4921349563542495
  0.9442310625112730  0.2237386829800856  0.4923100111245277
 -0.0007491764522059  0.3351395434247854  0.4909744117312227
  0.1113490089600512  0.3907261953076290  0.4900308459195901
  0.1683050451848969  0.3356334691423798  0.4897933864508130
  0.2788944514871907  0.3904029098691633  0.4867637087193377
  0.6097680275646726  0.4080223894425171  0.4909539759364177
  0.6652181789791008  0.3457974067335610  0.4916059689084443
  0.7761101987771916  0.3944447027339916  0.4908179337631544
  0.8314860645491593  0.3364342650972230  0.4910379697838138
  0.9437243109346019  0.3909979617375342  0.4905826537890298
  0.0001227068729948  0.5023422081465492  0.4904581100233655
  0.1113903116105509  0.5573022735240249  0.4906910944725122
  0.1667408624777239  0.5008830598849214  0.4899909845835217
  0.2787530901752383  0.5566204049518451  0.4896686746415013
```

0.3367131998509408	0.5035502378717125	0.4871405528083261
0.6684211655280812	0.5244091047783435	0.4906972777588643
0.7782288891742628	0.5621111244535083	0.4907616069910152
0.8323892582970198	0.5047654905524562	0.4904970544169865
0.9451232043626385	0.5590696387567651	0.4906750256460202
0.0027271410582820	0.6689954948365124	0.4919987744507572
0.1135569078358616	0.7240771445073554	0.4940102349495291
0.1682291190177418	0.6684355068985818	0.4923548134128073
0.2800680941301557	0.7245294570770242	0.4930151391543929
0.3351709890197708	0.6678627321017595	0.4913815574606615
0.4458784333153728	0.7214062159469009	0.4918099695922704
0.4967418206517888	0.6583639935300853	0.4895801015471987
0.6114722200870223	0.7078935378616017	0.4909219559251517
0.6698819423890437	0.6514663357419881	0.4911090099993186
0.7803417596410489	0.7221947035691924	0.4921732449572375
0.8403986608126902	0.6724921321204624	0.4915180157805325
0.9485640434327675	0.7260719732618407	0.4923084935754933
0.0028455826009216	0.8370572432681610	0.4960883791487742
0.1135624743219595	0.8912381425824475	0.5022699320716145
0.1687634466058553	0.8351856794765192	0.4994027153421502
0.2788943578275645	0.8907695156072636	0.5008186216898078
0.3350707635110569	0.8350393003635660	0.4961512662512829
0.4464477320367750	0.8912391932914555	0.4973107516354525
0.5007468451873949	0.8338979705055796	0.4940791186208310
0.6122042368534288	0.8869177077768449	0.4927934082146384
0.6659243422404324	0.8255610315133185	0.4920422749676701
0.7770888525713924	0.8870756881788633	0.4932019164899474
0.8342347067156414	0.8333431259554489	0.4941622747374154
0.9467353569367931	0.8908524912158665	0.4974389169388740
0.3123061458676071	0.1223289632384238	0.5803496869000316
0.3299207200566386	0.3351737206530838	0.4845309604845832
0.5028125729188475	0.3423469391278763	0.4891835025103713
0.4426124581592529	0.5543432599721549	0.4861372341785551
0.5625679827164675	0.5122497717946252	0.7036925655286876
0.6676028632762010	0.5415464377415515	0.7258020826693443
0.5479495772902551	0.5140403295132864	0.6319648863756444
0.5087848307166071	0.5521841752423698	0.7461570611040781
0.4867405986773711	0.3792253738515207	0.7171431954191949
0.4605499229155806	0.3623491225438276	0.7885823890948344
0.4545537030123446	0.4257963297606026	0.7969162667546406

```
Nv-NFC-3+PMS
   1.00000000000000
    14.8037004471000007     0.0000000000000000     0.0000000000000000
    -7.4018502235000003    12.8203806571999994     0.0000000000000000
     0.0000000000000000     0.0000000000000000    20.0000000000000000
   C    N    F    S    O    H
    61     3     1     1     5     1
Direct
  0.1050072591416876  0.0672708020189433  0.4958122603297605
  0.0460676814033472  0.1213498661726550  0.4934326794212246
  0.2734105128469492  0.0649456932076547  0.4967445438285581
  0.2170085961548282  0.1219033256597124  0.4976005283831966
  0.4440259465000935  0.0616005484478595  0.4942583303371095
  0.3874747632147632  0.1170938626314729  0.4962468979191462
  0.6111817236677219  0.0510491350021600  0.4924166771772051
  0.5569128848910753  0.1116264903657564  0.4938018760409196
  0.7778826590226591  0.0440382188019461  0.4910635082205026
  0.7227651538513096  0.1025313937897410  0.4921020188579931
  0.9384433269897131  0.0546160813425230  0.4911065732836462
  0.8853194469282524  0.1095849924010398  0.4902545306790685
  0.0603174965019957  0.2291844766716139  0.4945571965778409
  0.2777126888309592  0.2364324015650434  0.4983128094437704
  0.2537194987968859  0.3183375616220452  0.4998900961162016
  0.4459235844486781  0.2270773288605956  0.4968671438380067
  0.3894336448882434  0.2824380639133809  0.4978314201603218
  0.6146971364374817  0.2235903523338143  0.4946721933795002
  0.5577360641596202  0.2815909551339861  0.4961839629064054
  0.7835890531373981  0.2178105887391953  0.4927720810252254
  0.7269860881177698  0.2760457221484164  0.4940361195874736
  0.9530634056043497  0.2175561277971320  0.4922627904126589
  0.8982029834134370  0.2760984386528610  0.4930391619262609
  0.1508484993638116  0.4202660702934597  0.5109983370674688
  0.0635354551113382  0.4453855184048410  0.5030266843459748
  0.1574856245267867  0.3252544804596711  0.5017170935313807
  0.4394190897011155  0.3902070975812436  0.4992015762778552
  0.6105069306003862  0.3931541091208396  0.4969167113498952
  0.5502458211556580  0.4520670002483911  0.4983397998228882
  0.7812482209332314  0.3885520876506562  0.4955784972817300
  0.7230123352350722  0.4449667257526992  0.4965953809208844
  0.9524330133051755  0.3887967735868365  0.4960583944454250
  0.8938119523049075  0.4451399845456721  0.4972918521409188
  0.1112235239015371  0.5557112381545284  0.5165125679522088
  0.0589725707246226  0.6149875275103512  0.5067136774462448
  0.2116858399948326  0.5807087120028005  0.5417364058339285
```

0.6121467075904581	0.5643685342924321	0.4975558906211402
0.7790333120029794	0.5564780323823434	0.4975508562884332
0.7229001263325483	0.6113412456397256	0.4977354733974911
0.9477738201694711	0.5581846699923292	0.5002506292872051
0.8899917408683874	0.6137862718649412	0.4983504649815717
0.1123230151867981	0.7281275901473716	0.5007638565544749
0.0518598596059329	0.7839185348532044	0.4987672489916592
0.2218553212753627	0.7882923401367159	0.4940339511079945
0.3971577864481837	0.7891208686440707	0.4866216473837153
0.6639545795013193	0.8369436822420323	0.4921383056447822
0.7663737067316997	0.7210560280418796	0.4962724348444114
0.6702999185356175	0.7361718323040431	0.4944371304570249
0.9396662427228941	0.7260650850572207	0.4984932376671531
0.8781946412699263	0.7800117496866290	0.4967283073932992
0.1040177255276368	0.8964651012352604	0.4967414488426702
0.0451276222483108	0.9521724761022061	0.4957636681122117
0.2731966318529933	0.8961546951314439	0.4933853152480034
0.2161640003284383	0.9530049169184089	0.4958785546670458
0.4404980062085858	0.8972958888859440	0.4895128464374347
0.3864017915101537	0.9519348300399026	0.4919000459998716
0.5667867771024262	0.8452490457027343	0.4900360224107272
0.5515086384559676	0.9386183131194534	0.4906766773690060
0.7619826133239018	0.9330161447361567	0.4922314558519731
0.9302850212373968	0.8919117357611180	0.4951969256134408
0.8714715606010067	0.9470644769484542	0.4928592767092323
0.2363537344160202	0.5073265867245788	0.5352229027394482
0.5837811456166224	0.6403740579748282	0.4951123433257006
0.4628206881272063	0.7573274701922725	0.4873077210175125
0.2722388029249919	0.6642918960934128	0.5796953676972989
0.4030118488972885	0.4400947731754536	0.6807818336330173
0.3223527133525435	0.4370244897973478	0.7248026786943894
0.3746187994091265	0.4225429441464247	0.6097996421065071
0.4561554536567151	0.3871093473330204	0.7063881262849953
0.4914072237801608	0.5695517495485137	0.6758516051218280
0.5100635946289049	0.6137235269740710	0.7439960919702371
0.4366343757443512	0.5824394655825312	0.7588175512583185

```
Nv-NC-1+SO5+H
   1.00000000000000
    14.7600002288999992     0.0000000000000000     0.0000000000000000
    -7.3800001143999996    12.7825351581000000     0.0000000000000000
     0.0000000000000000     0.0000000000000000    20.0000000000000000
   C    N    S    O    H
    68     3     1     5     1
Direct
 -0.0011971517679784 -0.0010358381694681  0.4986317439544474
  0.1102272844427236  0.0556010371038482  0.4988435844683068
  0.1669344885548752  0.0008872873519425  0.4991412915705036
  0.2780538256597935  0.0570186096509897  0.4998618336304820
  0.3335619971349116  0.0007352065759512  0.4999006504687126
  0.4442705425240916  0.0577232587156974  0.5000690890350671
  0.4981050243516301 -0.0016476777792980  0.5004391745036194
  0.6084524089995643  0.0537854555864033  0.5012410032170288
  0.6634603385030854 -0.0037977322024121  0.5035617702169926
  0.7744065236057820  0.0527600930920413  0.5040900726891840
  0.8299151815698427 -0.0036026115431987  0.5027071179932925
  0.9414921030206966  0.0526462727395876  0.4997447588144434
 -0.0037535128260929  0.1644859479215840  0.4996964028148891
  0.1066411108193211  0.2197608433816566  0.4990737966359541
  0.1649315888089547  0.1669710300826953  0.4989937783953606
  0.2761989604212925  0.2231549778258383  0.4992446580226024
  0.3327369127602272  0.1693351861229356  0.5000716582866819
  0.4436474092537525  0.2308360494714389  0.5000681851078046
  0.4988746939135923  0.1720962152515122  0.4997572045252983
  0.6090336469780631  0.2244956060225795  0.4991507200918192
  0.6636963897468628  0.1663713487004362  0.5003230174803973
  0.7741007366906922  0.2203716176816857  0.5001849748218946
  0.8289016634067203  0.1633695530730719  0.5017453155841172
  0.9402024844402477  0.2192831093471587  0.5002791420542201
 -0.0042449243717896  0.3298957930146153  0.4993161444745789
  0.1066635278513672  0.3848623107033476  0.4988666244433571
  0.2675106256825658  0.3824604548686657  0.4981298713976480
  0.3291861171948245  0.3344338518593558  0.4991165058608585
  0.4387252993696178  0.4057918231063584  0.5004120762493480
  0.4983377117636483  0.3463104905358417  0.4987965984011545
  0.6093762101738681  0.3919526582938090  0.4916963763348903
  0.7739671702031730  0.3878733241188366  0.4957186074702923
  0.8290769466357164  0.3314126135075762  0.4982608675898958
  0.9408329993568774  0.3868409181733732  0.4987144541935600
 -0.0013967432304662  0.4980179440165892  0.4984192764073260
  0.1101181455034045  0.5525252366748075  0.4988642984149866
```

0.1651467910820340	0.4960923353292440	0.4988724399083988
0.2763678363635274	0.5506091684120238	0.4987797614582321
0.3291030497945803	0.4920919116247532	0.4972914113681241
0.4383133393213407	0.5294585012834944	0.4996270108203257
0.6218152116136895	0.5600560462403320	0.4634263381755748
0.6686685976271688	0.5021457589226419	0.4813086144962861
0.7791146917546120	0.5561865904346680	0.4893498005624458
0.8321126766390849	0.4990076951546144	0.4944314823208497
0.9435558100786385	0.5538388766259308	0.4971079269765701
0.0005737867720441	0.6656807713543574	0.4970657337320501
0.1116922636855509	0.7210026328859712	0.4985344107025588
0.1670711110033240	0.6642543742684762	0.4991587692455053
0.2779707905482213	0.7190391753327940	0.5002053314385340
0.3334479660498001	0.6616495035020966	0.5010444675508221
0.4433279636991458	0.7103507443667837	0.5029277170402993
0.4979149818402441	0.6482051620642041	0.5011128088314503
0.6071639980871779	0.7103919840266831	0.4944190692476551
0.6692302959220027	0.6653423488545397	0.4813596920680053
0.7797056273491846	0.7213082915078216	0.4905655425205530
0.8353413679518050	0.6671427925494996	0.4905247536569023
0.9460973584958976	0.7220157277125423	0.4951106653596312
0.0009389667863329	0.8330814069655773	0.4967260852350098
0.1118975311294937	0.8889367419832765	0.4983683082022444
0.1673247293537467	0.8327687909988960	0.4988938531297133
0.2785715000630957	0.8883995296612632	0.4996986557124972
0.3332065173061163	0.8308721232621011	0.5000471033682133
0.4437832468249972	0.8850315252135964	0.5002644227883196
0.4987192028475545	0.8253585670955929	0.5019782684538846
0.6072605158756230	0.8849160258898443	0.5042685585729000
0.7784284267538037	0.8864750923025264	0.5028114056700629
0.8330746067733057	0.8320072975099888	0.4979435242954672
0.9445373739853491	0.8881320283724847	0.4975370598537576
0.1604463218145488	0.3293955165608755	0.4987200840786282
0.6634077443849173	0.3350471879404244	0.4957773586305727
0.6633549602340325	0.8250134571668232	0.5183164230071760
0.5309930739062430	0.5314208928437991	0.6846764499450358
0.5753739769421211	0.5852113712912265	0.7473791069027388
0.6004215842829475	0.5668088150229627	0.6266740239636551
0.4220825909839376	0.5001465091437384	0.6721453270556257
0.5249732737367758	0.4016140106048786	0.6959508070392462
0.4768890298057994	0.3514071832060084	0.7508490916799074
0.6605014313846835	0.8169834016328196	0.5700177193135501

Nv-NC-2+SO5+H
1.00000000000000
14.7600002288999992 0.0000000000000000 0.0000000000000000
-7.3800001143999996 12.7825351581000000 0.0000000000000000
0.0000000000000000 0.0000000000000000 20.0000000000000000
C N S O H
66 3 1 5 1
Direct
0.0011085033163110 0.0000621132404267 0.4989099918511067
0.1128905305743519 0.0574344153906776 0.4996524763241632
0.1712326008413107 0.0048582533887782 0.4997490904404644
0.2836898407533929 0.0640868745331385 0.4989953180928862
0.3378753247645332 0.0052545371774485 0.4987115366787628
0.4480441894474880 0.0601137250434613 0.4981131248759239
0.5023815263239074 0.0012100336664981 0.4981084475942993
0.6126318503705066 0.0556049327571369 0.4976878569487923
0.6667220766591857 -0.0035442762851175 0.4976352080517973
0.7773703927697273 0.0533793029469328 0.4984526812914924
0.8323626719172003 -0.0031619725069330 0.4982047534439558
0.9433208832946101 0.0538707701912591 0.4991810519521138
-0.0017638676230289 0.1653059421236652 0.5002929859142760
0.1092224904780470 0.2217963919709753 0.5007207336543011
0.1669465789719341 0.1686680358108013 0.5002194013778266
0.2786967744253249 0.2297315800785155 0.5000416606181470
0.4526071248483219 0.2346237044770293 0.4951528263632242
0.5035699351163436 0.1727400093257681 0.4967590981250065
0.6137292930353683 0.2284516542320469 0.4967339526923497
0.6678350713539672 0.1688439757532028 0.4976307675372661
0.7778553292124850 0.2233013003293192 0.4989008604087969
0.8324554373448286 0.1654952201441386 0.4994448348570740
0.9430982504534849 0.2213505626591512 0.5003569259844621
-0.0011330267742796 0.3331667673498500 0.5007520394790064
0.1091533862918399 0.3886095511434401 0.5009572857618072
0.1640400049678518 0.3324712229129006 0.5010191329406735
0.2747137060218225 0.3849208597212338 0.5011338888967614
0.6099646481713686 0.4001020939250613 0.4933674758974247
0.6685093835065701 0.3414719396077735 0.4962432198401689
0.7789040266091745 0.3923568127165747 0.4978786884407712
0.8333855799437553 0.3347032431813119 0.4994116139735546
0.9441716168492729 0.3899936761326244 0.5005006159694694
0.0009484655999388 0.5011080086788210 0.5007876110701196
0.1122516428324872 0.5565172954085653 0.5009864688088278
0.1659671066151788 0.4994052398014828 0.5006954385678427
0.2772293660259977 0.5539031672832675 0.4998615467605081

0.3316073389911111	0.4978238864295672	0.4999275466910860
0.6710958795452343	0.5185760109080051	0.4930266178907195
0.7795081610886986	0.5595035678279401	0.4954103297190938
0.8351682651406702	0.5027417118013950	0.4976763458772409
0.9463183525677036	0.5580652219510863	0.4995940214472689
0.0045103697938303	0.6688829958423619	0.4996218250001286
0.1151830972632142	0.7248044961423112	0.5003942369078831
0.1698587931586466	0.6678940558371379	0.5006558182080169
0.2808643806566749	0.7228291564672905	0.4998913582633217
0.3350637439765485	0.6652554209594026	0.4992085213740696
0.4464191308606135	0.7176886515743839	0.4976170406324807
0.4987105684208579	0.6575611936954399	0.4969735545189732
0.6111994437169114	0.7054674655724533	0.4952672367820762
0.6715976115517974	0.6499614493424343	0.4935827722405213
0.7799162435139934	0.7188822165508661	0.4950370907573309
0.8413396174815143	0.6700880472120021	0.4962006185518253
0.9498682534002532	0.7251995097621086	0.4982754896388988
0.0051162083848928	0.8367919885963542	0.4986532841924207
0.1159859378289764	0.8932887046909690	0.4996857120486600
0.1713492343671847	0.8365959011904121	0.5000313607181021
0.2821549478441089	0.8925799366718190	0.4994957567870089
0.3368364188488994	0.8348990211570078	0.4992787269514262
0.4473651542347724	0.8886900613670556	0.4983128520273846
0.5019204019822656	0.8301182898337126	0.4974061603558982
0.6119363761398177	0.8830772304540919	0.4970184040988407
0.6656815573962577	0.8222384879555520	0.4962205880204032
0.7769403992885455	0.8837450107143708	0.4968150976192787
0.8342791910454862	0.8308331153389594	0.4965868842782919
0.9463146913773489	0.8888468548309375	0.4979834623732700
0.3388261250779168	0.1762123084570570	0.4980115905760986
0.3287432169476381	0.3347262918724240	0.5012578619505952
0.5055746609660997	0.3410613760861008	0.4911845881100915
0.4378804432138559	0.5505611508379640	0.4983606748536347
0.5719988231343502	0.5468618413331302	0.6731971263021940
0.6721966171291772	0.5613361340595009	0.6954564937891695
0.5469051211831008	0.5205689249314613	0.6028363168014078
0.5374542187972813	0.6143533362501745	0.7025443828379324
0.4765536305081738	0.4136743829800810	0.7059281955265058
0.4603069141915379	0.4101637577023114	0.7707310487071184
0.4721815926734094	0.5053040025011679	0.5057924330879096

Nv-NC-3+SO5+H
1.00000000000000
14.8037004471000007 0.0000000000000000 0.0000000000000000
-7.4018502235000003 12.8203806571999994 0.0000000000000000
0.0000000000000000 0.0000000000000000 20.0000000000000000
C N S O H
62 3 1 5 1
Direct

0.1058351649006765	0.0641206838866144	0.4989684315166954
0.0493827426492129	0.1217291914672472	0.4989948824367104
0.2733860763699124	0.0576018666354186	0.4987682351327144
0.2175032515975991	0.1167380814604640	0.4982970692688944
0.4420898053951867	0.0531053517764447	0.4988403945497177
0.3859724684359349	0.1090566051770052	0.4985880529127697
0.6105091731067785	0.0453338815447993	0.4991429764094207
0.5550896599583877	0.1048726684742013	0.4986479545406225
0.7805630164754381	0.0431663121517541	0.4997478817932625
0.7228180517808563	0.0989484222431673	0.4992451890469820
0.9413358592110167	0.0546289457172990	0.4997806226712336
0.8880463352111861	0.1091810531794299	0.4995250191506421
0.0649059713123415	0.2315235403093631	0.4982029052136135
0.2777379292187490	0.2301924060124578	0.4970110729346847
0.2580103232011767	0.3174319256951151	0.4957837448618718
0.4440840936831578	0.2198091070037196	0.4979949718304783
0.3881165883759870	0.2748131215919080	0.4972187648566644
0.6126350763280544	0.2172520594832267	0.4983186838721858
0.5553292837319408	0.2752343687879411	0.4979694911393869
0.7832533998365613	0.2141844418112226	0.4989135866801040
0.7250286715073768	0.2711219904343402	0.4986315224011657
0.9565906712197079	0.2179478779011325	0.4985866348415122
0.8984969091698919	0.2738334784465426	0.4987142670325041
0.1603109977028451	0.4302700379010769	0.4967026093694121
0.0635590275493722	0.4453653339619271	0.4983859518262906
0.1662926735294536	0.3300083399352174	0.4969250386287172
0.4348729521853336	0.3832865629473240	0.4967787594777877
0.6065706055448181	0.3871220484726520	0.4980493135611423
0.5452686100314845	0.4451915142067491	0.4981721482861711
0.7778823333763145	0.3835287039495280	0.4987685384914096
0.7192575423659294	0.4398528581733918	0.4985060323640259
0.9514861240966392	0.3862477533196456	0.4988482495923315
0.8903725071338214	0.4408351112403463	0.4991207810325106
0.1079162420686953	0.5553479935465132	0.4985877798184461
0.0523677465345960	0.6105919189442776	0.4992286243256340
0.2187329950160598	0.6025843186893004	0.4970966932301080

0.6088385029383964	0.5583796274307739	0.4995722736175459
0.7746222682625363	0.5515072060109837	0.4987744522309425
0.7184444934665366	0.6066230933866351	0.4989943553566387
0.9416036317842696	0.5531386471429035	0.4993279936621045
0.8850835099158784	0.6094368195259050	0.4992200789741900
0.1086244492433508	0.7225662116121642	0.4991533968155359
0.0501197331273132	0.7789932498516254	0.4994021335732619
0.2211813727134440	0.7739141585637279	0.4987947109423634
0.3937656639989474	0.7766168873703335	0.4985560406654641
0.6678473959910376	0.8359351843464324	0.5001040469644931
0.7636548570636347	0.7175225741317059	0.4996355470570073
0.6747735629208246	0.7376290971470153	0.5003078494574839
0.9370499198910091	0.7221507762951731	0.4994154687860468
0.8771795785339566	0.7772886877771422	0.4995923176621523
0.1034333622303093	0.8914779342433748	0.4994227564870267
0.0461465933624550	0.9490240513089935	0.4995197479837979
0.2729007849814861	0.8867975395166134	0.4990274622965971
0.2161382358779958	0.9446792574763370	0.4991632613385596
0.4402994361621382	0.8874832111315426	0.4991477926953536
0.3844911057852316	0.9423615025190737	0.4990914318173565
0.5674097589541152	0.8399552282296875	0.4993502840448394
0.5506931394189126	0.9327398963845686	0.4994005088898907
0.7652525201973780	0.9321213968851018	0.5000896800030028
0.9304022502951523	0.8891967737526412	0.4998351424119875
0.8741083144341403	0.9464364936010082	0.5000790828966004
0.2800845851638632	0.7150299284003608	0.4979930748891308
0.2469192130470314	0.5259960212940138	0.4951370529033224
0.5860019397339162	0.6383168363027287	0.5005705288679144
0.4711205227063326	0.7501611278457277	0.4985392626046149
0.3924898055322164	0.4605843200666850	0.6649624159092216
0.2846098687721633	0.4107336249480101	0.6868741454631883
0.4142297416211276	0.5070794812448474	0.5985174810874018
0.4527232847701224	0.4127760483125427	0.6860658807647807
0.4525127443542364	0.5883942575594957	0.7122363475460861
0.4751709846412475	0.5835577096696526	0.7749745828867882
0.5127620672948159	0.6303853582357261	0.5051684543495043

```
Nv-NFC-1+SO5+H
   1.00000000000000
    14.7600002288999992     0.0000000000000000     0.0000000000000000
    -7.3800001143999996    12.7825351581000000     0.0000000000000000
     0.0000000000000000     0.0000000000000000    20.0000000000000000
   C    F    N    S    O    H
    67     1     3     1     5     1
Direct
  0.0075770094653141  0.0034412034052125  0.5025044663950249
  0.1204592881280861  0.0612275541429684  0.5072088030649101
  0.1853710570589116  0.0164426534360947  0.4943362649333499
  0.3058093672400146  0.0748833603820093  0.4868820752570951
  0.3404823146904410  0.0000940205930419  0.4823377939024034
  0.4498587257558715  0.0482489494927967  0.4799087437633730
  0.5048022888626944 -0.0046638222258671  0.4835076820444670
  0.6151013081255778  0.0546366069419987  0.4870633243129442
  0.6712645696480610  0.0003926885191885  0.4951715096715812
  0.7826572260936500  0.0580530389065096  0.4990204627438758
  0.8395686862396465  0.0030353349802886  0.5025470933233602
  0.9508839888402871  0.0586617805227508  0.5026039234596708
  0.0042522608381926  0.1711552377096017  0.4997929377726640
  0.1141595669818271  0.2276659537940451  0.5065345501076267
  0.1660995499475191  0.1708364247320975  0.5241004203402608
  0.2602621700237389  0.2283374031895985  0.5572771889228588
  0.4171987902025040  0.1859054120998177  0.4839064438057947
  0.4962258981955483  0.1578238485182871  0.4803584964104875
  0.6072818038897647  0.2193163461519224  0.4837654805546798
  0.6667722392656841  0.1665056049103937  0.4862915296554303
  0.7776805905105877  0.2226302051775069  0.4894666354615760
  0.8351478975290050  0.1680600900853104  0.4940753484759299
  0.9469833325967126  0.2245898469841118  0.4944590255722857
  0.0020333152597977  0.3353928321771531  0.4917886104725388
  0.1141710425479700  0.3901959657235895  0.4934530814916916
  0.2809687394544606  0.3893624838334067  0.5049702395033403
  0.3263293569202975  0.3304873731940105  0.5301826352426248
  0.4238116867477025  0.3529139636571869  0.5148295849381594
  0.4757518253151285  0.3045371997087294  0.4952056662694461
  0.5943614555066922  0.3758666051969934  0.4904983640564419
  0.7707799742907137  0.3864854318465387  0.4877648347742705
  0.8304754461809840  0.3333660787442606  0.4883130807101088
  0.9436632598096184  0.3897624486923756  0.4891514076044706
 -0.0005493144274746  0.5005543804685385  0.4885534531234371
  0.1135486475108894  0.5549772637372680  0.4880550513170451
  0.1735099805668761  0.5008585142963183  0.4896772172067413
```

0.2873266081273296	0.5536462578388990	0.4892184010486340
0.3494772827473170	0.4987577725336936	0.4922470182770246
0.4679355711025620	0.5562485265266834	0.4920173540880373
0.5897305830800829	0.5550913651450377	0.4918107021206542
0.6441882124017263	0.4919354308605658	0.4907281425586523
0.7646407611328623	0.5510570467453787	0.4904882199600851
0.8246542324972987	0.4980497976296858	0.4890073696609402
0.9400342035276139	0.5542514496476801	0.4885698293770085
-0.0004937194047887	0.6668782668098102	0.4885329395282658
0.1124778231809741	0.7230069780076739	0.4869000405297037
0.1689889992283399	0.6666041315936161	0.4867749110232842
0.2802332105041206	0.7199580781324315	0.4870155950167724
0.3382129477298826	0.6649019511351216	0.4894309722139181
0.4479792121968379	0.7192171896523226	0.4940688621609868
0.5070846071396254	0.6667129243692864	0.4957200440493935
0.6155074488143610	0.7258769865857606	0.4990886964598160
0.6642119414878477	0.6663095425789339	0.4966976425194847
0.7769601460948907	0.7207724278546837	0.4977515065235837
0.8299905996265676	0.6651192853166998	0.4929721908741062
0.9458836234190304	0.7237165742378856	0.4910846854673440
0.0069373247789835	0.8382171590131013	0.4927554315814872
0.1217775775407711	0.8982940973101377	0.4901228442189918
0.1706635426375899	0.8356005700263379	0.4864592522543205
0.2820231976382437	0.8886676750842765	0.4842888272596689
0.3368145950798255	0.8314439810593244	0.4853026822615019
0.4482862886282479	0.8848960162532545	0.4865542213340752
0.5047673883422474	0.8307974679915638	0.4935182861801313
0.6143581873450386	0.8906979740317843	0.4987260527736774
0.7858964872734742	0.8927157845514817	0.5040666996905041
0.8361549378324432	0.8344034913907207	0.4996303049112118
0.9498892055117752	0.8919599418227000	0.4969657101953790
0.3012129506133026	0.1831575623974294	0.5955219611367338
0.1701913591869425	0.3373094589868104	0.5020561913730731
0.6585503721762139	0.3298281029621831	0.4870684721635005
0.6712548974532980	0.8362171091522658	0.5204470739835795
0.5001888305902634	0.5125298338261092	0.7049938416868055
0.6068068733402491	0.6007418132248462	0.7080830676011441
0.4598383752902125	0.4754313122741675	0.6379898489958027
0.4265615789226550	0.5083833608672769	0.7545404706055922
0.5263447591867879	0.4028919231880030	0.7306710779447233
0.4638292744216770	0.3390129372576514	0.7763832516557758
0.6688422137619322	0.8355302924007139	0.5725659100726572

Nv-NFC-2+SO5+H
1.00000000000000
14.7600002288999992 0.0000000000000000 0.0000000000000000
-7.3800001143999996 12.7825351581000000 0.0000000000000000
0.0000000000000000 0.0000000000000000 20.0000000000000000
C F N S O H
65 1 3 1 5 1
Direct
0.0035263765447227 0.0026915530066455 0.4920686446739096
0.1149551648677071 0.0586181290181193 0.4855588558954174
0.1674804583930793 0.9992968895194903 0.4775150695327845
0.2719213985718643 0.0420495359112310 0.4580051150356970
0.3336555940354028 -0.0004651501011091 0.4785771668512654
0.4447702802557341 0.0612291252116846 0.4866864019152163
0.5000367975067923 0.0042181544781810 0.4932358213714187
0.6107712532343084 0.0586178545967815 0.4978361831461934
0.6661189058513709 0.9993804066795446 0.4992113415939776
0.7773422518459712 0.0555161210010931 0.4998458798586322
0.8331825465591755 -0.0005504976774871 0.4985883704401723
0.9454639834702641 0.0561070053015105 0.4968124471946482
0.0010383166139015 0.1670624478519319 0.4989467643404450
0.1137240766395003 0.2227318373529261 0.4994131774890247
0.1739932149986792 0.1704946167862269 0.4940068146665187
0.2886029086039879 0.2366649519721138 0.5000039105477199
0.4504722344466635 0.2439183046019593 0.4979185264217849
0.4963049924412789 0.1748102740082980 0.4935550704774768
0.6081964525401251 0.2303568558520817 0.4987999897083321
0.6645030462593967 0.1713126063761908 0.4994198080161673
0.7761239919506765 0.2247001994627782 0.5017816188204465
0.8321866826409716 0.1676989533834751 0.5010948457770045
0.9438005602823009 0.2225672812882428 0.5016982317261309
0.9986059653982176 0.3337295461674081 0.5044615052353738
0.1100159736857036 0.3889318533576935 0.5055756068157822
0.1664126248137885 0.3338869404149309 0.5042467729562992
0.2768842217752257 0.3884402405713227 0.5055695206158379
0.6105682708801684 0.4062294832772557 0.5013585147548325
0.6654585617465746 0.3445057733444408 0.5020476982966340
0.7763850082608890 0.3934966776655762 0.5044439113416452
0.8317058017075395 0.3357273071628580 0.5041662130820939
0.9429862407450790 0.3897384910231830 0.5054948106935301
-0.0001013087010720 0.5013178715966150 0.5065449188405442
0.1113499645686642 0.5562479112138359 0.5060648897420056
0.1662771321739956 0.4995794643589122 0.5060295131343748
0.2776584953948288 0.5548840562161028 0.5051962081527560

0.3327650552107335	0.4999596737570084	0.5060034767921819
0.6703606716855368	0.5228620776513179	0.5020220213888587
0.7790914120612762	0.5614061327161000	0.5036327427810877
0.8330920485979662	0.5038239165505756	0.5052582032468993
0.9452440361689813	0.5578848804402154	0.5059778206645553
0.0033585185981146	0.6683619712267919	0.5039377057355972
0.1137338164713113	0.7231129089747889	0.5018674601905108
0.1685749429079276	0.6673201183263806	0.5040473490458038
0.2804515815397745	0.7228644195100051	0.5024062177594417
0.3352066248903144	0.6668382189617582	0.5032395670643672
0.4465765971774980	0.7203824518399077	0.5009078948322615
0.4996516557523029	0.6606002297348468	0.5023987263004380
0.6114228378272472	0.7077663402941813	0.5006938381715852
0.6714784100507178	0.6516947221562278	0.5008156199205810
0.7805058470884647	0.7210998046244758	0.5003054612436009
0.8409086802054824	0.6718184412761692	0.5025377208333222
0.9491489585807547	0.7254899375943799	0.5021618873727712
0.0034879133599933	0.8364170224118658	0.4976203207713213
0.1138846739461148	0.8901648821252479	0.4923568607388513
0.1690425513750095	0.8344402887158462	0.4955773350401650
0.2793604922314802	0.8895737183751994	0.4924932644723836
0.3358940548788827	0.8343518417771848	0.4977295157375325
0.4463333604822802	0.8901729151612531	0.4954802033308593
0.5014847862971705	0.8328509526241664	0.4980627608438860
0.6122395688370706	0.8858516877503614	0.4987547740829182
0.6661892977761928	0.8257454222094287	0.4995235278537808
0.7774335162960222	0.8866007456061873	0.4984568388611811
0.8350254609456063	0.8332557917604287	0.4978630912523451
0.9466663833392515	0.8900000315275545	0.4949233432359318
0.3126157540764582	0.1209187419511720	0.4119996423726932
0.3302788501430989	0.3355447137176699	0.5045922189295021
0.5040004593092788	0.3430405935836864	0.5002436737950176
0.4396874148470577	0.5536857330707307	0.5056762243575869
0.5567384226240383	0.5212925822502957	0.6836252751998058
0.6620383775699167	0.5366273467825908	0.6865245982686484
0.4986595957923111	0.4735506578874240	0.6222699414217058
0.5383192074911609	0.5991027545191554	0.7150100114849937
0.4888240695259428	0.4017836219232712	0.7413733132295075
0.4506139552156154	0.4149426590053726	0.7969254722086775
0.4745016928221813	0.5097860543767544	0.5155439180047234

```
Nv-NFC-3+SO5+H
   1.00000000000000
    14.8037004471000007    0.0000000000000000    0.0000000000000000
    -7.4018502235000003   12.8203806571999994    0.0000000000000000
     0.0000000000000000    0.0000000000000000   20.0000000000000000
   C    N    F    S    O    H
    61     3     1     1     5     1
Direct
  0.1044382812291267  0.0625256813066655  0.4931810085467370
  0.0499203358570775  0.1222030776886716  0.4925961826115399
  0.2706991381198264  0.0536966675903870  0.4957909602087103
  0.2157361890767324  0.1139591743810935  0.4937790049109793
  0.4389153808239176  0.0462242792072574  0.4998937296640678
  0.3831707570196720  0.1035318734025836  0.4969600454728949
  0.6092801179777321  0.0426882249306348  0.4975499425627543
  0.5523966528967664  0.1006487050206948  0.4983861909567555
  0.7809069783978441  0.0423160520585135  0.4952197102624807
  0.7217803855059595  0.0968387350543442  0.4955362679167383
  0.9424763790375433  0.0554220341663325  0.4929598268002027
  0.8884876041023383  0.1093882429739786  0.4933123279962319
  0.0651882963023874  0.2322598113288142  0.4919296099921863
  0.2752688881637575  0.2272434115091088  0.4933825512438914
  0.2577540915729811  0.3164721274196134  0.4923436387579038
  0.4407967963727539  0.2147948073036468  0.4967204544291264
  0.3852851626704700  0.2707390971857787  0.4953256747925590
  0.6091475640776619  0.2130922467873954  0.4969997573358144
  0.5520035831243678  0.2713005676488138  0.4975193015917735
  0.7807144254087446  0.2116283074572488  0.4947774788911813
  0.7216850728111865  0.2674646106137473  0.4956154300449843
  0.9556604991201949  0.2176262722794743  0.4926037715438989
  0.8961956047185615  0.2724146988930693  0.4932171530315875
  0.1554341066360595  0.4287410971387674  0.4874385208450253
  0.0604170692517434  0.4431176425538038  0.4884418669085540
  0.1653587385469231  0.3312304073389037  0.4906104534572581
  0.4309552441816052  0.3792923543844473  0.4963939292991511
  0.6026825709307140  0.3833689445489607  0.4982564837833735
  0.5409841556373528  0.4422475844494649  0.4997614229869444
  0.7738475079108112  0.3798454012433602  0.4951031520855284
  0.7150586160684387  0.4362438283511714  0.4967314879835797
  0.9476756244089535  0.3838646350765350  0.4916775610642757
  0.8861539361078544  0.4376197183349871  0.4926584107890511
  0.1012901632272100  0.5537818111295273  0.4855983128506544
  0.0454420368057831  0.6073891978373902  0.4878787865245041
  0.2092216407352670  0.5977760066594677  0.4817112624208407
```

0.6054277061133214	0.5559982045506396	0.5024444128214898
0.7702270527278511	0.5481494769807052	0.4961485615034922
0.7145868902350575	0.6034899587673784	0.4987347750072504
0.9362132708727160	0.5500756187801509	0.4914663684258889
0.8799505009605428	0.6064246435352769	0.4936280588950386
0.1011210702093484	0.7204074012213321	0.4889333737079434
0.0439346898226855	0.7771145839131631	0.4917494745120777
0.2091126029467283	0.7718435032122946	0.4901458615825855
0.4151435598066480	0.7807904301217889	0.5388901234697199
0.6724314474954056	0.8380508183354956	0.5028998807470108
0.7609179283741331	0.7149178993654312	0.4986365049812803
0.6743492263064990	0.7363024612648771	0.5031295474880820
0.9318168489112035	0.7196955997335193	0.4933767254462936
0.8738571616933252	0.7754320917993583	0.4953488846810560
0.1003559542253172	0.8892058005289809	0.4930810673413630
0.0440116314072535	0.9475972579528293	0.4933725211217925
0.2691646129434624	0.8811799369259494	0.4977641597895185
0.2130055841015478	0.9413829760734210	0.4955560032441813
0.4414744164755732	0.8825087593945151	0.5155300098720983
0.3821414026901118	0.9348746868905816	0.5053760598964222
0.5780607405814768	0.8451301340938503	0.5107280878828252
0.5522668974542569	0.9309791686182629	0.5036853999236289
0.7683729994840149	0.9334108317459558	0.4964135827888266
0.9289008754818984	0.8879301902266151	0.4942742841073858
0.8754872139055639	0.9473013700927073	0.4942095769760066
0.2421728170585943	0.5318712303245920	0.4830900812418438
0.5845275590177543	0.6378598039997700	0.5058763442735401
0.4894820920902013	0.7572492035748634	0.5324105267585930
0.3299391679057516	0.7177187546183054	0.5745885118669162
0.4041434509795299	0.4869880128622331	0.6684603942534689
0.2999390244114626	0.4228564322241801	0.6955213114434112
0.4133919498396792	0.5485336414704338	0.6095009521169152
0.4759780824968231	0.4475923573433733	0.6740177250228232
0.4604892072099540	0.5996049765032420	0.7323522923491875
0.4883215172184057	0.5769436436625198	0.7887367788339993
0.5140033267096037	0.6315209290367685	0.5191400240623233

Nv-NC-1+phenol
1.00000000000000
14.7600002288999992 0.0000000000000000 0.0000000000000000
-7.3800001143999996 12.7825351581000000 0.0000000000000000
0.0000000000000000 0.0000000000000000 20.0000000000000000
C N O H
74 3 1 6
Direct
0.0002766513031479 -0.0003564237966965 0.5014509041676189
0.1129738440152607 0.0582855606035075 0.4998526008614994
0.1671081646100343 0.0016988391458658 0.4973220723457208
0.2789805038662855 0.0579228803096749 0.4954130822539022
0.3341083929885418 0.0020647153181775 0.4949565195742825
0.4456646103763147 0.0569950085498859 0.4954255060757879
0.5004945702146427 0.0002069393050311 0.4966049007756710
0.6113487040758068 0.0538968105405266 0.4978740317209205
0.6668935882727416 -0.0021323946261888 0.4987340685708531
0.7783300642115113 0.0540245051070031 0.5006503818085760
0.8326278282444718 -0.0020793331026486 0.5018824382915628
0.9449757839080063 0.0549302520275875 0.5023824484456857
0.0019703556569711 0.1672221279805413 0.5019926138767689
0.1147853678477180 0.2252475342166934 0.5012985576772726
0.1722745223176249 0.1725693658796244 0.5007313915415996
0.2856942806041716 0.2307334255392407 0.5007387399176673
0.3362862355958777 0.1703456149818357 0.4974673740056722
0.4466333841596822 0.2246847278846493 0.4965766276759869
0.5017940470883291 0.1685132812149860 0.4960834524464610
0.6131678515227756 0.2218502445371614 0.4973563906013037
0.6681042582706685 0.1660769140522278 0.4983884469818111
0.7789845665698023 0.2209285480151842 0.4998654595708730
0.8345680513567564 0.1653911152212417 0.5007999119460250
0.9459513618280069 0.2216155448232398 0.5012230433242456
0.0021091987402061 0.3330107027701657 0.5002102245345166
0.1134372933874599 0.3891558164309447 0.4981501985451841
0.2813304089446322 0.3921343794897276 0.4951269363027579
0.3454513600734451 0.3457233266145246 0.5021036459628747
0.4637399169187103 0.4051753788789317 0.5033811242746073
0.5049480738211903 0.3367827493095031 0.4974883580501499
0.6146209600497052 0.3855188963182761 0.4974751460971638
0.7780349890436482 0.3867164861640895 0.4994585124738021
0.8338151871398793 0.3317437631703293 0.5000828675179627
0.9460197992867303 0.3884737879609867 0.5003233386001729
-0.0003106922766243 0.4992726400610769 0.5000452137858379
0.1106206110453806 0.5549985102250617 0.4988174510795623

0.1669368839654608	0.4999243001771903	0.4966280278905276
0.2778492042057951	0.5572738081543406	0.4909000456139741
0.3337192967630713	0.5022394538029301	0.4849507343239587
0.4405279589805245	0.5590809295252835	0.4697214538860350
0.5950039508199026	0.5362717557094344	0.5045160870307248
0.6634716133902233	0.4951832423116617	0.4994163128327340
0.7754470742167611	0.5533144411657679	0.5002297574075909
0.8314450972820574	0.4980554005064138	0.4998924746758168
0.9429404212268162	0.5541520620871982	0.5000298836896796
-0.0021178681263563	0.6657225480237705	0.5000646376304563
0.1101450431119688	0.7224747359928969	0.4985398089372432
0.1658029101691758	0.6670153372869265	0.4973645617666724
0.2767085085919603	0.7231503828047329	0.4946417060768251
0.3322813111961931	0.6675744688662270	0.4907170530954238
0.4426215261072566	0.7219969516538756	0.4902686452997644
0.4976069937901325	0.6659654577557758	0.4847683073220517
0.6076792386704634	0.7184030363783458	0.4952205254970712
0.6541180520999353	0.6543904844946781	0.5031081859523834
0.7691738398568981	0.7140905408594516	0.5030426815945338
0.8295967157709052	0.6635184249770937	0.5020014318987520
0.9419571503611316	0.7207737308473426	0.5010410594235361
-0.0017601035372516	0.8327471590975840	0.5003338366081040
0.1102618943106222	0.8897380966561902	0.4981532098453038
0.1660298282642539	0.8340213560887659	0.4973491155600684
0.2775960885971837	0.8899389727708040	0.4959286178336903
0.3328537381012066	0.8341122953445321	0.4955555716441142
0.4448222605838718	0.8892306492377183	0.4962745571923504
0.5000132843197937	0.8330028837867997	0.4951300013076803
0.6107979976485650	0.8865434610008476	0.4970110881403871
0.7745733140919364	0.8850634538621830	0.5014093288332240
0.8272644165713624	0.8275532832442613	0.5020593142549112
0.9415894177641398	0.8869448908668430	0.5014004861965339
0.2772200483214466	0.0528094732615730	0.7059255804058955
0.3443362314157414	0.1446728810950655	0.7407423482782767
0.2105361397849557	0.9606547819084090	0.7405243828294891
0.3442782581143524	0.1438280375908868	0.8105446631553960
0.2112789558597195	0.9610216209066826	0.8102116032189063
0.2779136852356315	0.0523051920056913	0.8456011265425799
0.1701804310624268	0.3352353119390674	0.4984885820520369
0.6687117463199357	0.3312561403644672	0.4979345910070954
0.6645857516089997	0.8296308979442604	0.4982101582356327
0.2733496274861530	0.0482285905491373	0.6375715807471053
0.3962023697912879	0.2161289919031156	0.7131231309744346
0.1592783757875817	0.8903391922983662	0.7120279610822531

0.3966924794259751	0.2153831671996796	0.8376670778111491
0.1593094127360887	0.8891781232375136	0.8372234859458038
0.2781360119374041	0.0520609763869232	0.9001155303477437
0.3216991858667518	0.1166865209493627	0.6187757004217144

```
Nv-NC-2+phenol
   1.00000000000000
    14.7600002288999992     0.0000000000000000     0.0000000000000000
    -7.3800001143999996    12.7825351581000000     0.0000000000000000
     0.0000000000000000     0.0000000000000000    20.0000000000000000
   C    N    O    H
    72     3     1     6
Direct
 -0.0003157552424000 -0.0012247157879328  0.4962135255359958
  0.1110532723483234  0.0558410624864882  0.4942051281333968
  0.1697012841132188  0.0036349286804903  0.4927025874876180
  0.2824401557629011  0.0629484255523794  0.4919740621121033
  0.3366109905997215  0.0043046521139257  0.4938852366279955
  0.4466280369130602  0.0590832017713377  0.4959952841530519
  0.5012201519566690  0.0001090136712955  0.4980968026707007
  0.6112202488479656  0.0544488148721242  0.4994345933416302
  0.6653406871921206 -0.0047429353924755  0.4997406699161137
  0.7762386528887125  0.0525670682538655  0.4999070730382733
  0.8309494807295146 -0.0043832471460656  0.4988278761313295
  0.9418848036451366  0.0528807148149910  0.4979787192115037
 -0.0030191787475986  0.1643452560741456  0.4987420784758896
  0.1084262333278786  0.2211176884424354  0.4974147866916450
  0.1652260129856752  0.1672132661144315  0.4948221385296769
  0.2765941300942145  0.2281119870164137  0.4935571484009949
  0.4512476746156571  0.2334675279633842  0.4945071787048830
  0.5022086165240564  0.1715327196841283  0.4962737826533747
  0.6125478651625671  0.2277364802866174  0.4985732188165868
  0.6666252096214556  0.1680682114696726  0.4997897254063464
  0.7764783387260819  0.2224221285947698  0.5007074074569939
  0.8313519863050456  0.1644629640593698  0.5005433351556440
  0.9419014678075063  0.2204279538617444  0.5002886459802307
 -0.0017427104787772  0.3325832423313345  0.5008492781749105
  0.1085852483679483  0.3880486232135045  0.5002712203558517
  0.1641001087027210  0.3318372956472882  0.4986052824644494
  0.2747767471223481  0.3844036537619372  0.4973125014665868
  0.6078517181805460  0.3997654065828207  0.4975116140216042
  0.6669975341068872  0.3407450837244576  0.4990290891075231
  0.7774180282027295  0.3914127809162835  0.5002708170258827
  0.8319656176259563  0.3338370363556246  0.5009475038521897
  0.9432735133902561  0.3893093601455033  0.5012753695812779
 -0.0001159386096112  0.5000657224620025  0.5010601279556114
  0.1111294824353226  0.5554615237189836  0.5005341387608787
  0.1654924790008741  0.4988520359463525  0.5002839397167588
  0.2773889546369374  0.5535989357331170  0.4995939692894501
```

0.3349952756171350	0.4993862036599915	0.4985937942100855
0.6690524547242575	0.5196697430107731	0.4982850980941030
0.7780870445031979	0.5589483288552601	0.4992054507471385
0.8338142567651993	0.5021340354293546	0.5000457645345322
0.9448565327642438	0.5571370852866288	0.5002551051315471
0.0032802089665163	0.6679444641100388	0.4989312851960762
0.1141621727767940	0.7241306192039199	0.4982485097915654
0.1688293373200528	0.6671981643992850	0.4993607493294027
0.2793749694628875	0.7222614459983857	0.4989256077664876
0.3341577445090947	0.6644461885174605	0.4994162292861974
0.4448743707226430	0.7167972785248409	0.4991954413887982
0.4952779342636749	0.6540426140946575	0.4989084070313214
0.6093895701092836	0.7036036932591713	0.4986316410360677
0.6696311448142724	0.6470210943520923	0.4980849496237481
0.7784717073101777	0.7176612510312470	0.4978630742542069
0.8402909654577433	0.6691923833585457	0.4985146757816227
0.9487403146684549	0.7242667284275187	0.4980090452196157
0.0039797794775173	0.8359596686205616	0.4961288371948157
0.1146195331784952	0.8923555197154853	0.4945578123768175
0.1701229242107694	0.8355569780261864	0.4959409860592824
0.2806520834335437	0.8915827664233770	0.4955931657555542
0.3353617307019405	0.8339790342071284	0.4976007878107543
0.4462467807151160	0.8880613118360520	0.4982692892428433
0.5006139416072830	0.8293648411413671	0.4991228864131898
0.6104339658502383	0.8818049745300967	0.4993379547752236
0.6642094603224322	0.8200624882020665	0.4988930286285476
0.7753487561240293	0.8823114472270621	0.4982864445606393
0.8327126038488742	0.8293119169228885	0.4975564380037485
0.9450368478904284	0.8878547498767149	0.4964835090602494
0.3374860523832228	0.1747479844142296	0.4924953933902513
0.2769036783392628	0.0529384651155962	0.7051818746113523
0.3437306504660682	0.1445830599706454	0.7402582837133402
0.2100537563519945	0.9605662306101134	0.7393947570997492
0.3431913830203093	0.1430985738534311	0.8100486308331052
0.2100496338902269	0.9602742859949217	0.8090598963593142
0.2764934056933619	0.0512407668984144	0.8447377490067731
0.3270852571856603	0.3326604257449530	0.4946805308557676
0.5039384804803336	0.3401062661239811	0.4951736695700479
0.4397503540687966	0.5484954658382953	0.4983618204602222
0.2734659011312393	0.0487632604010830	0.6367450085782084
0.3958380824857946	0.2162410043956113	0.7129105956106299
0.1585622464176278	0.8906014431613154	0.7106428195466338
0.3955774908050426	0.2144491410042251	0.8373687208535845
0.1574274278817893	0.8882472620561370	0.8356466811361051

0.2765591844783018	0.0506043984754300	0.8992483322483151
0.3205113399429747	0.1178212246967432	0.6181634054174572

Nv-NC-3+phenol
1.00000000000000
14.8037004471000007 0.0000000000000000 0.0000000000000000
-7.4018502235000003 12.8203806571999994 0.0000000000000000
0.0000000000000000 0.0000000000000000 20.0000000000000000
C N O H
68 3 1 6
Direct
0.1068197951266738 0.0653824768487719 0.4976822716013777
0.0497243537654583 0.1221667598345713 0.4985780239655638
0.2742509226460693 0.0594232521362656 0.4967539921765080
0.2185571414699072 0.1183165901856822 0.4973923851262869
0.4435577812310024 0.0557419361293057 0.4970672610559915
0.3873116036057646 0.1116321516501461 0.4976574456608199
0.6112707011908887 0.0472522008389941 0.4983668033430136
0.5562044877572425 0.1073208489797622 0.4982080597424788
0.7804647282234809 0.0437624326859215 0.4992516707596855
0.7236043847791843 0.1004255048052746 0.4990259479553354
0.9421558388162793 0.0553794953514157 0.4992254272844171
0.8881224777254118 0.1102091879916021 0.4993444893955395
0.0650975804661584 0.2325429256175058 0.4984243908883573
0.2787708655141728 0.2322769732227313 0.4981223597668598
0.2581886232641131 0.3184240628844964 0.4982439446480318
0.4456075046509060 0.2221459952079685 0.4988865839788529
0.3895624839443593 0.2775349002980792 0.4988224494666522
0.6138764522953751 0.2195834934972441 0.4990897087752512
0.5569148021264618 0.2777296770593022 0.4994144850192697
0.7839492469845900 0.2155343039006266 0.4991395391538762
0.7260812290242290 0.2730111498083740 0.4993188618710530
0.9557415909110426 0.2183710953370000 0.4987693926578174
0.8986948348910445 0.2751498891043760 0.4987538837379893
0.1603197466787426 0.4314437586459186 0.4968839348371375
0.0644617366889915 0.4470269298794150 0.4965864251258010
0.1652367592564604 0.3305701206256244 0.4978514943261657
0.4373781015142987 0.3857356049298724 0.4991900717124019
0.6086179543090181 0.3895719627346309 0.4999394420564024
0.5478925641189218 0.4492499002666360 0.4999785117211326
0.7792758366079252 0.3851154622850890 0.4992661307506615
0.7209517690050790 0.4416549974620245 0.4998089854975457
0.9523282702376255 0.3876752221390323 0.4979164885600955
0.8919161401243781 0.4424190256433221 0.4981675074243471
0.1094137616122066 0.5570522456041772 0.4949888866892338
0.0540264470134267 0.6124807474189903 0.4949152070997003
0.2202082818371421 0.6040732901516456 0.4947062332253775

0.6112789136393565	0.5626789337226744	0.5003892333693817
0.7768101771216448	0.5533370310329606	0.4996015649926309
0.7218237978041331	0.6088337546139341	0.4999949402834056
0.9436254424436212	0.5548273287108788	0.4971027431036965
0.8873372307158878	0.6110836755588200	0.4982068272270108
0.1097495917003677	0.7242927306500928	0.4935794514402316
0.0514564096780412	0.7805553208528746	0.4954078801313198
0.2222576351650972	0.7763150558273803	0.4926077001038869
0.3945905204228189	0.7799124964534300	0.4953476150701493
0.6677734986418378	0.8356402691784335	0.4990076902937254
0.7667146880534705	0.7189636639893491	0.4994921006116858
0.6722379677309744	0.7352258311800118	0.4996668635122017
0.9389067130269592	0.7233777857560478	0.4974739811384592
0.8784894766728401	0.7781830703996722	0.4984802969769984
0.1048122721452351	0.8930965991788701	0.4958277434599725
0.0470455744818926	0.9502763816218344	0.4973996606941521
0.2739212522826376	0.8886920010096167	0.4936761217428269
0.2170286784974874	0.9465735072916840	0.4951518957773599
0.4412718317548675	0.8904922470925233	0.4959182925270899
0.3856985933282044	0.9451294842982630	0.4952844230042223
0.5672177153913217	0.8405229295036389	0.4978680471322265
0.5515793540714656	0.9353507170184615	0.4975760738508750
0.7650030683312631	0.9324852732638764	0.4992819330248559
0.9319270953377472	0.8905982503568962	0.4983635954592694
0.8752707103440001	0.9474572981122084	0.4991430678822410
0.2816787969837737	0.7170928294170974	0.4938923130213394
0.2025745591512983	0.7601981014690529	0.7056206695792091
0.2677777361921947	0.7339907436583633	0.7410847104490480
0.1363329712939304	0.7867716671510121	0.7393982826447735
0.2663578456546546	0.7346220962043305	0.8108529321104667
0.1358624419327980	0.7871078053637433	0.8090730613329636
0.2006351489802383	0.7611086168264657	0.8450951759534360
0.2476904777407488	0.5266589037049506	0.4959891443028495
0.5842628253738527	0.6393267067723548	0.5002725666451178
0.4716013489440924	0.7528910071731938	0.4966667945440527
0.2002416938716821	0.7612354952454248	0.6371208851533008
0.3190345749007953	0.7132278565063985	0.7140566415478162
0.0863424370253834	0.8067662933287838	0.7104112164520351
0.3170734532530127	0.7140719852783726	0.8385094701845653
0.0842671947764573	0.8077864680914282	0.8355500606596095
0.1998977240790900	0.7614168917145625	0.8995927347502579
0.2482038496488249	0.7400684362582448	0.6192347828022683

Nv-NFC-1+phenol
1.00000000000000
14.7600002288999992 0.0000000000000000 0.0000000000000000
-7.3800001143999996 12.7825351581000000 0.0000000000000000
0.0000000000000000 0.0000000000000000 20.0000000000000000
C F N O H
73 1 3 1 6
Direct
0.0046869014361630 0.0017216641745184 0.4928482365270293
0.1178010536048655 0.0564627576848644 0.4863221787623694
0.1629576807830884 -0.0097069053334907 0.4766200370593620
0.2643891055346353 0.0286022887184159 0.4526036051134960
0.3275285625498012 -0.0097764517414029 0.4765160762942425
0.4388313986495264 0.0563808707585220 0.4862647522985308
0.4972472561466730 0.0016328093489477 0.4927518794462519
0.6080366026933132 0.0582326706233317 0.4959588135822986
0.6674464317838320 0.0032094539111619 0.4968329913201035
0.7792853677372916 0.0584267142266294 0.4975494827276788
0.8359406759769556 0.0032053235929865 0.4969768744550240
0.9504401748237052 0.0582924384511333 0.4960725927193694
0.0092603168599878 0.1691820797850979 0.4984627692903323
0.1242163171308270 0.2231962598374027 0.4992568756530005
0.1851000083876067 0.1675513455467911 0.4939517462039582
0.3096037120202017 0.2291030553828085 0.4968922621204774
0.4195404390765629 0.2290681976769046 0.4972342519414589
0.4826216655374633 0.1674603503616294 0.4939870592237214
0.5991924329140896 0.2231596700342405 0.4993077730614350
0.6600891512761429 0.1691405320568568 0.4984050489254414
0.7749082363308956 0.2232639076226889 0.5016176400908019
0.8346649072716035 0.1691470138650936 0.4998189023697026
0.9485392201460588 0.2232942807407058 0.5016502213843967
0.0026722004356719 0.3327902184389988 0.5069278735805086
0.1166291491890266 0.3876131537356917 0.5090398041999694
0.2851216880414383 0.3902384914529497 0.5051549369031522
0.3427310784548854 0.3389148006425581 0.4938506271994300
0.4495335488679053 0.3989063938538416 0.4778913743563337
0.4963291785947047 0.3390072245222026 0.4941424094541735
0.6052983630920662 0.3902834839877110 0.5053945041562400
0.7712032983805384 0.3875955268358147 0.5090752807569545
0.8303189523261264 0.3327602799832300 0.5069209781438622
0.9437407393013548 0.3872531679100325 0.5092152132572176
-0.0009283617281147 0.4979232594217018 0.5114705114536440
0.1122159477212945 0.5522006030385296 0.5118089164592623
0.1726953106739965 0.4974035894715845 0.5118327473186796

0.2866998771660633	0.5548711532626226	0.5128488456952863
0.3464343011676159	0.5003578575682628	0.5133932142382703
0.4656107825459525	0.5599128390361098	0.5134547762258570
0.5947440775338521	0.5601520995792967	0.5140554528121359
0.6541886606615203	0.5004141852375953	0.5137250248915939
0.7684158941038747	0.5548784714962879	0.5131003020951987
0.8249144007534472	0.4973770714346185	0.5119427517157122
0.9401920077432711	0.5522120024870967	0.5118882782592696
-0.0031993421004903	0.6626822181685156	0.5102503416474021
0.1085509481662058	0.7168993511817973	0.5076487855508107
0.1660974012402562	0.6626842180454449	0.5101643523473138
0.2789460263982575	0.7200201892163630	0.5081192159463136
0.3368147722750209	0.6656765190923644	0.5094159073534611
0.4469573575540116	0.7228806652242038	0.5037670365939557
0.5059550264703959	0.6694736794451269	0.5038182417066751
0.6153493468767272	0.7304861440439191	0.5007557997204055
0.6638148765366698	0.6695699364457239	0.5043428853662428
0.7762168893293818	0.7229437708901502	0.5042799403066800
0.8290808027079676	0.6656915068928040	0.5097406442752992
0.9412769541743675	0.7200277691585950	0.5083135082241335
-0.0026988878271292	0.8301302295419940	0.5017016575608333
0.1087216329155191	0.8831233766542275	0.4948004498727412
0.1637177268762728	0.8271809132684289	0.4989975353704976
0.2746296466556607	0.8830668101267551	0.4946782914022412
0.3330816583653098	0.8301017013521115	0.5015103169243519
0.4446136905870501	0.8894604786119308	0.4968120868552467
0.5015061712009968	0.8344364145307783	0.4989956042663401
0.6134337350363858	0.8928868707234058	0.4973239303658700
0.7797429166233356	0.8928866114310275	0.4975675467841258
0.8332517313715258	0.8344651667819517	0.4993776002135651
0.9451661888479144	0.8895390053648425	0.4970533267819098
0.2800082263218953	0.0568518551375539	0.6971958549478763
0.3472817881872905	0.1481374301822204	0.7326977403210349
0.2132069844555697	0.9645441343829096	0.7318194606713215
0.3468450876689439	0.1464149818611445	0.8025637173442920
0.2137527314059944	0.9638903078008340	0.8015397544780833
0.2803747362849877	0.0546089589970014	0.8374462994822404
0.3028735645395237	0.1055424007671324	0.4056046633050815
0.1743059165774668	0.3336696092326357	0.5056237466104311
0.6595616524950246	0.3336748609742571	0.5057322690418931
0.6697826408095777	0.8392713160780191	0.4982705117827223
0.2756184502088293	0.0524263145059348	0.6286400599711003
0.3992830713922009	0.2200677850813178	0.7056473624857645
0.1613976500953904	0.8945342968064288	0.7031909133678218

0.3990704898398872	0.2177166281619109	0.8300019526460204
0.1612322651557672	0.8917224280400780	0.8281108754531865
0.2803804831089530	0.0537819025669784	0.8919825455815540
0.3213881424732327	0.1217451785086505	0.6095513673299198

Nv-NFC-2+phenol
1.00000000000000
14.7600002288999992 0.0000000000000000 0.0000000000000000
-7.3800001143999996 12.7825351581000000 0.0000000000000000
0.0000000000000000 0.0000000000000000 20.0000000000000000
C F N O H
71 1 3 1 6
Direct
0.0030957875616225 0.0005251908505844 0.4921404809045617
0.1139678926445115 0.0579077613501020 0.4845184847843905
0.1748764004738104 0.0067700558200505 0.4919677156678550
0.2864193326793294 0.0605008490164985 0.4983676785941079
0.3360834662470585 0.0023614284264299 0.5020091513530275
0.4484667428458802 0.0554757923439093 0.5056790659617104
0.5020169828970127 0.9983758700529559 0.5061590175719076
0.6124751570417942 0.0536059436005576 0.5065918539842839
0.6667377884516095 0.9940312309564387 0.5052007490006231
0.7779965023323996 0.0520192973138459 0.5039563029524280
0.8328369786584183 0.9958493835489112 0.5005597378313996
0.9447692802238756 0.0538957309786748 0.4959707267953933
0.9984253157896941 0.1647801484405137 0.4970117998158363
0.1090846159289343 0.2223103902057680 0.4869315510228691
0.1638439798406301 0.1687325032560183 0.4730684448379774
0.2643279014145286 0.2351078990332082 0.4434279545063152
0.4528897033271839 0.2274863095161757 0.5064663064271769
0.5015491189116880 0.1655153982925306 0.5069968199911332
0.6121105746166621 0.2254115715268336 0.5078308596194683
0.6664960184606518 0.1664241455121189 0.5074610151165809
0.7774788010329561 0.2218148804298789 0.5073496253585004
0.8323242646363178 0.1643516428840347 0.5053545179407440
0.9428804366549334 0.2206538233876595 0.5030625995610591
0.9978755418729218 0.3323028520100348 0.5041532238528299
0.1094672234604904 0.3885103930526873 0.4995828399293724
0.1645254996647793 0.3337701657865342 0.4882974093826679
0.2738959195329506 0.3877152086008446 0.4765893678231075
0.6051158960311984 0.3980518602554080 0.5062384283064350
0.6640332290520542 0.3392800645848273 0.5074652970108564
0.7753145603967437 0.3901834861373780 0.5076021253231627
0.8309174524439726 0.3326395683682000 0.5078954324961721
0.9428912751503084 0.3883012562738175 0.5071084428907169
-0.0006528508239599 0.4998795514895723 0.5068296029733951
0.1107115222302141 0.5552065198117954 0.5052949475064832
0.1654445272961999 0.4985114440926910 0.5017879045910125
0.2772210233300441 0.5527252893614414 0.4986636056374885

0.3343136056073026	0.4993766695908918	0.4891332156140257
0.6653048566249995	0.5166471326737654	0.5045561241635361
0.7763625881664442	0.5569637893334981	0.5045079559105108
0.8314312962172096	0.5007833265151520	0.5062348345871380
0.9445621781891133	0.5564746681485820	0.5059384641679294
0.0028717208552134	0.6669851968269888	0.5034444169560336
0.1139498940821293	0.7231181069297445	0.5032058830708690
0.1678643933679504	0.6664894297629576	0.5046104775524463
0.2797354495731968	0.7212570582225813	0.5041205249551410
0.3343165830577668	0.6637956993375476	0.5022644032157229
0.4449905516558887	0.7159033930751824	0.5018343494484337
0.4952128740236443	0.6525979706262214	0.4983710838205921
0.6101501624303128	0.7023348363454869	0.5016468799393736
0.6687443181563367	0.6468599696676312	0.5025462960400796
0.7796367304917081	0.7177000196268142	0.5015600171109592
0.8397310915446902	0.6683588319615422	0.5022618303253170
0.9488597816270486	0.7240026361694418	0.5009795304072923
0.0049441626130953	0.8361247938526577	0.4972593165336071
0.1175106143833281	0.8937689911989537	0.4965256029476630
0.1706652974286439	0.8356537811994919	0.5008787302687276
0.2807976584139235	0.8898146648066367	0.5021891709328922
0.3355347233392634	0.8319767611313199	0.5041428555816846
0.4468049422900108	0.8854895244099196	0.5050163403641494
0.5006813878332460	0.8280594701914038	0.5040663797902503
0.6122500137698444	0.8812891829638717	0.5039747997099872
0.6656733696356203	0.8204586674568430	0.5025021168086983
0.7769570850033251	0.8826270387560876	0.5008676521460442
0.8343622957558031	0.8293405739598888	0.4997048114483401
0.9471740806028164	0.8881623418288377	0.4966734351792745
0.2782375195599491	0.0543203766319203	0.7093038641999708
0.3463691842512964	0.1453261330789547	0.7446097078638783
0.2107295156921794	0.9621974820727193	0.7437681558050112
0.3461779923806229	0.1435509116917185	0.8144804700154497
0.2113510946676059	0.9616548690566800	0.8134532921029540
0.2787270201386254	0.0521181663581026	0.8492693537740021
0.3011418781822607	0.1906591449428836	0.3993628348350225
0.3184976807372410	0.3358772449095035	0.4478134749487244
0.4954915732896920	0.3261507736469472	0.5070757507296986
0.4404048528807977	0.5488689106782684	0.4902505730166903
0.2732273000089332	0.0491598402434371	0.6409269684635632
0.3988895664857709	0.2169605536636872	0.7173991881577423
0.1585208446930432	0.8924228621000839	0.7150803856120385
0.3991773762250504	0.2146388637998599	0.8418116097251331
0.1583932512782479	0.8897222282800950	0.8400725304237838

0.2788010318315007	0.0513021201862735	0.9037803291155584
0.3204047906479199	0.1169001085199802	0.6206530188910088

```
Nv-NFC-3+phenol
   1.00000000000000
     14.8037004471000007         0.0000000000000000         0.0000000000000000
     -7.4018502235000003        12.8203806571999994         0.0000000000000000
      0.0000000000000000         0.0000000000000000        20.0000000000000000
   C    N    F    O    H
    67     3     1     1     6
Direct
  0.1062027428865596  0.0640731300732321  0.4953189529777637
  0.0470892147068155  0.1180358522641795  0.4942043578573471
  0.2747331911488983  0.0618699531084728  0.4952098317446030
  0.2181844951646630  0.1185068430262226  0.4970768831941206
  0.4455870366296897  0.0588766868542639  0.4931335322428538
  0.3889226314921375  0.1140361572567443  0.4955863563658794
  0.6125081835192158  0.0482917047577341  0.4922597295273042
  0.5584043588076036  0.1088845458284332  0.4938447515135316
  0.7788831976014916  0.0409095314384433  0.4917727064264878
  0.7239550349929270  0.0996182683019713  0.4927694851846419
  0.9395445986636243  0.0516142631304988  0.4921761980294855
  0.8863720871906650  0.1065436964695073  0.4919806704969339
  0.0614126219670269  0.2258052463326035  0.4959178766668477
  0.2791627819923187  0.2330795616855119  0.4999538027395634
  0.2543187269224759  0.3139782284349449  0.5028946963031921
  0.4476268803769856  0.2240028754134402  0.4978081277043678
  0.3910279414809845  0.2792089873502522  0.4996308169861541
  0.6163612515758665  0.2206737613193788  0.4956898230799097
  0.5593544608593435  0.2786199978138131  0.4972976671007115
  0.7850259284513476  0.2149105583101044  0.4939658515422313
  0.7286871588435230  0.2733110297301067  0.4951497829076922
  0.9542271719293607  0.2144922797290990  0.4940841883845983
  0.8994937180843574  0.2732067759030932  0.4942619399809909
  0.1518220042615976  0.4165421979323843  0.5116086176108842
  0.0652123865689235  0.4422653172586859  0.5026612327134721
  0.1584975705987345  0.3218416112494423  0.5029301981417947
  0.4410336317042653  0.3868609010330086  0.5003378693232610
  0.6122278179043201  0.3900934806764308  0.4977178446659926
  0.5518162284794026  0.4494695063958684  0.4985233574407718
  0.7830602402925416  0.3857885935758116  0.4960323587062316
  0.7247805872385988  0.4420775912455610  0.4966943898427623
  0.9538724028904324  0.3858459105777634  0.4961261271642383
  0.8956127101294722  0.4423465705488618  0.4968548831306996
  0.1133695423293384  0.5531357616762959  0.5144944070325312
  0.0610455843888325  0.6124538235989201  0.5037328238070884
  0.2137979532246848  0.5780044260039332  0.5394389036394481
```

0.6139251797278390	0.5617064617620601	0.4966068252431967
0.7807883667228160	0.5536322869376398	0.4960035278019567
0.7247044336904301	0.6085660629794443	0.4956470327078381
0.9496774399200686	0.5553784063710689	0.4982692354743391
0.8918960146195127	0.6110773644998122	0.4960216141135483
0.1139673897450149	0.7253414640107421	0.4965495677772107
0.0535279676906188	0.7809120814443689	0.4952964903402592
0.2233927379113113	0.7857621908817130	0.4890948881446956
0.3987211676759680	0.7865968964950392	0.4827992682221497
0.6653074269616045	0.8339526512662687	0.4910083279077166
0.7680402896279771	0.7181850353075239	0.4941553464013629
0.6720068080578671	0.7333848915353177	0.4932213144171162
0.9414602942823871	0.7232923336463246	0.4957018193597013
0.8798861803491518	0.7771858921191962	0.4944567907247771
0.1055396163602498	0.8934647675567303	0.4939490717001790
0.0465543861757954	0.9490951107779393	0.4942134635362360
0.2746589944329887	0.8934109397744069	0.4891666648668653
0.2175590734970520	0.9500436518871327	0.4928851075330406
0.4420712819209100	0.8947600278450429	0.4864269390448596
0.3878947669869493	0.9492545048388411	0.4889463933857470
0.5682267321596239	0.8425561774162783	0.4882721306958581
0.5528809306647765	0.9359597063284198	0.4892212347760341
0.7632701191603722	0.9298650821307627	0.4918397687655539
0.9316982620652869	0.8889573595936786	0.4937433213106908
0.8726927581791396	0.9439915147615575	0.4926179123002411
0.2008329744535516	0.7771604826921247	0.7056529240829008
0.2649860014705806	0.7455739173656535	0.7373418457188506
0.1357133119769783	0.8007602300753192	0.7434247496798571
0.2635836903488769	0.7380544249966124	0.8070185139484537
0.1350177248429729	0.7928322440589717	0.8128912247885052
0.1988723686720525	0.7616176101513350	0.8450515510407390
0.2377580042869649	0.5040367158528859	0.5354399222485977
0.5854113353425964	0.6375443571457001	0.4949767247689594
0.4642285074036429	0.7545973204867775	0.4844461028201750
0.2773087832644335	0.6635429381450086	0.5761562357815155
0.1986954761427865	0.7872849790792020	0.6376186989508228
0.3154268810771209	0.7268549346187601	0.7076443461469242
0.0864731223740724	0.8248796315562547	0.7174259866686582
0.3135195408528623	0.7134582170915520	0.8316645422136009
0.0838665272588183	0.8109512128943062	0.8421559231594937
0.1980181624148917	0.7555701737858899	0.8994192404001936
0.2397749779321001	0.7601822625313233	0.6164262508542011
